# Experimental Design for Stochastic Models of Nonlinear Signaling Pathways Using an Interval-Wise Linear Noise Approximation and State Estimation

**DOI:** 10.1371/journal.pone.0159902

**Published:** 2016-09-01

**Authors:** Christoph Zimmer

**Affiliations:** BIOMS, University of Heidelberg, Im Neuenheimer Feld 267, 69120 Heidelberg, Germany; University of Edinburgh, UNITED KINGDOM

## Abstract

**Background:**

Computational modeling is a key technique for analyzing models in systems biology. There are well established methods for the estimation of the kinetic parameters in models of ordinary differential equations (ODE). Experimental design techniques aim at devising experiments that maximize the information encoded in the data. For ODE models there are well established approaches for experimental design and even software tools. However, data from single cell experiments on signaling pathways in systems biology often shows intrinsic stochastic effects prompting the development of specialized methods. While simulation methods have been developed for decades and parameter estimation has been targeted for the last years, only very few articles focus on experimental design for stochastic models.

**Methods:**

The Fisher information matrix is the central measure for experimental design as it evaluates the information an experiment provides for parameter estimation. This article suggest an approach to calculate a Fisher information matrix for models containing intrinsic stochasticity and high nonlinearity. The approach makes use of a recently suggested multiple shooting for stochastic systems (MSS) objective function. The Fisher information matrix is calculated by evaluating pseudo data with the MSS technique.

**Results:**

The performance of the approach is evaluated with simulation studies on an Immigration-Death, a Lotka-Volterra, and a Calcium oscillation model. The Calcium oscillation model is a particularly appropriate case study as it contains the challenges inherent to signaling pathways: high nonlinearity, intrinsic stochasticity, a qualitatively different behavior from an ODE solution, and partial observability. The computational speed of the MSS approach for the Fisher information matrix allows for an application in realistic size models.

## Introduction

**Fig 1:**
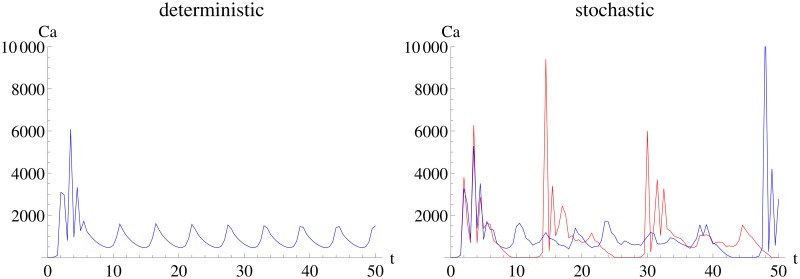
Deterministic and two (red and blue) stochastic realizations of the Calcium oscillation model. The left panel shows the deterministic behavior of the Calcium oscillation model, the right panel two stochastic realizations showing the special characteristics to which the experimental design methodology in this article can be applied: qualitatively different behavior from deterministic modeling, bursting oscillations, high nonlinearity and fast dynamics (e.g. from almost 0 to 10000 molecules within a few time units).

Computational modeling is widely used to deepen the understanding of biological processes. Due to advances in experimental techniques (e.g. the possibility to measure small numbers of molecules in single cells [1]), the importance of stochastic modeling is increasing. This article focuses on experimental time course data that shows intrinsic stochasticity such as e.g. signaling pathways (Fig 1). Stochastic simulation algorithms have been developed for decades [2] resulting in a lot of variants today [3]. Recently, the development of parameter estimation techniques suited for stochastic models began. These techniques can be classified into approaches based on the chemical master equation [4], moment closure methods [5–8], Monte Carlo methods [9, 10] and approximations [11–14].

The development of experimental design techniques for stochastic models in systems biology is a very new field. The goal of experimental design is to *design* an experiment by choosing the experimental conditions (e.g. time points of measurements or components that are measured) in such a way as to maximize the amount of information that can be obtained from the data. In contrast to parameter estimation, experimental design is independent from measurements and can be calculated before performing any experiment. Therefore, it is a tool to reduce experimental costs by obtaining a certain predefined accuracy or maximizing the accuracy with a predefined cost.

The most common quantity for measuring this information for models of ordinary differential equations (ODE) is the Fisher information matrix [15, 16]. As the Fisher information matrix is a parameter dependent measure, its application needs some prior knowledge about the system’s parameters. As this is not always readily available, techniques such as robust experimental design [17, 18] have been developed to broaden its applicability. The Fisher information matrix is under regularity condition the inverse of the asymptotic variance of a maximum likelihood estimator [19]. Therefore, the Fisher information has a high impact on analyzing and improving parameter estimation: it is used to calculate confidence intervals for the parameter estimates. Furthermore, it facilitates the investigation of the correlation between parameters and the optimization of experimental design. For experimental design the Fisher information matrix is first mapped to a scalar by so called optimality criteria [20]. This scalar function can then be optimized. The optimality criteria reflect the experimenter’s interest on the parameters. Additionally, optimality criteria can also be used for model selection. This article suggests an approach for calculating a Fisher information matrix in stochastic models.

The Fisher information has been applied to ODE or differential algebraic models [15, 21]. A review of experimental design techniques in systems biology is presented by [22]. The mitogen-activated protein (MAP) kinase cascade is investigated in [16] who compares the confidence ellipsoid of the Fisher information to parameter estimates gained from inference on simulated data. There are also approaches to experimental design without the Fisher information matrix such as Bayesian experimental design [23] or design strategies based on profile likelihoods [24].

Current approaches for calculating a Fisher information matrix in stochastic models in epidemics [25] and systems biology [11] approximate the process with a multivariate normal distribution taking into account the inter-temporal covariances. [26, 27] uses moment closure techniques to compare experimental moments to parametrized theoretical moments. Based on that, the authors show how to design optimal experiments to investigate gene expression. [28] suggests a Monte Carlo based techniques to derive optimal perturbation experiments for transcription.

The article at hand makes use of a recently developed multiple shooting for stochastic systems (MSS) objective function that treats the intervals between measurements separately. On each interval a linear noise approximation (LNA) is used in combination with a state updating scheme to handle non-observable components [14]. This separate treatment of the intervals means that the LNA is only needed on the relatively short time interval between measurements. The *FI*_*MSS*_ Fisher information is calculated based on this MSS objective function and pseudo data. The pseudo data is generated using the same approximation scheme as for the MSS objective function. The reason for the use of the Fisher information are its theoretical properties, the wide use in deterministic models and computational speed.

In contrast to [26] data from only one time course is sufficient for the new method of this article. The approach of [11] will be used as a benchmark for comparison. [11] calculated a Fisher information matrix without Monte Carlo simulations. This approach assumes that the observations are distributed with a multivariate normal distribution (MVN). The mean of this MVN is the ODE solution and the covariance matrix is calculated with the help of a LNA and it contains all inter-temporal covariances. This means that this approach applies the LNA to the whole systems horizon, in contrast to the MSS method which only applies to the intervals between measurements. This difference is of major importance: If the LNA holds over the whole systems horizon, it also holds on a shorter time scale. However, if it holds on shorter time scale, it does not necessarily hold over the whole systems horizon. The results section will illustrate the impact of this on experimental design methods.

Simple examples, as the Immigration-Death model, allow for an exact calculation of estimates and Fisher information matrices. This allows to compare the performance of the MSS method with an exact approach.

As the Fisher information is an asymptotic measure and calculated using the MSS objective function’s approximation, it is essential to investigate its performance. To address this matter, the Fisher information, which corresponds to the inverse of the covariance of the maximum likelihood estimator, is compared to a covariance matrix gained from different realizations of these estimator. These realizations are computed by simulating stochastic data sets and performing a parameter estimation on each of the data sets. The resulting estimates are used to calculate a covariance matrix, which is then compared to the Fisher information matrix. This comparison is based on optimality criteria, correlation structure or two-dimensional projection of the confidence intervals (which gives best visual interpretation and has also been suggested by [16]). As the Fisher information matrix is an asymptotic measure and calculated by approximations, an exact coverage of confidence intervals is unlikely. However, since the information content can be captured well enough to identify more informative designs, this is sufficient for experimental design. Note that the comparison of the Fisher information matrix to the covariance matrix from estimates is only done for investigating the performance. In general, when designing optimal experiments, only the evaluation of the Fisher information is needed and not the covariance from the estimates. The latter is only needed for evaluating the performance.

The methods section will recapitulate stochastic modeling, introduce the Fisher information matrix, and define the *FI*_*MSS*_ Fisher information matrix. The results section investigates the performance of the *FI*_*MSS*_ Fisher information matrix for three models: an Immigration-Death, a Lotka-Volterra and a Calcium oscillation model. Calcium oscillations play an important role in cell development and death as well as fertilization [29]. On top of that, the Calcium oscillation model is an especially challenging test case as it is highly nonlinear and shows a qualitatively different behavior in stochastic modeling than in deterministic modeling.

## Methods

### Stochastic Modeling of Biochemical Reactions

Computational modeling is a key technique for the analysis of complex systems in science. This subsection will introduce stochastic modeling and explain in which situations it is important.

Let *X* = (*X*_1_, …, *X*_*D*_) denote the *D* reactants in a system with *r* reactions in which *q*_*ij*_ denotes the number of educt molecules of species *X*_*i*_ for reaction *j* and *u*_*ij*_ the number of product molecules of species *X*_*i*_ for reaction *j*. Hence the system reads as

$$\begin{array}{c} q_{1j}\;X_{1}\;+\;q_{2j}\;X_{2}\;+\;\ldots \;+\;q_{Dj}\;X_{D}\;\;⟶\;\;u_{1j}\;X_{1}\;+\;u_{2j}\;X_{2}\;+\;\ldots \;+\;u_{Dj}\;X_{D},\\ \;\text{for}\;j=1,\ldots ,r \end{array}$$ (1)

The stoichiometric matrix *S* is a *D* × *r* dimensional matrix. Its entries *s*_*ij*_ = *u*_*ij*_−*q*_*ij*_ describe the net effect of reaction *j* to species *X*_*i*_. In terms of ODEs the system would read as

$$\frac{d}{dt}x\left(t;\theta ,x_{0}\right)\;=\;S\;v\left(x\left(t;\theta ,x_{0}\right),\theta \right),\;\text{with}\;x\left(0,\theta ,x_{0}\right)=x_{0}$$ (2)

and a rate law *v* = (*v*_1_, …, *v*_*r*_)^*T*^ describing the speed of the reactions, an initial concentration *x*_0_ and a parameter vector *θ*.

Stochastic modeling is important in systems with small numbers of molecules, where stochastic fluctuations can influence the system’s behavior [30]. It focuses on single particles and considers each reaction explicitly. Both order of reactions and waiting times are stochastic quantities depending on the system’s state and the rate laws. The chemical master equation (CME, Eq 3) describes the time evolution of the probability of the system to be in a state *ν*:

$$\begin{array}{cc} \frac{d}{dt}P_{\theta }\left(\nu ,t|\nu_{0},t_{0}\right)\; & =\;\sum_{j=1}^{r}\left({\tilde{v}}_{j}\left(\nu -s_{j},\theta \right)P_{\theta }\left(\nu -s_{j},t|x_{0},t_{0}\right)-{\tilde{v}}_{j}\left(\nu ,\theta \right)P_{\theta }\left(\nu ,t|\nu_{0},t_{0}\right)\right)\\ & P_{\theta }\left(\nu ,t_{0}|\nu_{0},t_{0}\right)=\left\{\begin{array}{cc} 1 & \nu =\nu_{0}\\ 0 & \text{else} \end{array}\right. \end{array}$$ (3)

with a vector *s*_*j*_ = (*s*_1*j*_, …, *s*_*Dj*_)^*T*^ and with a propensity $\tilde{v}$ that can be calculated from the rate law *v* and describes the speed of the reactions in terms of particle numbers. The rate constant *θ* needs to be defined in a volume independent way, otherwise apply transformations as in [31]. See [32] for detailed discussion on stochastic formulation for rate laws *v*_*j*_ of higher order reactions.

The Gillespie algorithm [2] is the method of choice to simulate stochastic time courses. It is an iterative algorithm simulating reaction event after reaction event using functions of random numbers to determine both time step and reaction. The resulting time course is then a discrete state continuous time Markov jump process, see also [33] for details. The stochastic time courses shown in this manuscript were simulated using the Gillespie implementation in COPASI [34].

Stochastic modeling can show system’s behavior that can not be seen with ODE modeling: Stochasticity can for example introduce bi-modality in genetic toggle switches, which have a stable steady state in ODE modeling [35]. The structure of Calcium oscillations may change qualitatively from ODE to stochastic modeling [36]. Furthermore, intrinsic stochasticity may provide information, e.g. regarding reactivity, which allow to solve identifiability problems [37]. This emphasizes the importance of stochastic modeling.

### The Fisher information Matrix

The Fisher information matrix measures the information provided by an experimental set-up for the estimation of the parameters. The theoretical result [19], which serves as the basis for the wide use [15, 16, 21, 22] of the Fisher information, states that the inverse of the Fisher information matrix *FI* is under certain regularity conditions [19] the asymptotic variance of a maximum likelihood estimator ${\hat{\vartheta }}^{\text{mle}}$ for a parameter *ϑ*:

$$\sqrt{n}\left({\hat{\vartheta }}_{\text{mle}}-\vartheta^{true}\right)\overset{\text{dist}}{\underset{n\to \infty }{\to }}MVN\left(0,FI{\left(T,\vartheta \right)}^{-1}\right),$$ (4)

where “dist” stands for convergence in distribution, MVN for multivariate normal distribution, *ϑ*^*true*^ for the true parameter value, and *T* = (*t*_0_, …, *t*_*n*_) for the set of time points, at which measurements are recorded. In case of structurally non identifiable parameters, the determinant of the Fisher information is zero and its inverse does not exist. Therefore, the relation (Eq 4) does only hold for scenarios, in which all parameters are identifiable.

A maximum likelihood estimator can be obtained by maximizing the likelihood function *L* over a parameter *ϑ*:

$${\hat{\vartheta }}_{\text{mle}}={\text{argmax}}_{\vartheta }\;L\left(O,\vartheta \right),$$ (5)

where the likelihood function L(O,ϑ) describes the probability to observe a data set O given a parameter *ϑ*. Intuitively spoken, the more sensitive the likelihood function is to changes in *ϑ* the more precise is an estimation of $\hat{\vartheta }$. The Fisher information matrix *FI* captures this sensitivity. Its components are defined as

$$FI{\left(T,\vartheta \right)}_{ij}\;=\;E_{O}\left[\frac{\partial }{\partial \vartheta_{i}}\;logL\left(O,\vartheta \right)\;\;\frac{\partial }{\partial \vartheta_{j}}\;logL\left(O,\vartheta \right)\right],\;i,j=1,\ldots ,n_{\vartheta }$$ (6)

where *n*_*ϑ*_ is the dimension of the parameter vector *ϑ*, and the expectation $E_{O}$ is calculated over all possible combinations of observations O. The Fisher information matrix is a symmetric matrix. The diagonal entries of its inverse describe the variance of the parameter estimates and the off-diagonal entries of its inverse the correlation of the parameter estimates.

#### Use of the Fisher information matrix

Due to the relation of Eq (4) the Fisher information can be used to calculate confidence intervals for each parameter or a multidimensional confidence area for all parameters. This includes information on the volume of the confidence ellipsoid and on the axis in the parameter space that can be identified with the lowest precision. Furthermore, the Fisher information can be used to obtain relative errors of the parameter estimates and extract correlation information between the parameters.

As the Fisher information only depends on the time points of the measurements but not on the actual outcome of an experiment, it is possible to calculate it before performing any experiment. This means that Fisher information matrices can be calculated for different experimental set-ups allowing the selection of the most informative design. This procedure is called experimental design. The goal is to obtain a parameter estimate that is as precise as possible, which means that its variance is as small as possible. As the Fisher information matrix is the inverse of the covariance matrix of the estimator, minimizing the variance means maximizing the Fisher information matrix. However, the task of maximizing a matrix is not well defined. To overcome this problem, several so called optimality criteria have been introduced [20], which map the Fisher information matrix to a real number.

#### Optimality criteria

Optimality criteria reflect measures of the parameter estimates’ confidence ellipsoids, which correspond to the Fisher information matrix. This article will use two optimality criteria:

- **D-criterion:** maps the Fisher information matrix to its determinant. The determinant corresponds to the volume of the confidence ellipsoid.
- **E-criterion** maps the Fisher information on its minimum eigenvalue. The minimum eigenvalue corresponds to the largest axis in the confidence ellipsoid.

While there are a lot more optimality criteria, see e.g. [20], the choice of the criterion depends on the focus of the experimenter. This article selects the D- and E-criterion as they give important information on the size and shape of the confidence ellispoid (volume and largest axis).

#### Computation of the Fisher information matrix for stochastic models

Eq (6) defines the Fisher information matrix. Its calculation is straightforward. However, the required computational time poses a big challenge, which makes the straightforward calculation infeasible for most realistic size models in systems biology. One reason is the expectation $E_{O}$ in Eq (6). Theoretically, this is a sum over all possible data sets O. If there are *n* measured time points, there are “number of points in state space” to the power of “*n*” summands. This is computational infeasible in most scenarios. Therefore, this article approximates this expectation with a mean over a subset of all data sets. This subset is created by generating *M* pseudo data sets $O^{\left(k\right)}(T),\;k=1,\ldots ,M$ using the Gillespie algorithm or with an alternative approach, as described in the “How to generate the pseudo data” subsection.

Another challenge is the evaluation of the likelihood function *L*. Analytical solutions are most commonly not available for models in systems biology. Approximations have to be accurate but still fast enough so that *M* can be chosen high enough to get a good approximation of the expectation. A MSS approximation for the log-likelihood function has been suggested by [13, 14]: $log\;L(O,\vartheta )\approx F_{MSS}(O,\vartheta )$. This *F*_MSS_ objective function will be introduced in the next subsection. (A more detailed explanation can be found in the appendix or the original articles [13, 14].)

The *FI*_*MSS*_ Fisher information matrix is based on the MSS objective function and reads as

$$\begin{array}{cc} FI_{\text{MSS}}{\left(T,\vartheta \right)}_{ij} & =\frac{1}{M}\sum_{k=1}^{M}\left(\frac{\partial }{\partial \vartheta_{i}}\;F_{MSS}\left(O^{\left(k\right)}\left(T\right),\vartheta \right)\;\frac{\partial }{\partial \vartheta_{j}}\;F_{MSS}\left(O^{\left(k\right)}\left(T\right),\vartheta \right)\right),\\ & i,j=1,\ldots ,n_{\vartheta } \end{array}$$ (7)

where the parameter *ϑ* contains the potential unobservable initial states *ν*_0_, the measurement noise covariance matrix Σ^meas^ and the kinetic parameters *θ*.

This *FI*_*MSS*_ Fisher information matrix can be used to assess the precision of the parameter estimates for an experimental set-up. It is possible to use pseudo data for a different number of system components to see the gain (or loss) in information by measuring more (or less) components. Furthermore, by varying the number of time points in *T*, the required number of time points for a predefined precision can be determined. This can be accomplished by calculating the optimal design for different numbers of measurements and then choose the smallest number of measurements for which the accuracy requirements are met. Additionally, it is possible to calculate an optimum experimental design by choosing the design T* that maximizes the value of an optimality criterion Φ:

$$\begin{array}{c} max_{T}\Phi \left(FI_{\text{MSS}}\left(T,\vartheta \right)\right). \end{array}$$ (8)

The power and accuracy of the *FI*_*MSS*_ Fisher information matrix will be demonstrated in the Results section on different test models.

### The MSS objective function

The MSS objective function has been suggested and shown to work for parameter estimation in stochastic models [12–14]. It assumes that observations are taken at discrete time points *t*_0_, *t*_1_, …, *t*_*n*_, where the system’s state is *ν* = (*ν*_0_, *ν*_1_, …, *ν*_*n*_). This system’s state is usually only imperfectly observable. This means that only some of the components can be observed and these observations are noisy: $O=(O_{0},\ldots ,O_{n})$. The main characteristics of the MSS objective function are:

- the time course data is split into intervals that are treated separately,
- unobserved states are handled by state updating,
- the distribution of a current state given its precursor is approximated with a normal distribution with mean and covariance gained by a linear noise approximation.

The MSS objective function will be derived briefly here, the details can be found in the S1 Text.

The observation based likelihood function L(O,ϑ) gives the probability to obtain the data O given a parameter *θ*:

$$L\left(O,\theta \right)\;=\;\prod_{i=1}^{n}\;P\left(O_{i};O_{i-1},\ldots ,O_{0},\theta \right),$$ (9)

where $P(O_{i};O_{i-1},\ldots ,O_{0},\theta )$ is the probability to observe $O_{i}$ given previous observations $O_{i-1},\ldots ,O_{0}$. This probability can be written as

$$P\left(O_{i};O_{i-1},\ldots ,O_{0},\theta \right)=\sum_{\nu_{i}\in \Omega_{i},\nu_{i-1}\in \Omega_{i-1}}P\left(O_{i};\nu_{i}\right)P\left(\nu_{i};\nu_{i-1},\theta \right)P\left(\nu_{i-1};O_{i-1},\ldots ,O_{0},\theta \right)$$ (10)

The first factor describes the measurement noise: $P(O_{i};\nu_{i})$ is the probability to measure $O_{i}$ if being in state *ν*_*i*_.

#### Transition probability

The second probability is the transition probability for a transition from to *ν*_*i*−1_ to *ν*_*i*_. Its distribution is generally unknown and [13, 14] suggest to approximate it with a normal distribution:

$$P\left(\nu_{i};\nu_{i-1},\theta \right)\approx p\left(\nu_{i};\nu_{i-1},\theta \right)=f\left(\nu_{i}|x\left(\Delta t;\theta ,\nu_{i-1}\right),\Sigma \left(\Delta t;\theta ,\nu_{i-1}\right)\right),$$ (11)

where *f*(*y*|*μ*, Σ) is the probability density function of a multivariate normal distribution with mean *μ* and covariance Σ which is calculated by a linear noise approximation

$$\begin{array}{cc} \frac{d}{dt}\Sigma \left(t;\theta \right) & =J\left(x,\theta \right)\Sigma \left(t;\theta \right)+\Sigma \left(t;\theta \right)J{\left(x,\theta \right)}^{\prime }+\Omega^{-1}D\left(x,\theta \right),\;\Sigma \left(0,\theta \right)=0_{D\times D}\\ \text{with}\;J(x,\theta ) & =S\frac{d}{dx}v\left(x,\theta \right)\;\;\text{and}\;D=(D_{ij})\;\text{with}\;\\ D_{ij}\left(x,\theta \right) & =\sum_{k=1}^{R}S_{ik}S_{jk}v_{k}\left(x,\theta \right)\; \end{array}$$ (12)

with *x* = *x*(Δ*t*; *θ*, *ν*_*i*−1_), the solution of Eq (2), a volume Ω and’ denoting the transpose of a matrix.

As the Gaussian distribution has a continuous support, the probability for $P(O_{i};O_{i-1},\ldots ,O_{0},\theta )$ in Eq (10) is calculated with an integral instead of the sum:

$$\begin{array}{cc} P(O_{i};O_{i-1},\ldots ,O_{0},\theta )\approx & \\ \int_{\nu_{i}\in \Lambda_{i},\nu_{i-1}\in \Lambda_{i-1}} & P\left(O_{i};\nu_{i}\right)p\left(\nu_{i};\nu_{i-1},\theta \right)P\left(\nu_{i-1};O_{i-1},\ldots ,O_{0},\theta \right)d\nu_{i}d\nu_{i-1} \end{array}$$ (13)

where Λ_*i*_ stands for the state space at time point *t*_*i*_. In many cases the state space will be constant over time, hence Λ = Λ_1_ = Λ_2_, … = Λ_*n*_.

#### State estimation

The third probability $P(\nu_{i-1};O_{i-1},\ldots ,O_{0},\theta )$ is the probability to be in a state *ν*_*i*−1_ given the observations $O_{i-1},\ldots ,O_{0}$. [14] suggests to use a state updating procedure instead of the full probability distribution $P(\nu_{i-1};O_{i-1},\ldots ,O_{0},\theta )$ to estimate the state *ν*_*i*−1_ at time point *t*_*i*−1_: Given a state estimate ${\hat{\nu }}_{i-1}$ at time *t*_*i*−1_, the probability to see the observation $O_{i}$ at time *t*_*i*_ is the product of the probability to move from state ${\hat{\nu }}_{i-1}$ to a state *ν*_*i*_ and the probability to see $O_{i}$ if the state is *ν*_*i*_. A state estimate ${\hat{\nu }}_{i}$ can be defined as the state that leads to the highest probability to observe $O_{i}$:

$${\hat{\nu }}_{i}={\text{argmax}}_{\nu_{i}}\left(f\left(\nu_{i}|x\left(\Delta t;\theta ,{\hat{\nu }}_{i-1}\right),\Sigma \left(\Delta t;\theta ,{\hat{\nu }}_{i-1}\right)\right)\cdot f\left(O_{i}|\nu_{i}^{\text{obs}},\Sigma^{\text{meas}}\right)\right)$$ (14)

for *i* = 1, …, *n* − 1 and *x* as in Eq (2) and Σ as in Eq (12). The initial state ${\hat{\nu }}_{0}$ is included into the optimization vector.

#### The MSS objective function

Taking the logarithm of Eq (9), inserting the approximation of Eq (11) and assuming a Gaussian measurement error, leads to the MSS objective function:

$$\begin{array}{c} F_{\text{MSS}}\left(O,\left(\theta ,\nu_{0},\Sigma^{\text{meas}}\right)\right)=\sum_{i=1}^{n}\text{log}\int_{\nu_{i}\in \Lambda }\;\;f\left(O_{i}|\nu_{i},\Sigma^{\text{meas}}\right)\\ \;\;f\left(\nu_{i}|x\left(\Delta_{i};\theta ,{\hat{\nu }}_{i-1}\right),\Sigma \left(\Delta_{i};\theta \right)\right)\;\;d\nu_{i}. \end{array}$$ (15)

The parameter *ϑ* = (*θ*, *ν*_0_, Σ^meas^) is composed of the kinetic parameters *θ*, the initial state *ν*_0_ and the measurement noise covariance matrix *Σ*^meas^.

### How to generate the pseudo data?

Eq (7) approximates the expectation $E_{O}$ over all possible data sets by the mean over a subset of all data sets. This subset is created by simulating pseudo data sets. Therefore, one needs a way to generate these pseudo data sets. The following scheme has been indicated in [13]. The distribution of *ν*_*i*_ at time point *t*_*i*_ given the knowledge of previous state *ν*_*i*−1_ is approximated with *ν*_*i*_|*ν*_*i*−1_ ∼ *N*(*x*(Δ*t*; *θ*, *ν*_*i*−1_), Σ(Δ*t*; *θ*, *ν*_*i*−1_)). This can be used to generate pseudo data trajectories *ν*(*T*) by iteratively drawing random numbers according to this distribution. The argument “(T)” is hereby used to denote the time points *t*_0_, …, *t*_*n*_ of the pseudo data. Given a state *ν*_*i*−1_, the next state *ν*_*i*_ and the next pseudo observation $O_{i}(T)$ are calculated as

$$\begin{array}{c} \nu_{i}\left(T\right)=\;\text{Round}\left(x\left(\Delta_{i};\theta ,\nu_{i-1}\right)\;+\;u_{i}\;A_{i}\right)\\ O_{i}\left(T\right)=\nu_{i}\;+\;{\tilde{u}}_{i}\;B_{i} \end{array}$$ (16)

with $\Sigma \left(\Delta_{i};\theta \right)=A_{i}^{\prime }A_{i}$, $\Sigma^{\text{meas}}=B_{i}^{\prime }B_{i}$ and random numbers *u*_*i*_ ∼ *N*(0_*D*_, 1_*D*×*D*_) and ${\tilde{u}}_{i}\;∼N\left(0_{\text{obs}},1_{\text{obs}\times \text{obs}}\right)$ with a *D* × *D* matrix 1_*D*×*D*_ with diagonal entries 1 and a vector 0_*D*_ of length *D* with zero entries. The length of the vector of observables is denoted by “obs”.

To ensure consistency in the generation of pseudo data and the evaluation of the MSS Fisher information matrix this scheme was applied (instead of e.g. a standard Gillespie algorithm). Furthermore, the suggested pseudo data generation scheme has the advantage that it allows for a continuous dependency between parameters and system’s state (by leaving out the “Round” operation)operation), which is not possible using a standard Gillespie algorithm.

### A benchmark approach for comparison

The method from [11] is used as a benchmark. It is also based on a LNA, however, it is assumed that the LNA holds for the whole time horizon, namely from *t*_0_ until *t*_*n*_. [11] calculates inter-temporal covariances by

$$\begin{array}{c} cov\left(\nu \left(s\right),\nu \left(t\right)\right)=\Sigma \left(s;\theta \right)\Phi {\left(s,t\right)}^{\prime }\;\text{for}\;t\geq s \end{array}$$ (17)

The fundamental matrix Φ of the non-autonomous system is calculated by

$$\begin{array}{c} \frac{d\Phi (s,t)}{dt}=J\left(x,\theta \right)\Phi \left(s,t\right),\;\text{with}\;\Phi \left(s,s\right)=I \end{array}$$ (18)

with an identity matrix *I*. The observation sequence *ν* = (*ν*_0_, …, *ν*_*n*_) is then considered to be multivariate normal distributed

$$\begin{array}{c} \nu ∼\text{MVN}(\mu \left(T,\theta \right),\Sigma_{B}\left(T,\theta \right)) \end{array}$$ (19)

with

$$\begin{array}{c} \mu \left(T,\theta \right)=\left(x\left(t_{0};\theta ,\nu_{0}\right),\ldots ,x\left(t_{n}-t_{0};\theta ,\nu_{0}\right)\right) \end{array}$$ (20)

and a symmetric matrix

$$\Sigma_{B}{\left(T,\theta \right)}^{i,j}=\left\{\begin{array}{cc} \Sigma (t_{i};\theta ), & i=j\\ \Sigma \left(t_{i};\theta \right)\Phi {\left(t_{i},t_{j}\right)}^{\prime }, & i<j \end{array}\right.$$ (21)

A parameter estimate can be calculated by maximizing the probability of the MVN distribution in Eq (19) to observe *ν* over the parameter *θ*. A Fisher information matrix *FI*_*Bench*_ can be calculated using [38] with $FI_{Bench}(T,\theta )={\left(FI_{Bench}^{j,k}(T,\theta )\right)}_{j,k=1,\ldots ,n_{\theta }}$ and

$$\begin{array}{lll} FI_{Bench}^{j,k}(T,\theta ) & = & \frac{\partial \mu {\left(T,\theta \right)}^{\prime }}{\partial \theta_{j}}\Sigma_{B}{\left(T,\theta \right)}^{-1}\frac{\partial \mu (T,\theta )}{\partial \theta_{k}}\\ & + & \frac{1}{2}trace(\Sigma_{B}{\left(T,\theta \right)}^{-1}\frac{\partial \Sigma_{B}(T,\theta )}{\partial \theta_{j}}\Sigma_{B}{\left(T,\theta \right)}^{-1}\frac{\partial \Sigma_{B}(T,\theta )}{\partial \theta_{k}}) \end{array}$$ (22)

Note that the first summand needs to contain the inverse of *Σ*_*B*_ as in [38].

## Results

### Design of the simulation study

To ensure the validity of the *FI*_*MSS*_ Fisher information approximation the accuracy thereof has to be evaluated. The Fisher information in itself is an asymptotic measure and the *FI*_*MSS*_ Fisher information matrix for stochastic models is particularly approximative using the LNA approximation from the MSS objective function.

Two quantities are calculated for the accuracy evaluation: on one hand the new *FI*_*MSS*_ Fisher information matrix for stochastic models. On the other hand, *N*_*sim*_ stochastic time courses are simulated with the Gillespie algorithm [2], and for each of them a parameter estimation is performed with the MSS objective function [14] (see the “Settings for the parameter estimation” section in the S1 Text). This results in *N*_*sim*_ parameter estimates. A covariance matrix is calculated from these *N*_*sim*_ parameter estimates and denoted by *Cov*. Its inverse *Cov*^−1^ is denoted by *FI*_*emp*_. As *FI*_*MSS*_ corresponds asymptotically to the inverse of the covariance matrix, Eq (4), *FI*_*MSS*_ can be compared to *FI*_*emp*_.

Different measures are employed to compare the two matrices *FI*_*MSS*_ and *FI*_*emp*_:

- a comparison based on optimality criteria such as D- and E-criterion (introduced in the “Optimality criteria” subsection),
- The *i*−*th* diagonal entry of the inverse of the Fisher information matrix corresponds to the variance of *i*−*th* component of the parameter estimate ${\hat{\theta }}_{i}$. Therefore, the average relative squared error (ARSE)
  $$ARSE\left(\theta_{i}\right)\;=100{\left(\frac{1}{N_{sim}}\sum_{i=1}^{N_{sim}}\frac{{\left({\hat{\theta }}_{i}-\theta_{i}^{\left(0\right)}\right)}^{2}}{{\theta_{i}^{\left(0\right)}}^{2}}\right)}^{1/2}$$ (23)
  can be compared to $100\sqrt{{\left(FI_{MSS}\right)}_{ii}}/\theta_{i}^{\left(0\right)}$ with known true parameter *θ*^(0)^. The average ARSE over all parameter components is sometimes named “A-criterion”,
- a visualization of the 2-d projections of the confidence ellipsoids and a comparison of its shape to the cloud of points of the parameter estimates as also suggested by [16],
- a comparison of the parameter correlations.

### Immigration-Death model

The first example is an Immigration-Death model:

$$⌀\overset{\theta_{1}}{\to }X$$ (24)

$$X\overset{\theta_{2}x}{\to }\;⌀$$ (25)

where *X* is the substance and *θ*_1_, *θ*_2_ are parameters. The representation in ODEs reads as

$$\frac{dx}{dt}\;=\;\theta_{1}\;-\theta_{2}x,\;\;x\left(0\right)=x_{0}.$$ (26)

This model can be used as a simple model of constitutive gene expression [39], where *X* is the amount of transcript, *θ*_1_ the transcription rate and *θ*_2_ the mRNA degradation rate.

The Immigration-Death model has a very interesting property: using an ODE model and steady state data, only the quotient of the parameters *θ*_1_ and *θ*_2_ is identifiable but not their absolute values. However, using a stochastic model, the information encoded in the intrinsic fluctuations allows the identification of both parameters [40]. This property has to be reflected in the Fisher information matrix. Therefore, this relatively simple model is a valuable part of the test set for the *FI*_*MSS*_ Fisher information matrix.

Various scenarios are taken into account with each 100 observations and different inter-sample distances ranging from Δ*t* = 0.1 to Δ*t* = 15. *x*_0_ = 10 is chosen as initial value with *θ*_1_ = 1 and *θ*_2_ = 0.1, as this configuration leads to a steady state. The *FI*_*MSS*_ Fisher information is calculated for all inter-sample distances according to Eq 7 with *M* = 1000 pseudo data sets. The pseudo data sets are generated as explained in “How to generate the pseudo data” subsection.

**Fig 2:**
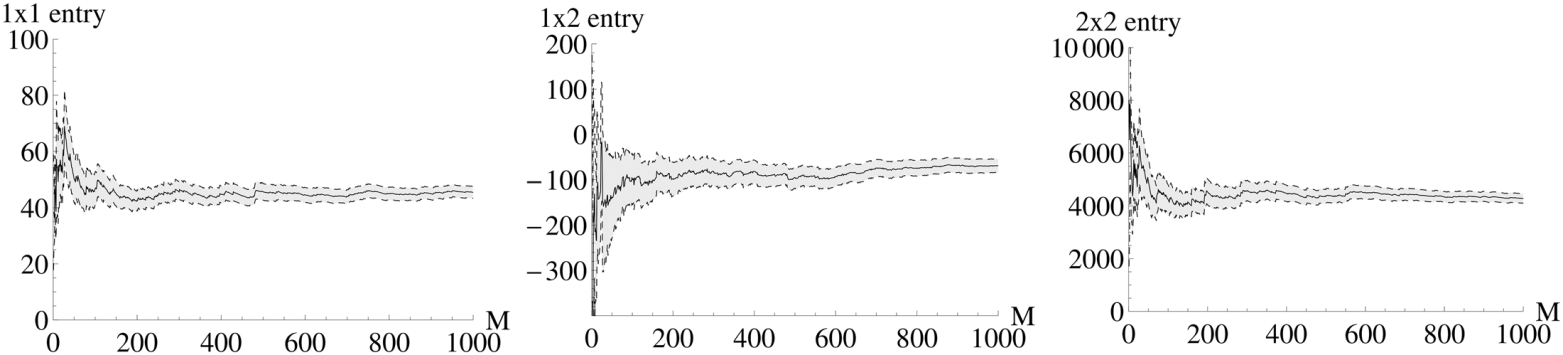
Dependence of the accuracy of the *FI*_*MSS*_ entries on the number of pseudo data sets. Each panel shows one entry of *FI*_*MSS*_. Note that the 2 × 1 entry is identical to the 1 × 2 entry due to the symmetry of the Fisher information matrix. The x-axis shows the number of pseudo data sets *M* used for calculating the sample mean (shown as solid line) of Eq 7. Gray color indicates the area from sample mean plus / minus one standard deviation. As the width of the gray are is decreasing, the accuracy increases and an acceptable accuracy is reached at values around M = 200.

The *FI*_*MSS*_ Fisher information matrix is calculated based on a finite pseudo data set (*M* = 1000) where each entry of *FI*_*MSS*_ represents a sample mean. Therefore, the first question is how the accuracy of the entries of *FI*_*MSS*_ depends on the number of pseudo data sets *M*. Fig 2 shows how the entries of *FI*_*MSS*_ converge with increasing *M*. One can see that already a number of *M* as low as 200 leads to an acceptable accuracy for *FI*_*MSS*_.

As the Fisher information is an asymptotic measure and the *FI*_*MSS*_ Fisher information matrix is based on the MSS objective function, the next step is to investigate the accuracy of *FI*_*MSS*_ compared to *FI*_*emp*_. *FI*_*emp*_ is the inverse of the covariance of estimates gained from simulated data. For each experimental design *N*_*sim*_ = 1000 data sets are simulated and 1000 parameter estimates calculated. The experimental designs vary in their inter-sample distance and contain, for better comparison, 100 observations each. Whenever an estimate ${\hat{\theta }}_{1}$ was greater than 3, it was counted as non-converging. This happened 28 times for Δ*t* = 15, 7 times for Δ*t* = 12.5, and 3 times for Δ*t* = 10. Convergence was achieved for each data set for all remaining inter-sample distances.

**Fig 3:**
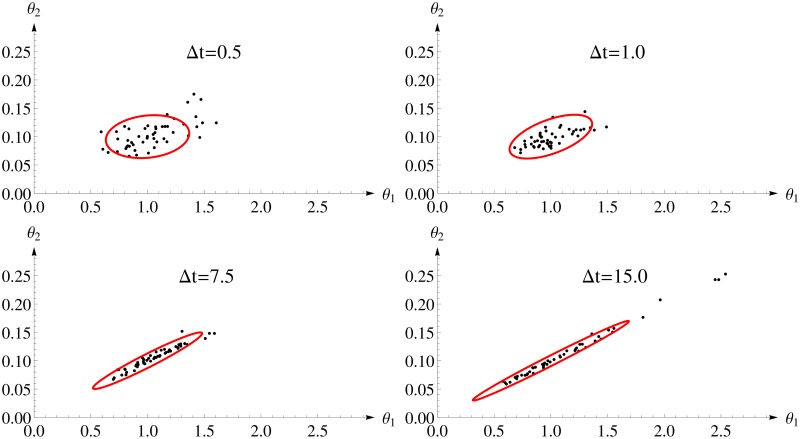
Parameter estimates and two dimensional confidence ellipsoids from the *FI*_*MSS*_ Fisher information for different design of the Immigration-Death model. Each panel considers one experimental design with varying inter-sample distances Δ*t* and 100 observations. The confidence ellipsoid (red) of the *FI*_*MSS*_ is able to represent the shape of the distribution of the estimates.

Fig 3 shows the 95%-confidence ellipsoid calculated for *FI*_*MSS*_ and the first 50 estimates. One can see a distinct change in the shape of the confidence ellipsoid from almost round for small inter-sample distance to rather stretched for larger inter-sample distances. The reason is that higher inter-sample distances allow for a better determination of the steady state and, therefore, for the quotient of the parameters. But, these designs collect less information on the intrinsic fluctuations as the inter-sample distance approaches the auto-correlation time and, therefore, the absolute value can be identified with lower precision only. The *FI*_*MSS*_ Fisher information covers this change very well.

**Fig 4:**
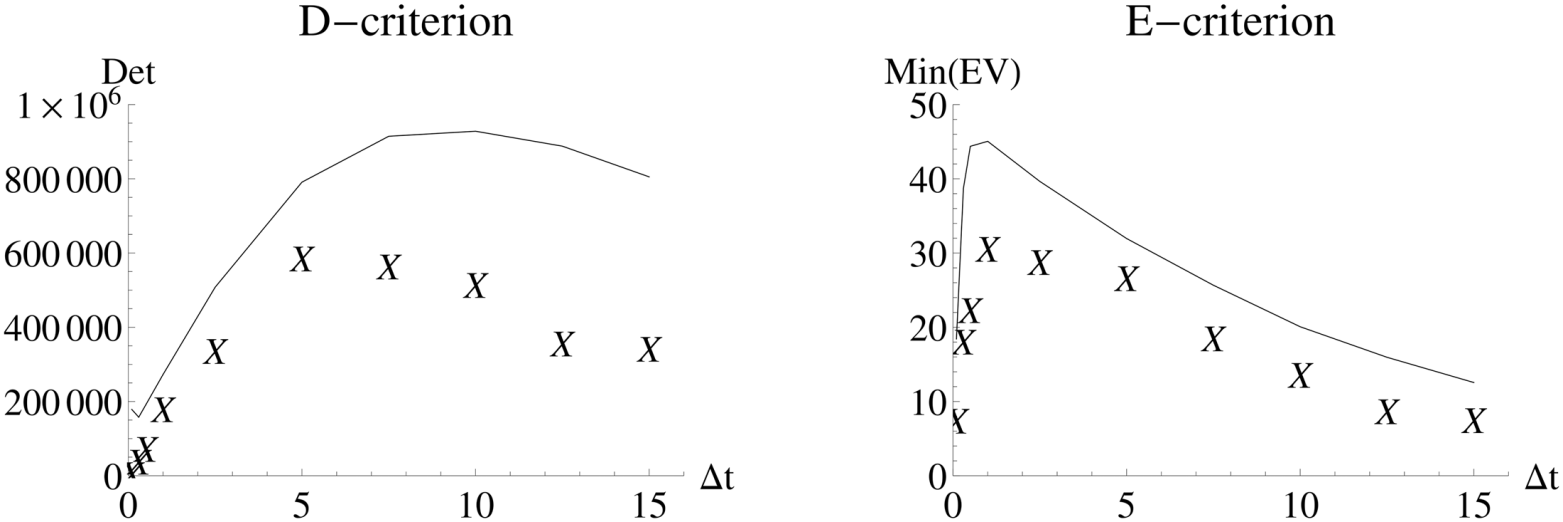
D-criterion and E-criterion for different experimental designs for the Immigration-Death model. *FI*_*MSS*_ Fisher information and *FI*_*emp*_ are calculated for different inter-sample distances. The solid line is an interpolation of the values of *FI*_*MSS*_ and the “X” denote the values of *FI*_*emp*_.

Fig 4 shows the evaluation of the performance of the *FI*_*MSS*_ in comparison to *FI*_*emp*_ based on the D- and E-criteria. While the *FI*_*MSS*_ covers the dynamics well, there is a bias towards overestimating the information content due to its asymptotic nature. However, it is still accurate enough to allow for experimental design, namely choosing an inter-sample distance that leads to an E-optimal design (Δ*t* = 1) or an D-optimal design which needs a larger inter-sample distance of 5 to 10. While the *FI*_*MSS*_ would suggest the higher value, *FI*_*emp*_ would suggest the lower value. This difference is again due to the non-asymptotic scenario and the approximation in the MSS objective function.

The D-criterion values of *FI*_*MSS*_ between Δ*t* = 5 and Δ*t* = 10 are relatively similar. The same holds for the values of *FI*_*emp*_. This demonstrates a good performance of the MSS Fisher information but also that choosing a good design is fairly robust towards the inter-sample distance. This is important information because it means that deviation of the experimental schedule by 5 time units will not strongly influence the quality of the outcome. However, looking at the E-criterion, a deviation of 5 time units from the optimal inter-sampling schedule will have a stronger impact as the values of the E-criterion for e.g. Δ*t* = 0.3 are much smaller than the optimal values at Δ*t* = 1.

**Fig 5:**
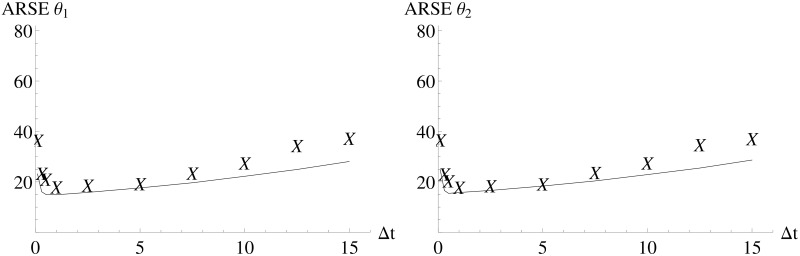
ARSE for different experimental designs for the Immigration-Death model. *FI*_*MSS*_ and *FI*_*emp*_ are calculated for different inter-sample distances. The solid line is an interpolation of the values of *FI*_*MSS*_ and the “X” denote the values of *FI*_*emp*_.

Additionally, the performance of *FI*_*MSS*_ is also evaluated based on the ARSE (Fig 5). While there is a slight underestimation of the ARSE, again due to the asymptotic nature of the Fisher information, the *FI*_*MSS*_ capture the dependency on the inter-sample distance well.

The Fisher information matrix can also be used to extract information on the correlation between the parameters *θ*_1_ and *θ*_2_. Table 1 summarizes the correlation based on the inverse of *FI*_*MSS*_ denoted by *Corr*(*FI*_*MSS*_) and the correlation of the parameter estimates from the simulated data denoted by *Corr*_*emp*_ for four designs.

**Table 1:** Different experimental designs and their parameter correlation.

Δ*t*	*Corr*(*FI*_*MSS*_)	*Corr*_*emp*_
0.5	0.16	0.52
1.0	0.58	0.68
7.5	0.97	0.97
15.0	0.99	0.99

The first column shows the correlation of *θ*_1_ and *θ*_2_ based on *FI*_*MSS*_ and the second column based on the estimates from simulated data for experimental designs with different inter-sample distances.

The computational time for an evaluation of the new *FI*_*MSS*_ Fisher information matrix with *M* = 1000 takes less than 1 minute on an Intel Core i7-3770 CPU with 16GB RAM using one kernel.

#### Comparison with the benchmark approach

The approach of [11] is applied to the Immigration-Death model as a benchmark. The Fisher information matrix *FI*_*Bench*_ is calculated as well as 1000 parameter estimates for the same data set used for the MSS method. *FI*_*emp*,*Bench*_ is calculated from these estimates. As for the MSS method, estimates with an *θ*_1_ > 3 are counted as non-converging. Out of the 1000 estimates 2 were non-converging for Δ*t* = 10, 9 for Δ*t* = 12.5 and 33 for Δ*t* = 15.

**Fig 6:**
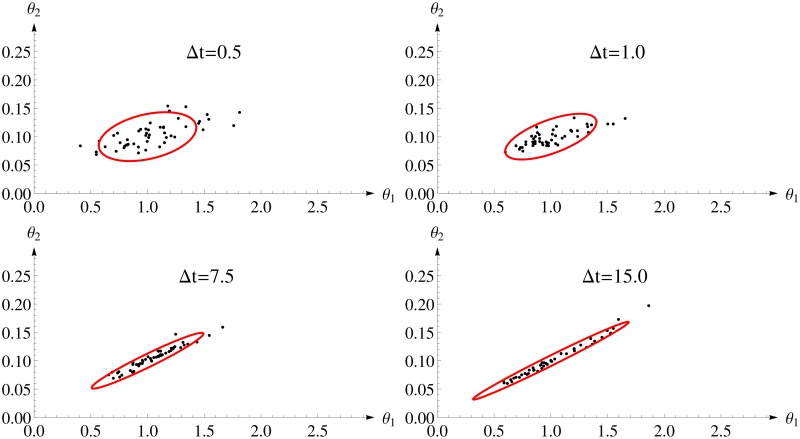
Parameter estimates and two dimensional confidence ellipsoids of the benchmark method for different design of the Immigration-Death model. Each panel considers one experimental design with varying inter-sample distances Δ*t* and 100 observations. The confidence ellipsoid (red) of the *FI*_*Bench*_ corresponds well with the location of the parameter estimates.

Fig 6 shows the first 50 estimates and the 2-dimensional confidence ellipsoid of *FI*_*Bench*_. The accuracy of the parameter estimates is comparable to the MSS method (Fig 3) as the estimates have a similar distance to the true value (1, 0.1). The Fisher information *FI*_*Bench*_ leads to a 2-dimensional confidence interval that captures the location of the estimates similarly well as *FI*_*MSS*_.

**Fig 7:**
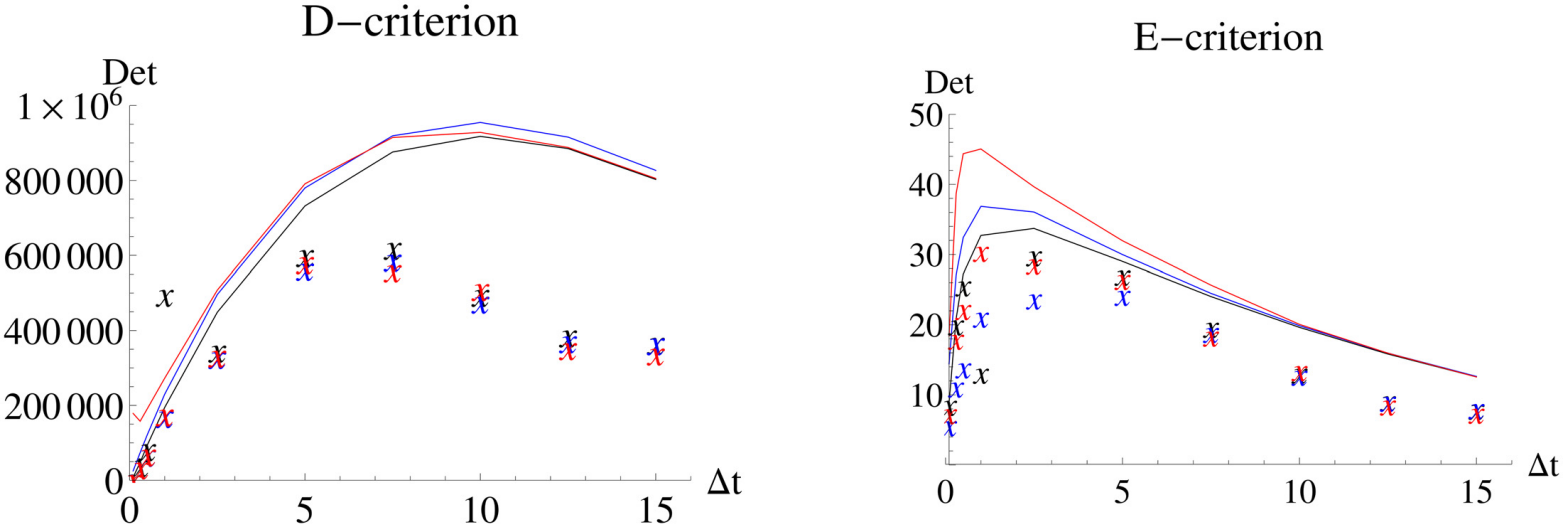
D-criterion and E-criterion for different experimental designs for the Immigration-Death model for all three methods (MSS, benchmark and exact). *FI*_⋅_ Fisher information and *FI*_*emp*,⋅_ are calculated for different inter-sample distances. The solid line is an interpolation of the values of *FI*_⋅_ and the “x” denote the values of *FI*_*emp*,⋅_. Red color corresponds to the MSS method, blue to the benchmark and black to the exact method. Symbols partially overlapping.

**Fig 8:**
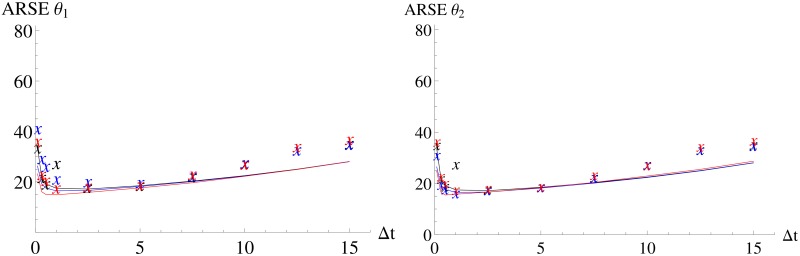
ARSE for all three method for different experimental designs for the Immigration-Death model. *FI*_⋅_ and *FI*_*emp*,⋅_ are calculated for different inter-sample distances. The solid line is an interpolation of the values of *FI*_*ex*_ and the “X” denote the values of *FI*_*emp*,⋅_. Red color corresponds to the MSS method, blue to the benchmark and black to the exact method. Symbols partially overlapping.

Figs 7 and 8 and Table 2 confirm that the benchmark approach is able to capture the changes in the volume (D-criterion), the longest axis (E-criterion), the ARSEs as well as the correlation. Therefore, both the MSS and the benchmark are well suited for parameter estimation and a calculation of a Fisher information matrix for this model.

**Table 2:** Different experimental designs and their parameter correlation for the benchmark method.

Δ*t*	*Corr*(*FI*_*Bench*_)	*Corr*_*Bench*,*emp*_
0.5	0.37	0.64
1.0	0.64	0.75
7.5	0.97	0.97
15.0	0.99	0.99

The first column shows the correlation of *θ*_1_ and *θ*_2_ based on *FI*_*Bench*_ and the second column based on the estimates from simulated data for experimental designs with different inter-sample distances.

#### Comparison with an exact method

The Immigration-Death example with the above mentioned parametrization is small enough to apply a state truncation and an exact method (ex) for the parameter estimation as well as the calculation of the Fisher information matrix. This approach is based on an analytical calculation of the transition probabilities *P*(*ν*_*i*_; *ν*_*i*−1_, *θ*) as described in [41]:

Pex(νi,Δt;νi-1,θ)=1k!∑j=0kkjθ1θ2je-θ1θ2(1-e-θ2Δt) (27)

$$\;\;\;\;\;\;\;\left.\;{\left(1-e^{-\theta_{2}\Delta t}\right)}^{\nu_{0}-k-2j}\;{\left(e^{-\theta_{2}\Delta t}\right)}^{k-j}\;\prod_{i=0}^{k-j-1}\left(\nu_{0}-i\right)\right).$$ (28)

The likelihood function is defined as the product of these transition probabilities:

$$L_{ex}=\prod_{i=1}^{n}P_{ex}\left(\nu_{i},t_{i}-t_{i-1};\nu_{i-1},\theta \right)$$ (29)

A Fisher information matrix can be calculated as

$$\begin{array}{cc} FI_{ex}{\left(T,\theta \right)}_{jk} & =\sum_{i=1}^{n}\sum_{\nu_{i},\nu_{i-1}}\frac{\partial }{\partial \theta_{j}}\;logP_{ex}\left(\nu_{i},t_{i}-t_{i-1};\nu_{i-1},\theta \right)\\ & \frac{\partial }{\partial \theta_{k}}\;logP_{ex}\left(\nu_{i},t_{i}-t_{i-1};\nu_{i-1},\theta \right)\\ & P_{ex}\left(\nu_{i},t_{i}-t_{i-1};\nu_{i-1},\theta \right)\;\;\;P_{ex}\left(\nu_{i-1},t_{i-1}-t_{0};\nu_{0},\theta \right) \end{array}$$ (30)

See S1 Text for details. The sum ∑_*ν*_*i*_,*ν*_*i*−1__ is infinite. However, it is replaced by ∑_*ν*_*i*_,*ν*_*i*−1_ ≤ 30_ as the probability to reach a higher number is very small even for large time scales: $1-\sum_{\nu_{i}=0}^{30}P_{ex}\left(\nu_{i},1500;0,\left(1,0.1\right)\right)=7.98\times 10^{-8}$, 1500 being the longest observation duration used in this simulation study.

Next, the exact approach is applied to the Immigration-Death model. The Fisher information matrix *FI*_*ex*_ is calculated as well as 1000 parameter estimates for the same data set used for the MSS method and the method of [11]. *FI*_*emp*,*ex*_ is calculated from these estimates. As with the MSS method, estimates with an *θ*_1_ > 3 are counted as non-converging and this happened 2 times for Δ*t* = 1, 2 times for Δ*t* = 10, 9 times for Δ*t* = 12.5 and 32 times for Δ*t* = 15 out of the 1000 estimates for each scenario.

**Fig 9:**
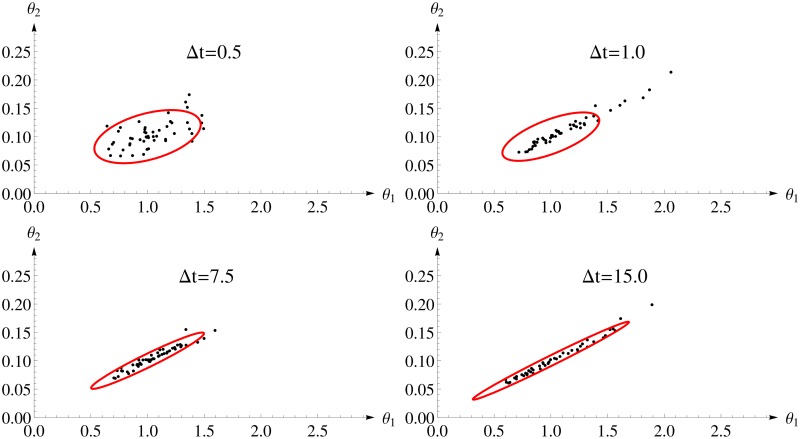
Parameter estimates and two dimensional confidence ellipsoids for the exact method for different design of the Immigration-Death model. Each panel considers one experimental design with varying inter-sample distances Δ*t* and 100 observations. The confidence ellipsoid (red) of the *FI*_*ex*_ is similar than the confidence ellipsoid of the *FI*_*MSS*_ in Fig 3.

Fig 9 shows the first 50 estimates and the 2-dimensional confidence ellipsoid of *FI*_*ex*_. The accuracy of the parameter estimates is comparable to the MSS method in Fig 3 (showing that the approximation does not lead to a loss in accuracy). The 2-dimensional confidence intervals describe the change in the cloud of estimates from rather round to rather stretched well and, more important, the 2-dimensional confidence intervals are similar to those calculated with *FI*_*MSS*_ in Fig 3.

The observation for the D-criterion, the E-criterion and the ARSEs is similar: *FI*_*ex*_ and *FI*_*emp*,*ex*_ correspond very well. Again, more important, the results from *FI*_*MSS*_ and *FI*_*ex*_ are very similar (see Figs 7 and 8) which shows that the MSS method is able to calculate accurate Fisher information matrices. The same holds for capturing the correlation (MSS results in Table 1, exact method in Table 3).

**Table 3:** Different experimental designs and their parameter correlation for the exact method.

Δ*t*	*Corr*(*FI*_*Bench*_)	*Corr*_*Bench*,*emp*_
0.5	0.45	0.45
1.0	0.67	0.98
7.5	0.97	0.97
15.0	0.99	0.99

The first column shows the correlation of *θ*_1_ and *θ*_2_ based on *FI*_*ex*_ and the second column based on the estimates from simulated data for experimental designs with different inter-sample distances.

Fig 7 also shows that all three approaches overestimate the D- and E- criterion values. To ensure that this does not depend on the specific data set, the exact method was also investigated using different data sets (S6 Fig) showing consistent results. The reason for the overestimation seems to be that the Fisher information is an asymptotic measure and this causes difficulties for longer step-sizes in the Immigration-Death model.

### Lotka-Volterra model

The next example is a Lotka-Volterra model, which shows oscillatory behavior. The model consists of three reactions:

$$\begin{array}{ccc} Y^{\left(1\right)} & \overset{\theta_{1}\;Y^{\left(1\right)}}{\to } & 2\;Y^{\left(1\right)}\\ Y^{\left(1\right)}+Y^{\left(2\right)} & \overset{\theta_{2}\;Y^{\left(1\right)}\;Y^{\left(2\right)}}{\to } & 2\;Y^{\left(2\right)}\\ Y^{\left(2\right)}\;\;\; & \overset{\theta_{3}\;Y^{\left(2\right)}}{\to } & \;\;\;⌀ \end{array}$$ (31)

where *Y*^(1)^ and *Y*^(2)^ denote prey and predator, respectively, and *θ*_1_, *θ*_2_, *θ*_3_ are parameters. The first reaction of Eq (30) is the prey reproduction, the second the predator reproduction, and the third is the predator death. In terms of ODEs this system reads as

$$\begin{array}{cc} \frac{d}{dt}y^{\left(1\right)}\left(t\right)\; & =\;\theta_{1}y^{\left(1\right)}\left(t\right)-\theta_{2}y^{\left(1\right)}\left(t\right)y^{\left(2\right)}\left(t\right)\\ \frac{d}{dt}y^{\left(2\right)}\left(t\right)\; & =\;\theta_{2}y^{\left(1\right)}\left(t\right)y^{\left(2\right)}\left(t\right)-\theta_{3}y^{\left(2\right)}\left(t\right). \end{array}$$ (32)

The true parameter is set to *θ*^(0)^ = (0.5, 0.0025, 0.3) and the initial values to *Y* = (71, 79) as in [9].

As in the Immigration-Death model, a good approximation is already obtained using as few as *M* = 200 pseudo data sets for the evaluation of the *FI*_*MSS*_ Fisher information matrix (see S1 and S2 Figs).

**Fig 10:**
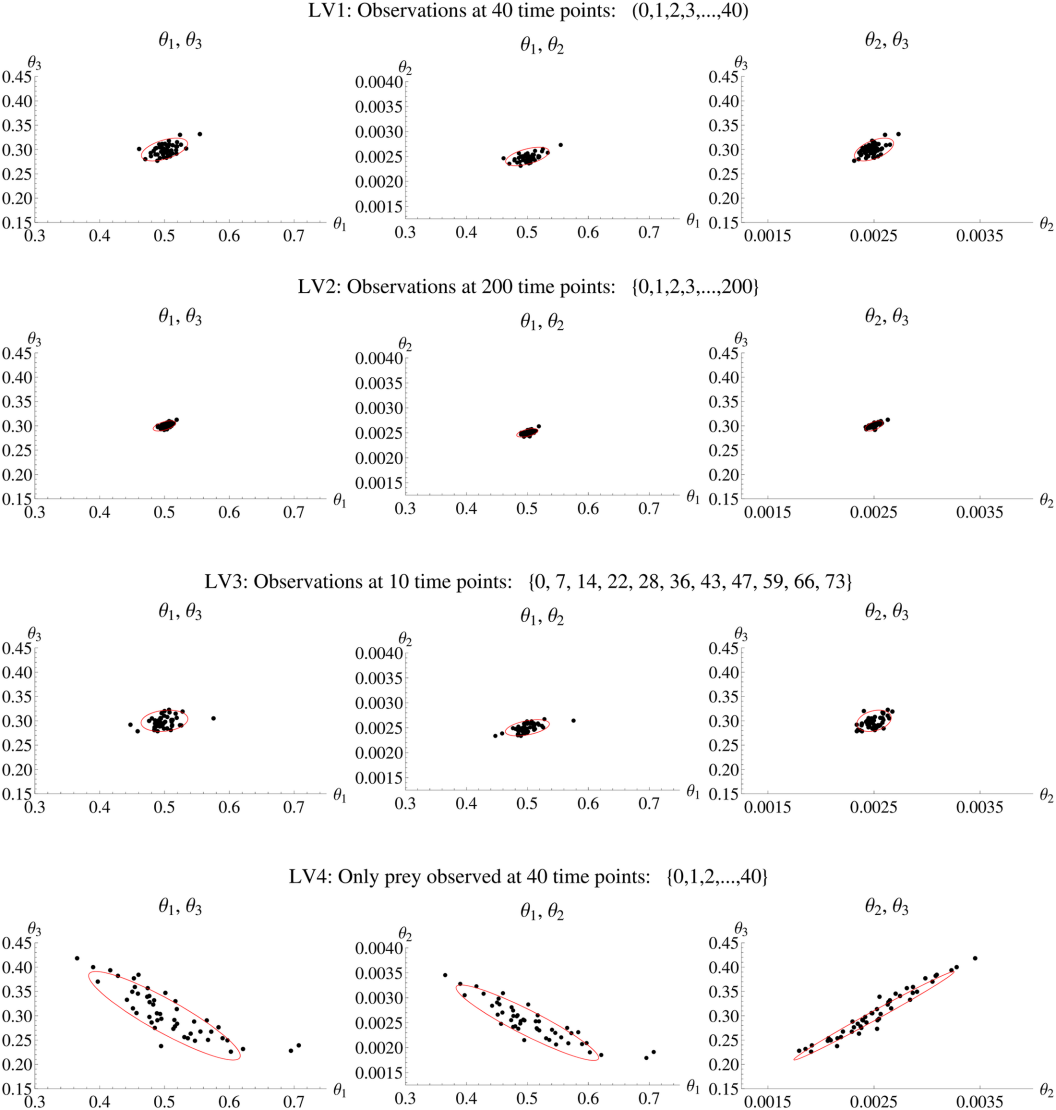
Parameter estimates and confidence ellipsoid from *FI*_*MSS*_ Fisher information for Lotka-Volterra model. Each row shows one of the scenarios LV1 to LV4. In each row the three panels show one two dimensional projection of the three dimensional parameter space. In each panel the black dots are the estimates from simulated data and the confidence ellipsoid from *FI*_*MSS*_ is marked red.

Four different experimental designs of the Lotka-Volterra model are compared to analyze the impact on the amount of information gained in the experiment. The first experimental design LV1 consists of 40 equidistant observations of both prey and predator with an inter-sample distance of 1. As the parameter space is three-dimensional, Fig 10(LV1) shows the three two-dimensional projections of the *N*_*sim*_ = 50 estimates and the confidence ellipsoid from *FI*_*MSS*_ for LV1. In general, there is a clear agreement between the estimates and the confidence ellipsoid, which is also reflected in the D- and E-criteria as well as the ARSE as summarized in Table 4 (first row).

**Table 4:** Different designs and their information content according to different optimality criteria.

Fully observed Lotka-Volterra model						
	Det(*FI*_*MSS*_)	Det(*FI*_*emp*_)	Min(EV(*FI*_*MSS*_))	Min(EV(*FI*_*emp*_))	ARSE(*FI*_*MSS*_)	ARSE(*FI*_*emp*_)
LV1	2. 10^16^	1.2 10^16^	4.1 10^3^	3.3 10^3^	(3%, 3%, 3%)	(3%, 3%, 4%)
LV2	2.1 10^18^	3. 10^18^	1.7 10^4^	2.1 10^4^	(1%, 1%, 1%)	(1%, 2%, 1%)
LV3	1.8 10^16^	7.4 10^15^	4.5 10^3^	2.7 10^3^	(3%, 3%, 3%)	(4%, 3%, 4%)
Partially observed Lotka-Volterra model						
LV4	3.7 10^13^	1.7 10^11^	1.2 10^−3^	5.1 10^−4^	(10%, 12%, 12%)	(14%, 15%, 16%)

Each row represents one experimental design and the columns show the number for the D-criterion from *FI*_*MSS*_ (column 1) and *FI*_*emp*_ (column 2), the E-criterion from *FI*_*MSS*_ (column 3) and *FI*_*emp*_ (column 4) and the ARSE (column 5 and 6).

Next, an extended observation time frame until *T* = 200 is considered retaining an inter-sample distance of Δ*t* = 1. This greatly increases accuracy of the estimates and one once again sees a good agreement for the confidence ellipsoids (Fig 10(LV2)) and the optimality criteria (Table 4(second row)). Furthermore, the ARSE is reduced by 50% yielding important information about the benefits in extending the observation time frame.

**Fig 11:**
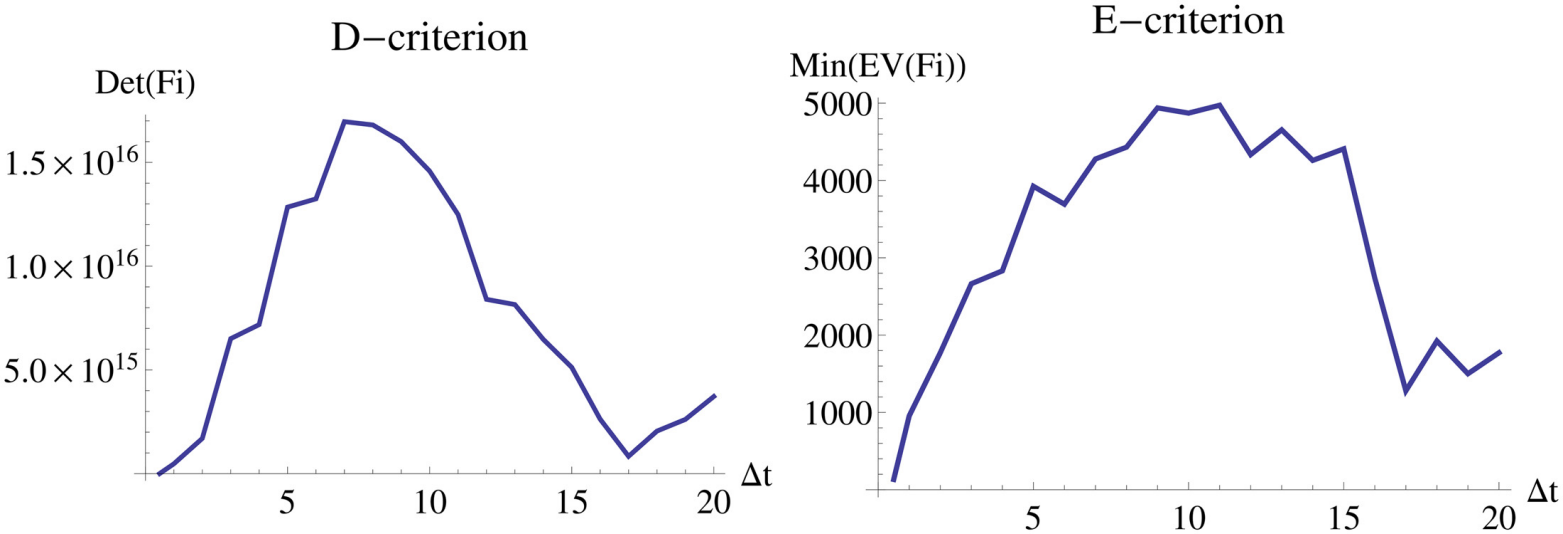
Dependence of D- and E-criterion on the inter-sample distance. The left panel shows interpolations of values of the D-criterion and the right panel of the E-criterion for different inter-sample distances.

To investigate whether experimental costs can be reduced by decreasing the number of measured time points while maintaining the information on the parameters, a scan over different inter-sample distances is performed based on an equidistant design with 10 observations (Fig 11). This scan is only performed for the *FI*_*MSS*_ Fisher information, a comparison to a covariance from estimates is omitted due to computational time requirements. Based on the optimality criteria, a sample distance of Δ*t* = 7 or Δ*t* = 9 would be preferable to Δ*t* = 1, which was used in LV1 and LV2.

Furthermore, there is no need to consider only equidistant designs rather than also allowing for experimental designs with varying inter-sample distances. Based on the D-criterion, an optimal design with 10 observations points is calculated. This leads to an optimization problem as in Eq (8) with the set of time points *T* as optimization variable. Here, a particle swarm algorithm is chosen for the optimization with 20 iterations, 25 particles and *M* = 500. The potential inter-sample distances are limited to be within 1 to 12, as the evaluation of the D- and E-criterion (Fig 11) does not suggest higher values. The resulting optimal design is denoted by LV3. It is assumed that the experimental set-up does not allow for arbitrary precision with respect to the observed time points and that observations may only be recorded at integer time points. The resulting optimal design consists of the time points *T* = (0, 7, 14, 22, 28, 36, 43, 47, 59, 66, 73). A three-fold reduction in observation time points has no impact on the accuracy as evident in the comparison of confidence ellipsoids and estimates (Fig 10LV3) and in the evaluation of optimality criteria (Table 4(third row). Depending on the experimental set-up, this is a huge reduction in costs. As the *FI*_*MSS*_ Fisher information captures this gain precisely, it is a valuable tool to reduce experimental costs.

**Fig 12:**
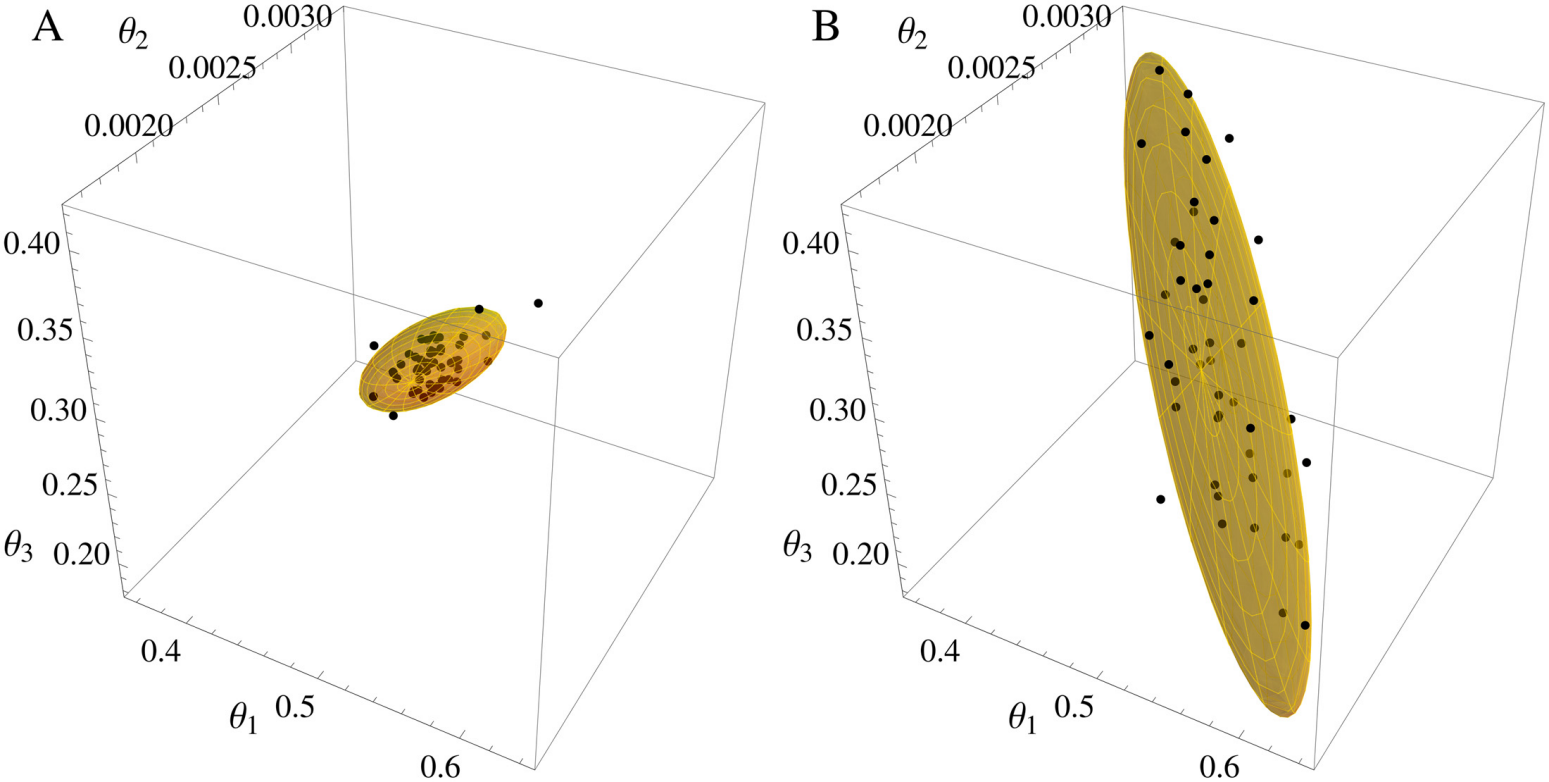
Parameter estimates and three dimensional confidence ellipsoid from the *FI*_*MSS*_ Fisher information for Lotka-Volterra model. Left panel fully observed scenario LV1, right panel partially observed scenario LV4. In each panel the black dots are the estimates from simulated data and the three dimensional confidence ellipsoid from *FI*_*MSS*_ is marked yellow. One can see that the *FI*_*MSS*_ Fisher information captures the change in correlation between the parameters.

The Lotka-Volterra model offers the chance to investigate scenarios with partial observation, namely scenarios in which only one species can be observed. The experimental design LV4 has the identical set-up as LV1, except that only the prey is observed. As Fig 10(LV4) demonstrates, the confidence ellipsoid of the new *FI*_*MSS*_ captures the shape of the estimates precisely even in partially observed scenarios. Interestingly, the correlation between the parameters *θ*_1_ and *θ*_2_ as well as *θ*_1_ and *θ*_3_ changes from positive in the fully observed scenarios to negative in the partially observed case. This is represented in the changed spatial orientation of the estimates’ cloud for LV4 when compared to the other scenarios in Fig 10. Again, the *FI*_*MSS*_ is able to capture this change. This is highlighted in Fig 12, where the three-dimensional ellipsoids are compared to the cloud of estimates for LV1 and LV4. Furthermore, the D- and E-criteria as well as the ARSE shown in Table 4 support the fact that the *FI*_*MSS*_ is even applicable in partially observed cases.

Table 5 summarizes the evaluation of the correlation between the parameters for different experimental designs. The fully observed designs (LV1-LV3) show moderate levels of correlation for the estimates, which is mapped by the *FI*_*MSS*_ Fisher information in the cases of LV1 and LV2. Due to the *FI*_*MSS*_ being an asymptotic measure a sparse sample of ten observed time points is likely the cause for a reduced precision in LV3. The design LV4 has a strong correlation between the second and third component of the parameter vector and the *FI*_*MSS*_ Fisher information captures this very well. Furthermore, the sign change of the correlation between the fully observed scenarios to the partially observed case is also represented in the *FI*_*MSS*_.

**Table 5:** Different experimental designs and their parameter correlation.

	Corr(*FI*_*MSS*_)	*Corr*_*emp*_
LV1	(0.54, 0.52, 0.54)	(0.59, 0.49, 0.59)
LV2	(0.54, 0.56, 0.57)	(0.57, 0.61, 0.71)
LV3	(0.47, 0.18, 0.29)	(0.63, 0.35, 0.53)
LV4	(-0.93, -0.9, 0.99)	(-0.89, -0.84, 0.97)

Correlation from Fisher information *FI*_*MSS*_ and correlation from estimates *Corr*_*emp*_. The three numbers in brackets correspond to *corr*(*θ*_1_, *θ*_2_), *corr*(*θ*_1_, *θ*_3_), *corr*(*θ*_2_, *θ*_3_).

The computational evaluation of the *FI*_*MSS*_ Fisher information matrix with *M* = 1000 takes approximately 22 minutes for LV1 and 2 hours for LV2 on an Intel Core i7-3770 CPU with 16GB RAM using one kernel. The increase in computational time by a factor of 5 is due to the length of the time series. As LV3 contains even fewer points than LV1, its computational time is even faster with 6 minutes. The computational time of LV4 is with 30 minutes slightly longer than LV1 as additional derivatives for the unobserved initial state have to be calculated.

#### Comparison with benchmark approach

The benchmark method [11] is applied to the LV1 scenario. First, the Fisher information *FI*_*Bench*_ is calculated and next 50 estimates from the simulated data set. These estimates are used to construct a covariance matrix and its inverse *FI*_*emp*,*Bench*_. Table 6 shows the comparison of D- and E-criterion as well as ARSE of *FI*_*Bench*_, *FI*_*emp*,*Bench*_, *FI*_*MSS*_ and *FI*_*emp*_. The accuracy of the parameter estimation with the benchmark is similar to the MSS (last column in Table 6). A Wilcoxon-Signed Rank test is applied to see whether the small differences in ARSE are significant. The benchmark is significantly better for *θ*_1_ in the scenario with 5 observations (0.1 ≥ *p* ≥ 0.01). The MSS method is significantly better for *θ*_1_ (20 observations), *θ*_2_ (20, 30 and 40 observations) and *θ*_3_ (30 and 40 observations) and strongly significantly better (*p* < 0.01) for *θ*_1_ (30 and 40 observations).

**Table 6:** Different designs and their information content according to different optimality criteria for the benchmark approach and the MSS approach.

Fully observed Lotka-Volterra model						
	Det(*FI*)	Det(*FI*_*emp*_)	Min(EV(*FI*))	Min(EV(*FI*_*emp*_))	ARSE(*FI*)	ARSE(*FI*_*emp*_)
Bench 5	1.2 10^12^	5.6 10^12^	3.4 10^2^	1.4 10^2^	(6.3%, 36.9%, 16.9%)	(9.%, 21.3%, 26.5%)
MSS 5	3.5 10^12^	2.9 10^12^	1.1 10^2^	9.5 10^1^	(9.6%, 19.4%, 29.1%)	(11.8%, 23.2%, 31.4%)
Bench 10	1. 10^14^	8 10^14^	1.9 10^3^	1.1 10^3^	(3.8%, 15.9%, 6.2%)	(5.%, 4.7%, 6.9%)
MSS 10	4.8 10^14^	4.7 10^14^	9.6 10^2^	8.5 10^2^	(5.4%, 5.4%, 7.6%)	(5.7%, 5.9%, 7.5%)
Bench 20	1.4 10^15^	1.7 10^15^	6.6 10^3^	1.7 10^3^	(2.4%, 8.9%, 3.5%)	(4.6%, 5.1%, 4.7%)
MSS 20	1.9 10^15^	1.3 10^15^	1.8 10^3^	1.3 10^3^	(4.3%, 4.6%, 4.6%)	(4.7%, 5.2%, 5.7%)
Bench 30	2.6 10^21^	3 10^15^	5.2 10^4^	1.3 10^3^	(0.5%, 0.1%, 1.3%)	(4.7%, 5.7%, 5.4%)
MSS 30	1.1 10^16^	7.9 10^15^	3.2 10^3^	2.9 10^3^	(3.2%, 3.2%, 3.6%)	(3.3%, 3.4%, 4.%)
Bench 40	1.6 10^35^	3.7 10^15^	1.6 10^7^	1.5 10^3^	(0.%, 0.%, 0.%)	(4.4%, 5.9%, 5.2%)
MSS 40	2. 10^16^	1.2 10^16^	4.1 10^3^	3.3 10^3^	(2.9%, 3.%, 3.2%)	(3.2%, 3.2%, 3.9%)

Each row represents an experimental design and a method. The columns show the number for the D-criterion of *FI*_⋅_ (column 1) and *FI*_*emp*,⋅_ (column 2), the E-criterion of *FI*_⋅_ (column 3) and *FI*_*emp*,⋅_ (column 4) and the ARSE (column 5 and 6).

More importantly, the benchmark Fisher information matrix *FI*_*Bench*_ exhibits a strong overestimation of the precision for larger observation horizons (30 and 40) as its values for the D- and E-criterion are a lot higher than the corresponding values of *FI*_*emp*,*Bench*_. The same holds for the parameter individual ARSEs which are strongly underestimated.

**Fig 13:**
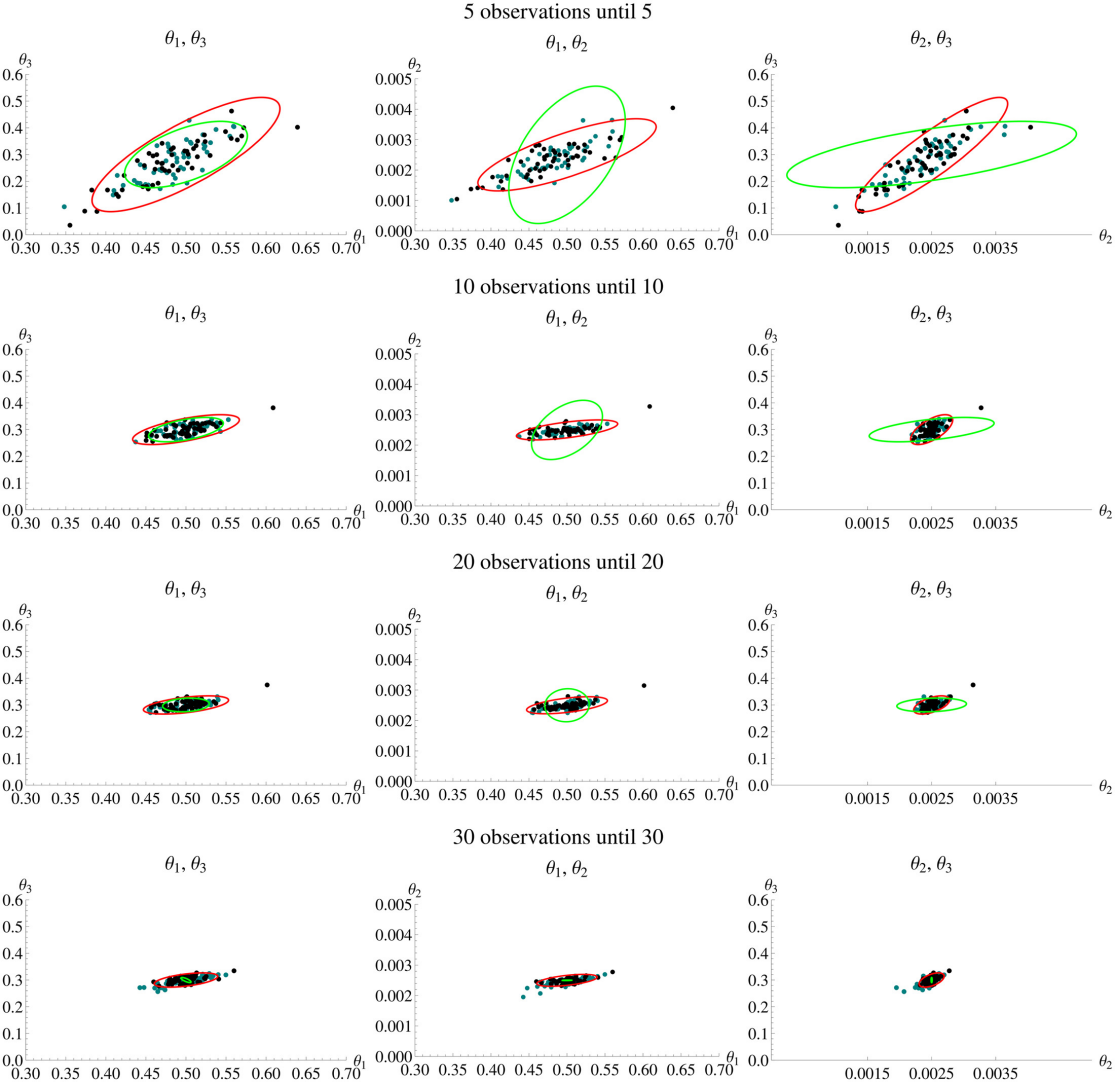
Parameter estimates and confidence ellipsoid MSS and the benchmark. Each row shows an experimental design. In each row, each panel shows one two dimensional projection of the three dimensional parameter space. In each graphic the black dots are the estimates from MSS and the green dots from Bench. The confidence ellipsoid of *FI*_*MSS*_ is marked red and the confidence ellipsoid of *FI*_*Bench*_ green. The confidence ellipsoid of *FI*_*Bench*_ for the last row with 30 observations is so small that it can be hardly seen.

Fig 13 shows the 2-dimensional projections of the parameter estimates for MSS and benchmark. For small observation horizons (≤ 20) the *FI*_*Bench*_ performs slightly worse than the MSS method but it roughly captures the location of the estimates. However, for larger observation horizons (30 and 40) the 2-dimensional confidence ellipsoid of *FI*_*Bench*_ is too small to capture the location of the estimates. Table 7 shows that this is not only a problem of the size of the ellipsoid but also the correlations, as they are also not well reflected.

**Table 7:** Different experimental designs and their parameter correlation for MSS and the benchmark.

	Corr(*FI*_*Bench*,*MSS*_)	*Corr*_*Bench*,*emp*_	*FI*_*MSS*_	*FI*_*emp*_
5	(0.5, 0.56, 0.62)	(0.84, 0.80, 0.87)	(0.78, 0.8, 0.89)	(0.85, 0.88, 0.87)
10	(0.46, 0.52, 0.53)	(0.68, 0.62, 0.52)	(0.61, 0.65, 0.64)	(0.61, 0.74, 0.66)
20	(0.04, 0.17, 0.17)	(0.67, 0.54, 0.54)	(0.54, 0.53, 0.55)	(0.64, 0.66, 0.66)
30	(0.27, -0.77, 0.)	(0.76, 0.82, 0.79)	(0.55, 0.52, 0.54)	(0.55, 0.5, 0.58)
40	(-0.19, 0.55, -0.09)	(0.75, 0.82, 0.77)	(0.54, 0.52, 0.54)	(0.6, 0.5, 0.63)

First column: number of observations with *Deltat* = 1, second column: correlation from *FI*_*Bench*_, third column: correlation from estimates *Corr*_*Bench*,*emp*_, fourth column: correlation from *FI*_*MSS*_ and fifth column *FI*_*emp*_. The three numbers in brackets correspond to *corr*(*θ*_1_, *θ*_2_), *corr*(*θ*_1_, *θ*_3_), *corr*(*θ*_2_, *θ*_3_).

The striking difference in performance can be explained as follows: Both methods rely on approximations, namely, the MSS method on an interval-wise LNA and the benchmark on a LNA on the whole systems horizon. As mentioned in the introduction, the second is a lot more restrictive than the first. Whether the approximation holds, can be easily tested. The benchmark’s approximation requires *ν* ∼ *MVN*(*μ*, Σ_*B*_) (see Eq 19). If this is fulfilled, then it follows that (*ν* − *μ*)*A*_*B*_ with ${\left(A_{B}^{\prime }A_{B}\right)}^{-1}=\Sigma_{B}$ is a vector of independent standard normally distributed random variables. A Kolmogorov-Smirnov test can be applied to test this. Similarly, the MSS methods requires *ν*_*i*_ ∼ *N*(*x*(Δ_*i*_; *θ*, *ν*_*i*−1_), Σ(Δ_*i*_; *θ*)) for *i* = 1, …, *n* which leads to (*ν*_*i*_ − *x*(Δ_*i*_; *θ*, *ν*_*i*−1_))*A*, with (*A*′ *A*)^−1^ = Σ(Δ_*i*_; *θ*) for *i* = 1, …, *n*. This can be also tested by a Kolmogorov-Smirnov test.

**Fig 14:**
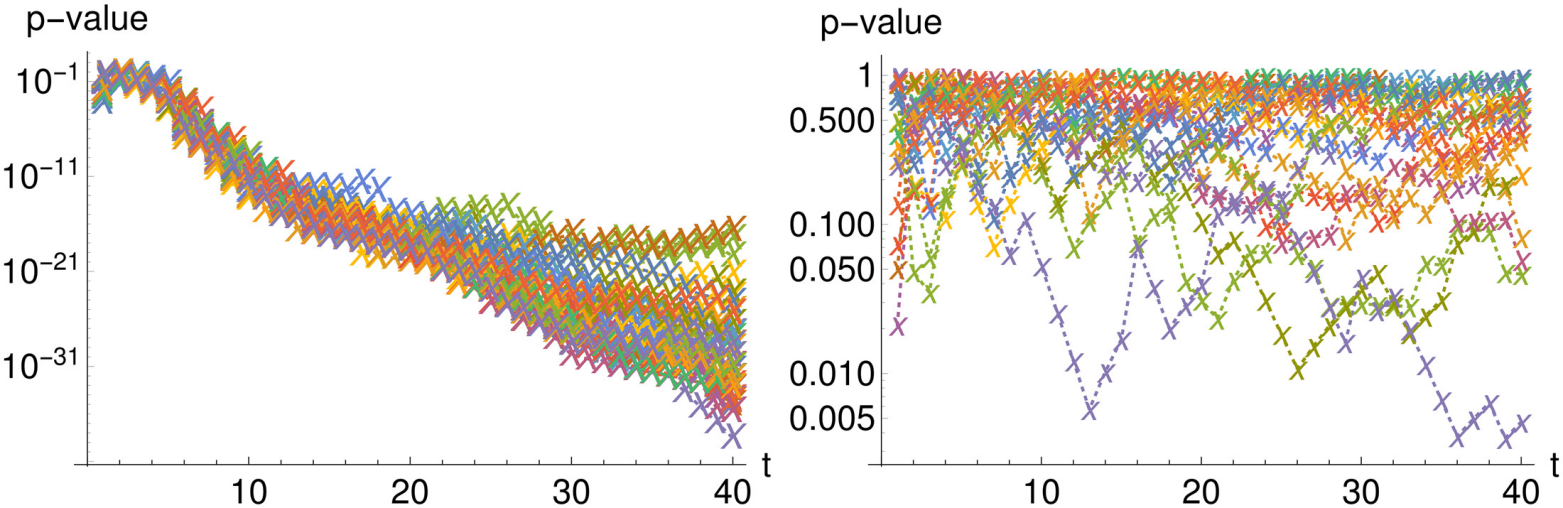
Testing the approximation for different observations horizons. P-values for Kolmogorov-Smirnov tests whether the approximation is fulfilled for different observation horizons; left panel shows results for benchmark and right panel for MSS. Each color stands for one of the 50 data sets. Test is performed with the true parameter *θ* = (0.5, 0.0025, 0.3).

Fig 14 shows the p-values of the Kolmogorov-Smirnov test for the benchmark (left panel) and the MSS (right panel) in dependence of the total observation horizon. One can clearly see that the MSS methods assumption is not significantly violated but the benchmark’s assumption clearly fails with increasing observation duration. This shows the strong benefits of applying the multiple shooting approach and using the LNA only on the intervals between observations. Fig 14, therefore, also explains the differences in performance for the benchmark Fisher information *FI*_*Bench*_ and the MSS Fisher information *FI*_*MSS*_.

**Fig 15:**
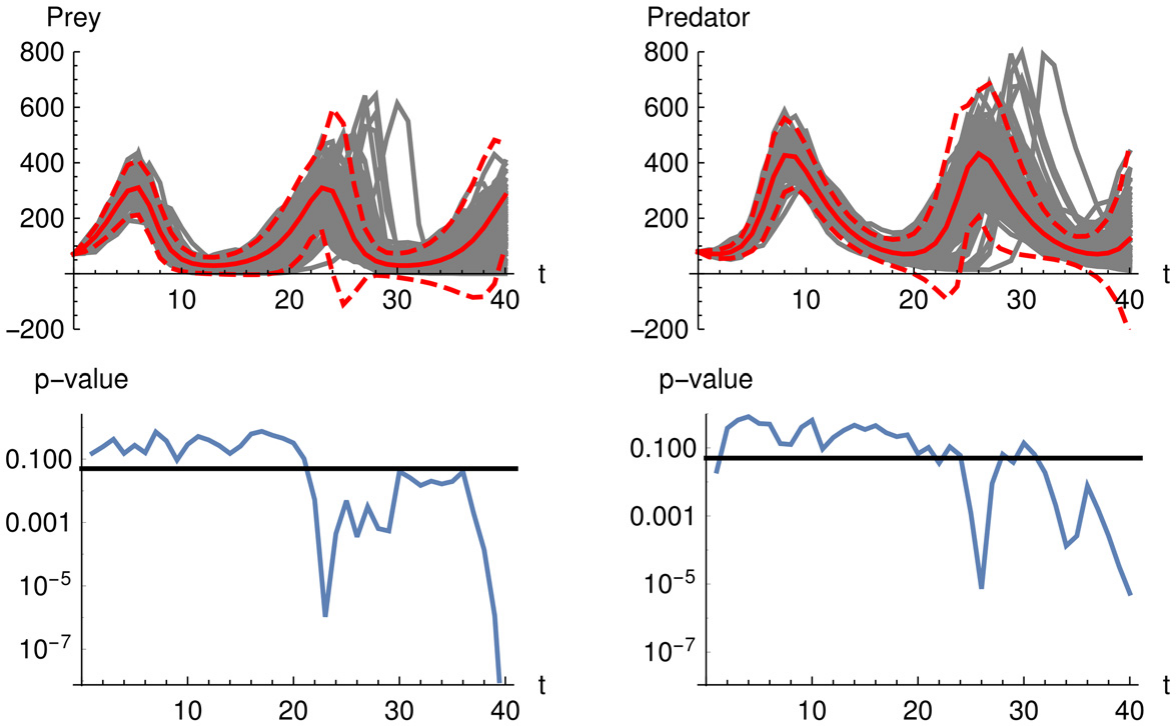
Mean and two standard deviations from LNA versus stochastic simulations for Lotka-Volterra model. The upper row shows 100 stochastic simulations in gray color and the mean from a LNA in solid red color as well as mean plus and minus two standard deviations in dashed red color. The lower row shows p-values of a Kolmogorov-Smirnov test for each time point whether the 100 stochastic simulations follow a normal distribution with mean and variance from a LNA. The solid line at a p-value of 0.05 illustrates that all values below show significant differences to the LNA approxiamtion. One can see that the quality of the LNA approximation decreases over time. Test is performed with the parameter *θ* = (0.5, 0.0025, 0.3).

Fig 15 shows the comparison of the mean and the mean plus and minus two standard deviation calculated from the LNA and 100 stochastic simulations over time demonstrating why the performance of the LNA decreases with increasing observation duration. The LNA yields an accurate approximation in the beginning (until time 20) but does not lead to an accurate description for any later time points (from time 20 onwards).

### Calcium oscillation model

The third model used to evaluate the new approach is a Calcium oscillation model [36]:

$$\begin{array}{cc} \frac{d\;G(t)}{dt}\; & =\;\theta_{1}+\theta_{2}G\left(t\right)-\frac{\theta_{3}\;G\left(t\right)\;Ca\left(t\right)}{G\left(t\right)+\theta_{4}}-\frac{\theta_{5}\;G\left(t\right)\;PLC\left(t\right)}{G\left(t\right)+\theta_{6}}\\ \frac{d\;PLC(t)}{dt}\; & =\;\theta_{7}G\left(t\right)-\frac{\theta_{8}\;PLC\left(t\right)}{PLC\left(t\right)+\theta_{9}}\\ \frac{d\;Ca(t)}{dt}\; & =\;\theta_{10}G\left(t\right)-\frac{\theta_{11}\;Ca\left(t\right)}{Ca\left(t\right)+\theta_{12}}. \end{array}$$ (33)

where *Ca*(*t*) stands for cytosolic Calcium, *G*(*t*) for the active subunit of the G-protein and *PLC*(*t*) for the activated form of phospholipase C [36]. The behavior of this model differs qualitatively between stochastic and deterministic modeling for small particle numbers as presented in [36]. The true parameter vector is

θ=(212,2.95,1.52,190,4.88,1180,1.24,32240,29090,13.58,153000,160) (34)

and the initial value is (*Ca*, *G*, *PLC*)(0) = (10, 10, 10). This model shows highly nonlinear oscillations in stochastic modeling but only small amplitude regular oscillations in deterministic modeling (Fig 1). Therefore, this model is excellent for testing any methods analyzing models with intrinsic stochasticity. Even more, Calcium oscillations are also of a high practical relevance: in cell development and death as well as fertilization [29].

**Fig 16:**
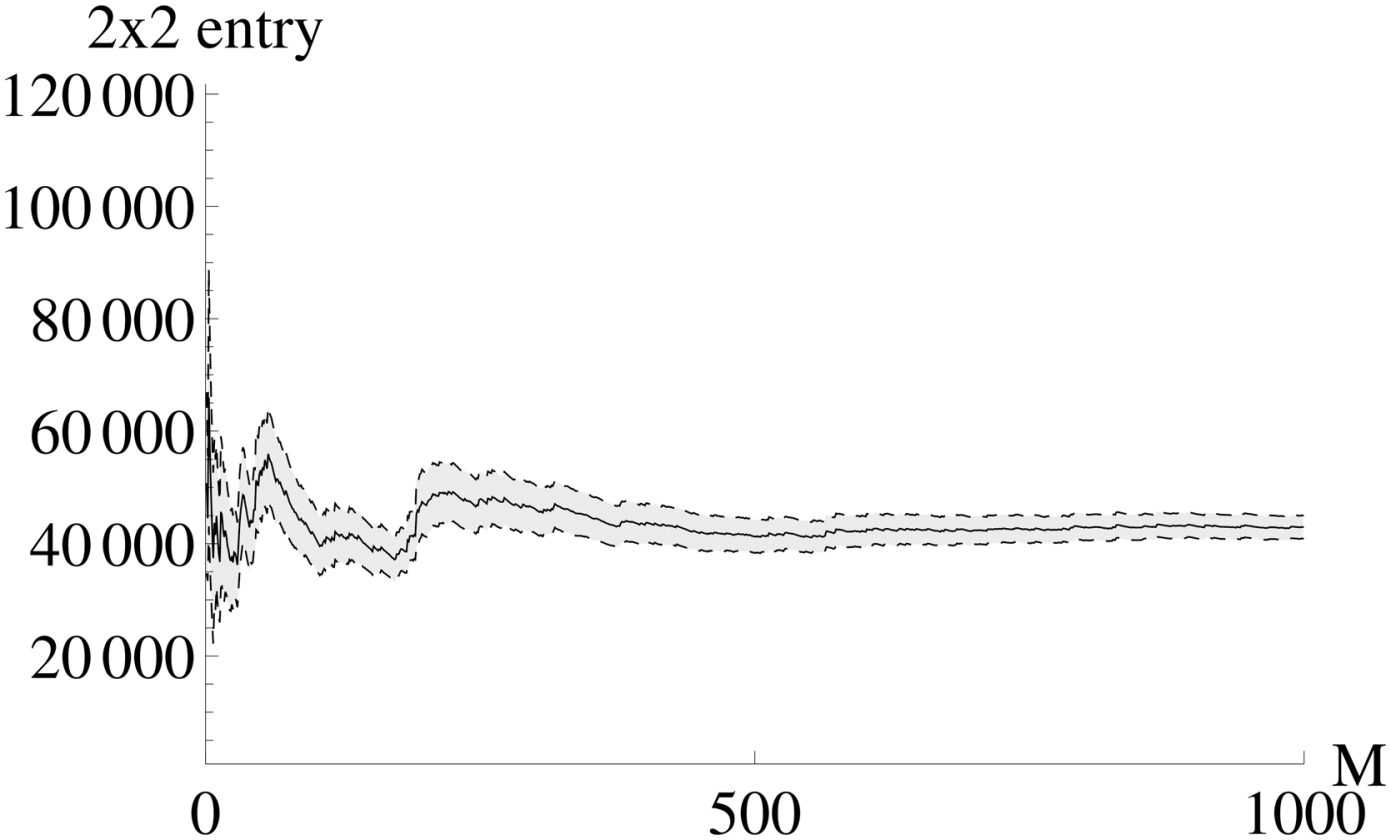
Dependence of the accuracy of the *FI*_*MSS*_ entries on the number of pseudo data sets. The x-axis shows the number of pseudo data sets *M* used for calculating the 2 × 2 entry of *FI*_*MSS*_, the mean is shown as solid line. Gray color shows the area from sample mean plus / minus one standard deviation. As the width of the gray area is decreasing, the accuracy is increasing. One can see that already small values as *M* = 400 give a good approximation.

Even though the systems is highly nonlinear, the *FI*_*MSS*_ can be calculated with a moderate number of *M* = 400 pseudo data sets, as representatively shown for the 2 × 2 entry in Fig 16. The remaining entries of the *FI*_*MSS*_ can be found in S3 Fig. The fact that the *FI*_*MSS*_ can be calculated with a moderate number of pseudo data sets even in highly nonlinear systems is essential as otherwise the computational costs would be to high for performing experimental design, which needs multiple *FI*_*MSS*_ calculations. The respective plots for the partially observed case can all be found in S4 Fig.

**Fig 17:**
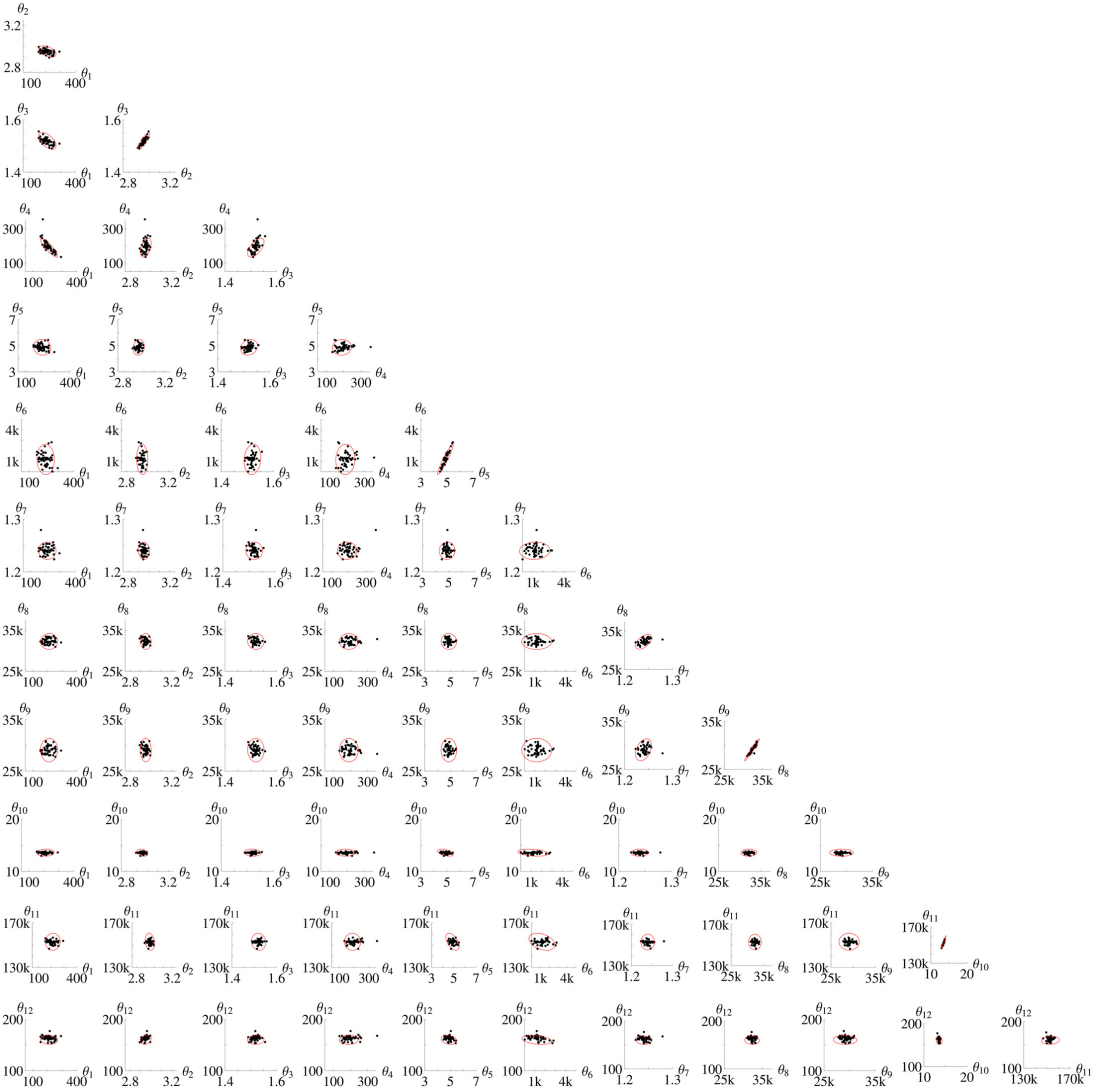
Parameter estimates and confidence ellipsoid from *FI*_*MSS*_ for Calcium model. The panels show the two dimensional projections of the 12-dimensional parameter space for the Δ*t* = 0.5 design. In each panel the black dots are the estimates from simulated data and the confidence ellipsoid of the Fisher information is marked red. “k” is used as an abbreviation for “thousand”.

Fig 17 shows the consensus between the *FI*_*MSS*_ Fisher information matrix and the 2-dimensional projections of the cloud of the *N*_*sim*_ = 50 estimates from simulated data. Each panel shows one 2-dimensional projection of the parameter space. As with the previous models, there is a nice agreement between the *FI*_*MSS*_ Fisher information and the shape and the size of the cloud of estimates for all projections.

Table 8 shows the consensus between *FI*_*MSS*_ and *FI*_*emp*_ on the D- and E-criterion. The *FI*_*MSS*_ Fisher information captures the volume reduction of the confidence ellipsoid (D-criterion) with larger inter-sample distance. The *FI*_*MSS*_ Fisher information also demonstrates robustness of the minimal eigenvalue (E-criterion) towards changes in the inter-sample distance. This means that the newly defined *FI*_*MSS*_ Fisher information matrix is able to capture key properties of experimental design in systems with highly stochastic oscillations. The fact that the D-criterion changes, while the E-criterion is almost constant over the three experimental designs, means that the volume of the confidence ellipsoid reduces while its axis in the parameter space with the smallest precision does not improve. A similar effect has been observed for the Immigration-Death model with designs ID-Δ*t* = 1.0 and ID-Δ*t* = 7.5 in Fig 3.

**Table 8:** D- and E-criterion for different experimental designs for the Calcium oscillation model.

		Δ*t* = 0.1	Δ*t* = 0.2	Δ*t* = 0.5
D-criterion	Det(*FI*_*MSS*_)	2.6 10^−14^	1.4 10^−11^	1.2 10^−9^
	Det(*FI*_*emp*_)	5.3 10^−15^	4.1 10^−13^	6.6 10^−10^
E-criterion	Min(EV(*FI*_*MSS*_))	1.4 10^7^	8.5 10^6^	9.8 10^6^
	Min(EV(*FI*_*emp*_))	9.3 10^6^	7.4 10^6^	3.3 10^6^

Each column shows one experimental design.

An investigation of the ARSE for the three different designs (Table 9) shows that the *FI*_*MSS*_ performs equally well for all three designs.

**Table 9:** ARSE of *FI*_*MSS*_ and of the 50 estimates for experimental designs in Calcium oscillation model.

Design	Δ*t* = 0.1		Δ*t* = 0.2		Δ*t* = 0.5	
Parameter	FI Δ*t* = 10	Exp Δ*t* = 10	FI Δ*t* = 20	Exp Δ*t* = 20	FI Δ*t* = 50	Exp Δ*t* = 50
*θ*_1_	13.3%	14.7%	11.%	12.6%	11.2%	14.8%
*θ*_2_	1.%	0.8%	0.9%	0.7%	0.7%	0.8%
*θ*_3_	1.3%	2.3%	1.1%	1.8%	0.9%	0.8%
*θ*_4_	15.8%	104.4%	11.4%	81.%	11.8%	20.%
*θ*_5_	5.2%	6.8%	5.5%	4.1%	5.%	4.3%
*θ*_6_	49.5%	66.9%	54.2%	41.5%	50.3%	49.6%
*θ*_7_	1.4%	1.4%	0.8%	1.%	0.5%	0.8%
*θ*_8_	6.7%	5.3%	3.7%	4.7%	2.4%	2.%
*θ*_9_	10.8%	8.7%	5.1%	7.4%	3.2%	2.6%
*θ*_10_	1.8%	1.5%	1.8%	1.4%	2.%	1.2%
*θ*_11_	1.8%	1.5%	1.9%	1.4%	2.%	1.2%
*θ*_12_	2.2%	2.4%	2.2%	2.3%	2.2%	3.2%
**Median**	**3.7%**	**3.9%**	**2.9%**	**3.2%**	**2.3%**	**2.3%**

The table displays the ARSE gained from the *FI*_*MSS*_ and the ARSE from *FI*_*emp*_ for each of the parameters (rows) and each of the experimental design (columns). The last row gives the median of the components *θ*_1_ to *θ*_12_. The *FI*_*MSS*_ Fisher information matrix captures all ARSEs well.

In contrast to the D-criterion, which improves with increasing inter-sample distance, the ARSE remains fairly constant. A similar phenomenon could be observed in the Immigration-Death model (Fig 3) where the ARSE is fairly the same for Δ*t* = 0.5 and Δ*t* = 7.5 while the volume is a lot smaller for the second design.

During the analysis of the Calcium model one potential drawback of the LNA based MSS objective function was encountered. The LNA approximation in the MSS objective function breaks down for large inter-sample distances as the influence of nonlinear effects on the dynamics increases. In addition to the theoretical condition on the LNA—discussed in detail in [13]—there is an easy way to detect such situations: Calculate *M*_1_ = *FI*_*MSS*_(*ϑ*, *T*) and *M*_2_ = *FI*_*MSS*_(*ϑ*, *T*′) with designs *T* = (*t*_0_, *t*_1_, *t*_2_, …, *t*_*n*_) and $T^{\prime }=\left(t_{0},\frac{t_{0}+t_{1}}{2},t_{1},\frac{t_{1}+t_{2}}{2},t_{2},\ldots ,t_{n-1},\frac{t_{n-1}+t_{n}}{2},t_{n}\right)$. If the LNA approximation holds, the entries of *M*_1_ and *M*_2_ should have a similar size. Therefore, the quotients of the diagonal elements of *M*_1_ and *M*_2_ are calculated for comparison. The mean and two standard deviations serve as an indication how close these values are to 1 (which would indicate a similar size). If they are close to one, this means that the choice of the time step does not influence the result. Their mean and standard deviation are for Δ*t* = 0.10: 1.02 ± 0.20, for Δ*t* = 0.20: 1.00 ± 0.15, for Δ*t* = 0.50: 0.72 ± 0.48. The LNA does not hold any more for Δ*t* = 1.0: 4.9 10^−5^ ± 1.9 10^−4^, Δ*t* = 1.5: 2.2 10^−8^ ± 3.9 10^−8^ and Δ*t* = 2.0: 1.0 10^−9^ ± 6.0 10^−10^. Therefore, experimental designs with inter-sample distances of Δ*t* = 1.0 or higher cannot be recommended. For the Lotka-Volterra and Immigration-Death model, there was no such indication and the LNA approximation was valid for all considered step-sizes.

The Calcium model also indicates that it is more consistent to use the scheme of Eq (16) for generating the pseudo data than the Gillespie algorithm. As even the interval-wise LNA becomes critical with longer inter-sample distance, one can either use a rough model approximation with the MSS and then calculate the Fisher information consistently with the scheme or use the correct (Gillespie) model and use a rough approximation for the Fisher information. As the calculation of the Fisher information includes derivatives, the first seems to be more robust towards rough approximations. S7 Fig creates the same plot as in Fig 17 but with Gillespie simulations instead of the scheme. The result shows still a good agreement of *FI*_*emp*_ and *FI*_*MSS*_ but the use of the scheme is favorable as the consistence between *FI*_*emp*_ and *FI*_*MSS*_ is better in Fig 17. The D-criterion for *FI*_*MSS*_ with the Gillespie algorithm is 1.1 × 10^−6^, which underlines that the scheme is more suited (Table 8 shows that the D-criterion *FI*_*emp*_ is 6.6 10^−10^ and the D-criterion of *FI*_*MSS*_ with the scheme is 1.2 10^−9^). Whenever the interval-wise LNA is not rough, there is no statistic difference between pseudo data from the scheme or the Gillespie algorithm, so the choice does not matter.

**Fig 18:**
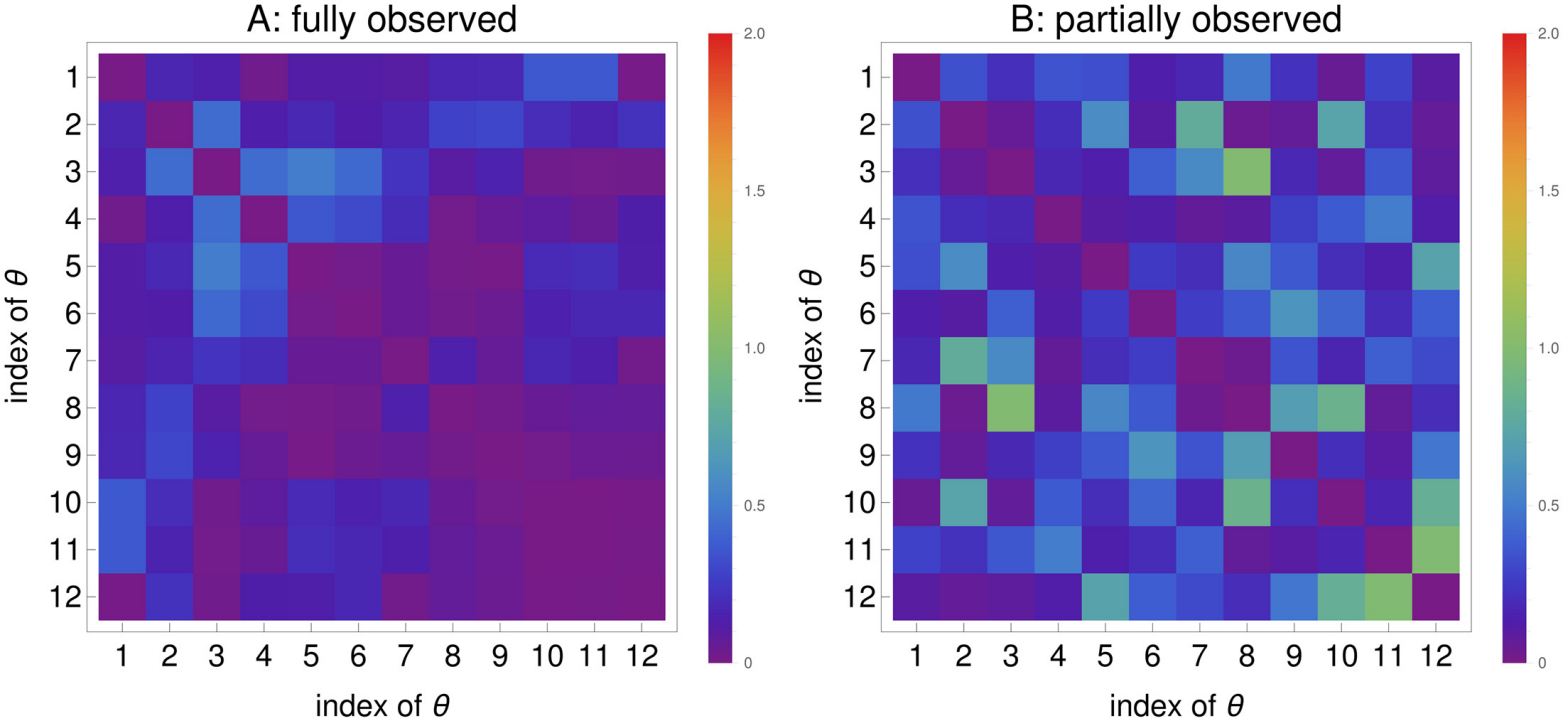
Heat map of the difference of correlations between Corr(*FI*_*MSS*_) and *Corr*_*emp*_. A shows the fully observed Calcium oscillation model and B the partially observed scenario. Two correlation matrices are calculated for each of the cases, one from *FI*_*MSS*_ and the other from the estimates. The absolute value of their differences is color-coded.

To evaluate the parameter correlation calculated by the *FI*_*MSS*_, correlation matrices are composed of the *FI*_*MSS*_ and the estimates for a design with an inter-sample distance of Δ*t* = 0.1. The consensus is evaluated based on the absolute values of their difference, which is illustrated in Fig 18(A).

The computational time for an evaluation of the Fisher information matrix with *M* = 1000 takes roughly 6 hours on an Intel Core i7-3770 CPU with 16GB RAM using eight kernels.

The state estimation procedure for the partially observed case has been developed and shown to work well previously [14]. However, in highly nonlinear stochastic models with few observables, imprecise state estimates are in principle possible. [14] (Fig 5) shows that, even in such case, the method can extract information from current data and re-adapt the estimates of the unobservables to their underlying dynamics. Nevertheless, it means that there is at least one poor state estimate which might have resulted in one very unlikely transition to the following data point. This unlikely transition leads to a very small probability, which results in a very high negative log-likelihood value and in an unrealistically high term of the Fisher information matrix.

To circumvent this issue, the transitions between time points *t*_*i*−1_ and *t*_*i*_ are determined for which $q_{15}<((O_{i}-x(\Delta_{i};\theta ,{\hat{\nu }}_{i-1})\text{)}{\left(\Sigma (\Delta_{i};\theta )+\Sigma^{\text{meas}}\right)}^{-1/2}{)}_{k}<-q_{15}$ holds for all observed components *k*, with *q*_15_ being the 10^−15^ quantile of a standard normal distribution. For time steps that do not fulfill this condition, the corresponding components of the MSS objective function derivatives are set to zero and not counted for *FI*_*MSS*_ which means that these time intervals are disregarded. Using the 10^−15^-quantile means that only very strong outliers are not counted as information in the *FI*_*MSS*_ Fisher information matrix. In fact, calculating the *FI*_*MSS*_ for a scenario in which only Calcium is observable, this happened on average for 2.8 in 100 intervals over the *M* = 1000 pseudo data sets; for a scenario in which both Calcium and *PLC* are observable only 0.5 in 100 failed this condition. No occurrences were detected in the partially observed Lotka-Volterra system. Furthermore, the components of the state estimates $\hat{\nu }$ are lower bounded with 0.1 for numerical reasons.

Investigating the ARSE for the partially observed scenario showed a good agreement between the *FI*_*MSS*_ and the estimates for most of the parameters (Table 10). The deviations for e.g. *θ*_10_ and *θ*_11_ might indicate that the landscape of the parameter space contains some strong nonlinearities which cannot be captured by the Fisher information matrix as it is, by definition, a quadratic measure. These nonlinearities could lead to non-identifiabilities that are not captured by the Fisher information. A similar phenomenon has been observed previously [42] and the use of the profile likelihood techniques [43] has been suggested to improve the analysis.

**Table 10:** ARSEs from estimates and from *FI*_*MSS*_ for partially observed Calcium oscillation model.

Design	only Ca observable		Ca and PLC observable	
Parameter	*FI*_*MSS*_	*FI*_*emp*_	*FI*_*MSS*_	*FI*_*emp*_
*θ*_1_	16.9%	43.6%	11.7%	24.3%
*θ*_2_	1.9%	6.7%	0.6%	1.8%
*θ*_3_	3.9%	16.9%	0.7%	5.%
*θ*_4_	12.4%	57.6%	7.4%	40.2%
*θ*_5_	3.3%	42.5%	5.7%	7.3%
*θ*_6_	29.3%	121.2%	47.2%	97.2%
*θ*_7_	13.5%	17.9%	0.6%	7.2%
*θ*_8_	45.8%	15.4%	2.5%	2.8%
*θ*_9_	35.9%	26.3%	3.2%	3.4%
*θ*_10_	1.5%	955.7%	1.5%	6.4%
*θ*_11_	1.1%	20.2%	1.4%	2.7%
*θ*_12_	2.8%	15.7%	3.4%	4.9%
**Median**	**8.1%**	**23.3%**	**2.8%**	**5.7%**

Same notation as in Table 9.

However, the important gain from this analysis is that the *FI*_*MSS*_ Fisher information matrix can be used to compare the three designs, fully observed, Calcium and PLC observed, and only Calcium observed. This comparison leads to the insight that the additional measurement of PLC gives a modest increase in accuracy compared to only measuring Ca. The further additional measurement of G (leading to a full observation) does not have a remarkable impact on the information and accuracy anymore. This means that one can easily save the cost of measuring G. Depending on the costs for measuring PLC, a compromise between accuracy and cost can be reached. The newly suggested *FI*_*MSS*_ Fisher information matrix covers these differences well and can, therefore, serve as a valuable instrument in deciding on the design of an experiment.

The correlation structure between the parameters can also be reproduced as shown in Fig 18B for the scenario with observation of Calcium and PLC.

## Discussion

This work introduces an approach to calculate a *FI*_*MSS*_ Fisher information matrix for stochastic models based on the MSS objective function [14]. The *FI*_*MSS*_ approach is able to successfully capture important experimental design properties such as precision and correlation in challenging models. Furthermore, it allows the comparison of the information content of different experimental design and, by that, choose an optimal design. The article demonstrates that these features hold for highly nonlinear models that might even show a qualitatively different behavior in stochastic modeling than in deterministic modeling. Therefore, the method is particularly suited for application on signaling pathways in systems biology.

The calculation of the *FI*_*MSS*_ Fisher information is based on the MSS objective function [13, 14]. The MSS objective function treats the intervals between succeeding observations separately. On each interval a LNA is used and the unobserved states are updated with a state estimation procedure. The *FI*_*MSS*_ Fisher information is calculated based on this MSS objective function and the use of pseudo data which is gained by the same MSS approximation.

The dependency of the *FI*_*MSS*_ precision on the number of pseudo data sets used for the calculation was investigated. As illustrated in Figs 2 and 16 and S1 Fig to S4 Fig, a few hundred pseudo data sets are sufficient to obtain a good approximation. As there are many evaluations of the Fisher information matrix involved in finding the optimal experimental design, this is a critical characteristic of the new method.

The Fisher information is an asymptotic description of the inverse of the covariance matrix of a maximum likelihood estimator. In particular in the stochastic case it is also approximative. Thus, it is very important to investigate whether its accuracy is still satisfactory under realistic (particularly finite) data scenarios. Therefore, this work compares the *FI*_*MSS*_ Fisher information matrix to *FI*_*emp*_, the inverse of a covariance matrix calculated from parameter estimates. These parameter estimates are gained by performing parameter estimations on simulated data sets. Both, *FI*_*MSS*_ and *FI*_*emp*_, are then compared based on

- two-dimensional projections of the confidence ellipsoids and the estimates—which is easiest for visualization,
- optimality criteria such as determinant (corresponding to volume of confidence ellipsoid) and minimal eigenvalue (corresponding to the largest axis of the confidence ellipsoid),
- average relative squared errors and
- the correlation structure.

All this is solely done to evaluate the accuracy of the suggested methodology. There is no need for the comparison in real life applications where it is enough to calculate the *FI*_*MSS*_ Fisher information matrix for designing experiments.

Three test models were used to demonstrate the power of the newly suggested *FI*_*MSS*_ Fisher information matrix: an Immigration-Death model, a Lotka-Volterra model, and a Calcium oscillation model. The newly defined *FI*_*MSS*_ Fisher information matrix proved to be successful for all four test measures (a-d). Figs 3, 10 and 17 show that it reflects the shape and size of the two-dimensional projections of the confidence intervals. Furthermore, Fig 4 and Tables 4 and 8 show that it covers the volume (D-criterion) and the largest axis (E-criterion) of the multi-dimensional confidence ellipsoid. The average relative squared error (ARSE) is covered precisely as well (Fig 5 and Tables 4+9). Additionally, the correlation structure is reflected accurately (Tables 1 and 5 and Fig 18).

During the evaluation of the Immigration-Death model a larger optimal inter-sample distance was obtained based on the D-criterion compared to the ARSE (Fig 4). Depending on the experimenter’s interest, the newly introduced *FI*_*MSS*_ MSS Fisher information matrix aids in choosing an appropriate experimental design. The analysis of the Lotka-Volterra model demonstrates the gain in precision by extending the observed time frame (five-fold from LV1 to LV2). An even greater gain can be achieved by allowing for non-equidistant designs. Here, a similar amount of information can be obtained with 10 observations compared to 40 observation at equidistant time intervals. Depending on the experimental set-up, this is a huge reduction in costs. The Calcium model showed that there is an increase in information when measuring PLC and Calcium instead of only Calcium. Measuring also G (hence all three variables) does not lead to a strong increase in information anymore. As this analysis can be run before performing any experiments, the Fisher Information is a very valuable tool for experimental design. The *FI*_*MSS*_ Fisher information matrix extended its applicability to signaling pathways with high nonlinearity and intrinsic stochastic effects that lead to a qualitatively different behavior from the deterministic solution.

The approach of [28] uses the expected Kullback Leibler divergence between prior and posterior distribution to measure the information content of an experiment. The potential lack of prior knowledge on the parameter can be handled with an uninformative prior. This is an advantage compared to the MSS method using the Fisher information matrix, which is a parameter dependent measure. However, [28] uses Monte Carlo simulations thrice to explore a) the parameter space, b) the observation space and c) the state space. While the additional computational cost of a) leads to a broader applicability (in case of poor prior knowledge regarding the parameter) and the cost of b) is comparable to the MSS method’s simulation cost, the additional computational cost for c) is a critical advantage of the MSS method, especially in signaling pathways with fast dynamics and a huge state space such as the Calcium model (in which the states of all three components take values from 0 to 10 000 within a few time units).

[27] is suited for an experimental set up with multiple measurements per time point comparing their moments with parametrized theoretical moments based on a moment closure without the use of simulations. The MSS approach differs from this approach as it is suited to experiments with measurements from only one time course (and not multiple measurements per time point) and it uses simulations to generate the pseudo data for the *FI*_*MSS*_. Next, MSS employs a LNA in contrast to moment closure, see [44] for a comparison of LNA and moment-closure which are both used to calculate moments of stochastic systems.

In contrast to other recent approaches from [11, 25], the *FI*_*MSS*_ Fisher information matrix only needs the LNA on the relatively short time interval between two succeeding measurement points. This makes it less restrictive than a LNA on the whole time horizon as a comparison with a benchmark ([11]) has shown. This benchmark treats the observations as samples from a multivariate normal distribution with a mean equaling the deterministic solution and a covariance matrix containing all inter temporal covariances. The LNA is applied to calculate these inter-temporal covariances. If the system can be approximated with a LNA over the whole time horizon, the benchmark approach has two advantages: a) it allows to consider the inter-temporal correlations which provide additional information that cannot be exploited with the MSS method and b) it does not need Monte Carlo simulations for the calculation of a Fisher information matrix. It needs only one ODE solution and one calculation of the inter-temporal covariances, which is an increase in computational speed. This is also of benefit for parameter estimation because [11] needs only one computation of the ODE and of the inter-temporal covariance system independent of the number of single-cell trajectories. However, the number of rows and columns of the inter-temporal covariance matrix scales with the product of the number of time points and the number of components.

The results of the Immigration-Death model show a similar performance of MSS and the benchmark method regarding parameter estimation and the calculation of the Fisher information matrix. As there is little inter-temporal correlation in the model, the benefit “a)” of the benchmark method is small. The Lotka-Volterra model shows an acceptable performance of the benchmark for small observation horizons. However, the MSS method performs better even here (Fig 13). For larger observation horizons (30 and 40) the difference becomes striking (last two rows of Fig 13). The benchmark is not able to reflect the location of the estimates anymore, while the MSS method still accurately describes the location of the estimates. The reason is that the benchmarks approximation is not valid anymore, while the less restrictive approximation of the MSS method still holds. This has been evaluated using a Kolmogorov-Smirnov test (Fig 14). Comparing this figure to S5 Fig showing the results for the Immigration-Death model, explains the benchmarks acceptable performance for the Immigration-Death model and the poor performance for the Lotka-Volterra model. Fig 13 also shows that the accuracy of the parameter estimation is less affected than the accuracy of the Fisher information, possibly due to the variance acting as a weighting factor for the parameter estimation. A very rough approximation of this weighting factor might have little influence (as indicated in [13]) on the parameter estimation but stronger influence on the Fisher information matrix, which describes how well the (parameter dependent) changes in the variance can be exploited.

While LNAs over the whole system’s horizon have been successfully used to model single-cell experiments [45, 46], the presented examples demonstrate that this is not generally the case and a less restrictive approximation such as the MSS is needed. The theoretical condition for the LNA approximation used in the MSS objective function are discussed in [13, 14] and reviewed in the supporting information S1 Text. Even the interval-wise LNA used in MSS can fail to hold (e.g. in case of only very few molecules present in the system), see [47] for details on theoretical and practical limitations of LNAs. Including higher order terms in the calculation of the covariance can improve the situation [48]. However, this has not been necessary for the presented models. A way to evaluate the validity of the LNA approximation was introduced using the Calcium model. The time steps for the creation of the pseudo data sets were varied and the impact on the entries of the *FI*_*MSS*_ was compared. This control helps to identify designs with an high amount of information and with an applicability of the MSS objective function for parameter estimation.

Work by [49] and [50] suggests and objective function using a LNA embedded in a Kalman filtering framework. This approach is similar to the MSS objective function as it also treats intervals between measurements separately and uses a LNA. However, the MSS method is more general as the state updating formulation (Eq 14) could also be straightforward extended to non Gaussian measurement noise. In case of Gaussian measurement noise the state updating formula (Eq 14) is equal to the state updating of [49, 50]. Differences can be found in the initialization of the variance/co-variance for each interval. The MSS initializes with 0 (as in Eq 12), [49, 50] use a Kalman filter recursion. This is an alternative to the updating procedure for MSS and might allow for a more precise description of the variance and co-variance. Therefore, it might be a promising objective function to be incorporated into an experimental design framework. However, this approach has not yet been used for calculating a Fisher Information matrix or experimental design.

Calculating exact Fisher information matrices is only possible in small example models such as the Immigration-Death model. However, the comparison of the MSS method to an exact method is an important part of a performance study. The results show that the accuracy of parameter estimation and of the calculation of the Fisher information matrix are comparable to the exact approach (MSS in Figs 3, 4 and 5, exact method in Figs 9, 7 and 8). This is an important message as it shows that the use of the interval-wise LNA approximation does not lead to a loss of accuracy for the Immigration-Death model.

The Fisher information is a parameter-dependent measure. Therefore, its power for experimental design depends on the knowledge of parameters based on professional expertise or previous experiments. If there is no such knowledge, robust experimental design [17, 18] or Bayesian experimental design [23] suggest strategies for a deterministic framework, which are applicable to the *FI*_*MSS*_.

It is possible to fix the random seed before the computation making the *FI*_*MSS*_ Fisher information deterministic. This is very advantageous for the optimization where the user can apply Bayesian techniques as well as global optimization or gradient based methods.

None of the computations in this article required the use of a computing cluster. One evaluation of the *FI*_*MSS*_ Fisher information matrix takes less than one minute on one kernel for the Immigration-Death model, approximately 20 minutes (LV1) to 120 minutes (LV2) on one kernel for the Lotka-Volterra model, and roughly 6 hours for the Calcium oscillation model on eight kernels. This demonstrates the applicability of the *FI*_*MSS*_ Fisher information matrix to realistic size models from a computational point of view.

## Supporting Information

S1 Text Supporting information text. (PDF)

S1 Fig Dependence of the accuracy of the *FI*_*MSS*_ entries on the number of pseudo data sets for the fully observed Lotka-Volterra model. Each panel shows one entry of the Fisher information matrix. Note that the *i* × *j* entry is identical to the *j* × *i* entry due to the symmetry of the matrix. The x-axis shows the number of pseudo data sets used for calculating the sample mean of Eq (7) shown as solid line. Gray color shows the area from sample mean plus / minus one standard deviation. As the width of the gray are is decreasing, the accuracy is increasing. Small values as *M* = 200 already give a good approximation.
(TIF)

S2 Fig Dependence of the accuracy of the *FI*_*MSS*_ entries on the number of pseudo data sets for the partially observed Lotka-Volterra model. Same setting as in S1 Fig.
(TIF)

S3 Fig Dependence of the accuracy of the *FI*_*MSS*_ entries on the number of pseudo data sets for the fully observed Calcium oscillation model. Same setting as in S1 Fig.
(PDF)

S4 Fig Dependence of the accuracy of the *FI*_*MSS*_ entries on the number of pseudo data sets for the partially observed Calcium oscillation model. Same setting as in S1 Fig.
(PDF)

S5 Fig Testing the approximation for different observations horizons. P-values for Kolmogorov-Smirnov tests whether the approximation is fulfilled for different observation horizons in the Immigration-Death model; left panel shows results for benchmark and right panel for MSS. Each color stands for one of the 50 data sets. Test is performed with the true parameter *θ* = (1, 0.1) and the scenario with the largest inter-sample distance, namely Δ*t* = 15.
(TIF)

S6 Fig D-criterion and E-criterion for the exact method for the Immigration-Death model with multiple evaluations of *FI*_*emp*,*ex*_. *FI*_*ex*_ Fisher information and *FI*_*emp*,*ex*_ are calculated for different inter-sample distances. The solid line is an interpolation of the values of *FI*_*ex*_ and the “x” denote the 10 values of *FI*_*emp*,*ex*_.
(TIF)

S7 Fig Parameter estimates and confidence ellipsoid from *FI*_*MSS*_ for Calcium model using the Gillespie algorithm to generate the pseudo data. The panels show the two dimensional projections of the 12-dimensional parameter space for the Δ*t* = 0.5 design. In each panel the black dots are the estimates from simulated data and the confidence ellipsoid of the Fisher information is marked red. “k” is used as an abbreviation for “thousand”.
(TIF)

S1 File Mathematica code of the method and all simulation studies. (TGZ)
