# Supplementary material for: Experimental Design for Stochastic Models of Nonlinear Signaling Pathways Using an Interval-Wise Linear Noise Approximation and State Estimation
Source: PLoS One. 2016 Sep 1;11(9):e0159902. doi: 10.1371/journal.pone.0159902 (PMC5008843; doi:10.1371/journal.pone.0159902)
Supplement: S1 Text — (PDF) [file pone.0159902.s001.pdf]

Supporting information text

Derivation of the MSS objective function

This supporting information explains how the MSS objective function is derived. It is a review of the respective section in [14].

Given observations $\mathscr{O}_0, \mathscr{O}_1, \ldots, \mathscr{O}_n$ at time points $t_0, t_1, \ldots, t_n$, the observation based likelihood function $L(\mathscr{O}, \theta)$ gives the probability to obtain the data $\mathscr{O}$ given a parameter θ. This likelihood function can be factorized into

$$L(\mathscr{O}, \theta) \ = \prod_{i=1}^{n} P(\mathscr{O}_i; \mathscr{O}_{i-1}, \ldots, \mathscr{O}_0, \theta), \tag{35}$$

where $P(\mathscr{O}_i; \mathscr{O}_{i-1}, \ldots, \mathscr{O}_0, \theta)$ is the probability to observe $\mathscr{O}_i$ given previous observations $\mathscr{O}_{i-1}, \ldots, \mathscr{O}_0$. This probability can be written as

$$P(\mathscr{O}_i; \mathscr{O}_{i-1}, \ldots, \mathscr{O}_0, \theta) = \sum_{\nu_i \in \Omega_i, \nu_{i-1} \in \Omega_{i-1}} P(\mathscr{O}_i; \nu_i) P(\nu_i; \nu_{i-1}, \theta) P(\nu_{i-1}; \mathscr{O}_{i-1}, \ldots, \mathscr{O}_0, \theta) \tag{36}$$

Measurement noise

The first factor describes the measurement noise: $P(\mathscr{O}_i; \nu_i)$ is the probability to measure $\mathscr{O}_i$ if being in state ν_i. As this is in most cases a Gaussian distribution, its calculation is computationally fast because it does neither involve stochastic simulations nor ODE solutions.

Transition probability

The second probability is the transition probability for a transition from to ν_{i-1} to ν_i. Its distribution is generally unknown. It could be calculated with the use of simulations and density estimation methods or the solution of a high dimensional chemical master equation system. Both are from computational point very time consuming. Therefore, [13, 14] suggest to approximate it with a normal distribution:

$$P(\nu_i; \nu_{i-1}, \theta) \approx p(\nu_i; \nu_{i-1}, \theta) = f\left(\nu_i | x(\Delta t; \theta, \nu_{i-1}), \Sigma(\Delta t; \theta, \nu_{i-1})\right), \tag{37}$$

where $f(y|\mu, \Sigma)$ is the probability density function of a multivariate normal distribution with mean μ and covariance Σ which is calculated by a linear noise approximation as in equation (12).

As the Gaussian distribution has a continuous support, the probability for $P(\mathscr{O}_i; \mathscr{O}_{i-1}, \ldots, \mathscr{O}_0, \theta)$ in equation (36) is calculated with an integral instead of the sum:

$$P(\mathscr{O}_i; \mathscr{O}_{i-1}, \ldots, \mathscr{O}_0, \theta) \approx \tag{38}$$

$$\int_{\nu_i \in \Lambda_i, \nu_{i-1} \in \Lambda_{i-1}} P(\mathscr{O}_i; \nu_i) p(\nu_i; \nu_{i-1}, \theta) P(\nu_{i-1}; \mathscr{O}_{i-1}, \ldots, \mathscr{O}_0, \theta) d\nu_i d\nu_{i-1} \tag{39}$$

where Λ_i stands for the state space at time point t_i. In many cases the state space will be constant over time, hence $\Lambda = \Lambda_1 = \Lambda_2, \ldots = \Lambda_n$.

State estimation

The third probability $P(\nu_{i-1}; \mathcal{O}_{i-1}, \ldots, \mathcal{O}_0, \theta)$ is the probability to be in a state ν_{i-1} given the observations $\mathcal{O}_{i-1}, \ldots, \mathcal{O}_0$. This probability is challenging from a computational point of view due to two reasons: first, its evaluation needs computational expensive formulas from hidden Markov processes (equation 1 in [51]). Second, even once it is evaluated for every point ν_{i-1} in the state space Λ_{i-1}, the transition probability $p(\nu_i; \nu_{i-1}, \theta)$ has to be calculated for any of these points. This is, even with the approximation, computationally challenging (see also comment in discussion).

Therefore, a state updating is used instead of the full probability distribution $P(\nu_{i-1}; \mathcal{O}_{i-1}, \ldots, \mathcal{O}_0, \theta)$ to estimate the state ν_{i-1} at time point t_{i-1}: Given a state estimate $\hat{\nu}_{i-1}$ at time t_{i-1}, the probability to see the observation $\mathcal{O}_i$ at time t_i is the product of the probability to move from state $\hat{\nu}_{i-1}$ to a state ν_i and the probability to see $\mathcal{O}_i$ if the state is ν_i. A state estimate $\hat{\nu}_i$ can be defined as the state that leads to the highest probability to observe $\mathcal{O}_i$:

$$\hat{\nu}_i = \text{argmax}_{\nu_i} \left(f\left(\nu_i | x(\Delta t; \theta, \hat{\nu}_{i-1}), \Sigma(\Delta t; \theta, \hat{\nu}_{i-1})\right) \cdot f\left(\mathcal{O}_i | \nu_i^{\text{obs}}, \Sigma^{\text{meas}}\right)\right) \tag{40}$$

for $i = 1, \ldots, n-1$ and x as in equation (2) and Σ as in equation (12). The initial state $\hat{\nu}_0$ is included into the optimization vector. This is a quadratic optimization problem with an analytical solution and therefore computationally very fast.

By definition the state estimate incorporates information available from the previous observations. The state estimate $\hat{\nu}_{i-1}$ is then used to evaluate $P(\mathcal{O}_i, \mathcal{O}_{i-1}, \ldots)$ as

$$P(\mathcal{O}_i; \mathcal{O}_{i-1}, \ldots, \mathcal{O}_0, \theta) \approx \int_{\nu_i \in \Lambda_i} P(\mathcal{O}_i; \nu_i) p(\nu_i; \hat{\nu}_{i-1}, \theta) d\nu_i \tag{41}$$

The state estimation procedure can be considered as a 1-point evaluation of the integral or an approximation of the distribution with a point distribution. Although this seems to be a rough approximation, extensive simulation studies [14] have shown that this allows to estimate states and parameters. When considering different models, there are two options to check whether the approximation is appropriate: first, stochastically simulating time courses, applying this approach to them, and checking how well the states are estimated. Second, using updates formulas for $P(\nu_{i-1}; \mathcal{O}_{i-1}, \ldots, \mathcal{O}_0, \theta)$ (such as equation 1 in [51]) to evaluate the term in equation (36) and comparing the result to equation (41). The wide use of Kalman-filtering techniques [52] shows that the use of state estimates allows for an successful analysis.

The MSS objective function

Taking the logarithm of equation (35), inserting the approximation of equation (41) and considering Gaussian measurement error, leads to the MSS objective function as displayed in equation (15).

Settings for the parameter estimations

Immigration-Death model:

The parameter estimation for the Immigration-Death model was carried out by optimizing the MSS objective function (equation (15)). The optimization was performed with the FindMinimum algorithm in the software Mathematica 9 [53]. The search was

initialized with the true parameter and terminated with at maximum 500 iterations. Only for the parameter estimations with the benchmark method, the version 10.4 [54] was used as the version 9.0 seemed to be much slower here.

Lotka-Volterra model:

The parameter estimation for the Lotka-Volterra model was also carried out with the FindMinimum algorithm in the software Mathematica 9. The search was initialized with the true parameter $\theta^{(0)}$ and $(1.1, 1.1, 1.1, 0.9)\theta^{(0)}$ as a second start value.

Calcium oscillation model:

The parameter estimation for the fully observed Calcium oscillation model was carried out using a Particle Swarm [55] algorithm implemented in Mathematica [53]. The search domain of the Particle Swarm was

$$[50, 400] \times [2.75, 3.25] \times [1.4, 1.75] \times [.01, 1000] \times [3, 7] \times [1, 5000] \times [1., 1.4] \quad (42)$$
$$\times [27500, 40000] \times [20000, 40000] \times [10, 20] \times [130000, 170000] \times [100, 200] \quad (43)$$

with 125 iterations and 25 particles per iteration. The partially observable Calcium model had a search domain of

$$[1, 400] \times [2.7, 3.75] \times [1, 2.5] \times [0.1, 1000] \times [0.1, 7] \times [0.1, 10000] \times [0.25, 2.5] \quad (44)$$
$$\times [10000, 40000] \times [10000, 75000] \times [5, 500] \times [100000, 300000] \times [10, 300] \quad (45)$$
$$\times [0.001, 1000] \times [0.01, 1000] \quad (46)$$

with 300 iterations and 150 particles per iteration.

Calculation of the Fisher information for the exact approach for the Immigration-Death model

The Fisher information FI_{ex} (equation 30) is derived as follows (see also [56]) with

$$B_i = \frac{\partial}{\partial \vartheta_j} log(P_{ex}(\nu_i, t_i - t_{i-1}; \nu_{i-1}, \theta)) \frac{\partial}{\partial \vartheta_k} log(P_{ex}(\nu_i, t_i - t_{i-1}; \nu_{i-1}, \theta)) \quad (47)$$

as an abbreviation

$$\begin{aligned} FI_{ex}(T,\vartheta)_{jk} &= E_{\mathscr{O}}\left[\frac{\partial}{\partial\vartheta_j}\, logL_{ex}(\mathscr{O},\vartheta)\ \ \frac{\partial}{\partial\vartheta_k}\, logL_{ex}(\mathscr{O},\vartheta)\right] && (48)\\ &= \sum_{\nu_1,\ldots,\nu_n}\left(\sum_{i=1}^{n}\frac{\partial}{\partial\vartheta_j}\, log(P_{ex}(\nu_i,t_i-t_{i-1};\nu_{i-1},\theta))\right. && (49)\\ &\quad \left.\frac{\partial}{\partial\vartheta_k}\, log(P_{ex}(\nu_i,t_i-t_{i-1};\nu_{i-1},\theta))\right)P(\nu_1,\ldots,\nu_n) && (50)\\ &= \sum_{\nu_1}\cdots\sum_{\nu_n}\left(\sum_{i=1}^{n}B_i\right)P(\nu_n|\nu_{n-1})\ldots P(\nu_1,\nu_0) && (51)\\ &= \sum_{i=1}^{n}\sum_{\nu_1}\cdots\sum_{\nu_n}B_iP(\nu_n|\nu_{n-1})\ldots P(\nu_1,\nu_0) && (52)\\ &= \sum_{i=1}^{n}\sum_{\nu_i}\sum_{\nu_{i-1}}B_i\sum_{\nu_n}\cdots\sum_{\nu_{i+1}}\sum_{\nu_{i-2}}\cdots\sum_{\nu_0}P(\nu_i|\nu_{i-1}) && (53)\\ &= \sum_{i=1}^{n}\sum_{\nu_i}\sum_{\nu_{i-1}}B_iP(\nu_i|\nu_{i-1})P(\nu_{i-1}|\nu_0) && (54) \end{aligned}$$

In the second equality the Markov property is used, the third and fourth change the summation in a suitable order and the fifth uses the following relations:

$$\begin{aligned} \sum_{\nu_j}P(\nu_j|\nu_{j-1}) &= 1 \text{ for } j=i+1,\ldots,n,\\ \sum_{\nu_j}P(\nu_{j+1}|\nu_j)\ P(\nu_j|\nu_{j-1}) &= P(\nu_{j+1}|\nu_{j-1}) \text{ for } j=1,\ldots,i-2. \end{aligned} \quad (55)$$

References

1. Raj A, van Oudenaarden A. Single-Molecule Approaches to Stochastic Gene Expression. Annu Rev Biophys. 2009;38:255–270.

2. Gillespie DT. A General Method for Numerically Simulating the Stochastic Time Evolution of coupled Chemical Reactions. Journal of Computational Physics. 1976;22 (4):403–434.

3. Pahle J. Biochemical simulations: stochastic, approximate stochastic and hybrid approaches. Briefings in Bioinformatics. 2009;10 (1):53–64.

4. Andreychenko A, Mikeev L, Spieler D, Wolf V. Approximate maximum likelihood estimation for stochastic chemical kinetics. EURASIP Journal on Bioinformatics and Systems Biology. 2012;9.

5. Gillespie CS. Moment-closure approximations for mass-action models. IET Systems Biology. 2009;3 (1):52–58.

6. Gillespie CS, Golightly A. Bayesian inference for generalized stochastic population growth models with application to aphids. Applied Statistics. 2010;59 (2):341–357.

7. Hasenauer J, Wolf V, Kazeroonian A, Theis FJ. Method of conditional moments (MCM) for the Chemical Master Equation; A unified framework for the method of moments and hybrid stochastic-deterministic models. J Math Biol. 2013;DOI 10.1007/s00285-013-0711-5.

8. Mikeev L, Wolf V. Parameter Estimation for Stochastic Hybrid Models of Biochemical Reaction Networks. HSCC 12, Beijing. 2012;.

9. Boys RJ, Wilkinson DJ, Kirkwood TBL. Bayesian inference for a discretely observed stochastic kinetic model. StatComput. 2008;18:125–135.

10. Wang Y, Christley S, Mjolsness E, Xie X. Parameter inference for discretely observed stochastic kinetic models using stochastic gradient descent. BMC Systems Biology. 2010;4:99.

11. Komorowski M, Costa MJ, Rand DA, Stumpf MPH. Sensitivity, robustness, and identifiability in stochastic chemical kinetics models. PNAS. 2011;108:21:8645–8650.

12. Zimmer C, Sahle S. Parameter Estimation for Stochastic Models of Biochemical Reactions. Journal of Computer Science & Systems Biology. 2012;6:011–021.

13. Zimmer C, Sahle S. Deterministic inference for stochastic systems using multiple shooting and a linear noise approximation for the transition probabilities. IET Systems Biology. 2015;9:181 – 192. doi:doi: 10.1049/iet-syb.2014.0020.

14. Zimmer C. Reconstructing the hidden states in time course data of stochastic models. Mathematical BioSciences. 2015;269:117 – 129.

15. Körkel S, Kostina E. Numerical Methods for Nonlinear Experimental Design. In: Bock HG, Kostina E, Phu HX, Rannacher R, editors. Modelling, Simulation and Optimization of Complex Processes, Proceedings of the International Conference on High Performance Scientific Computing. Hanoi, Vietnam: Springer; 2004. p. 255–272.

16. Faller D, Klingmüller U, Timmer J. Simulation Methods for Optimal Experimental Design in Systems Biology. SIMULATION. 2003;79:717–725.

17. Bock HG, Körkel S, Kostina E, Schlöder JP. Robustness Aspects in Parameter Estimation, Optimal Design of Experiments and Optimal Control. In: Reactive Flows, Diffusion and Transport. From Experiments via Mathematical Modeling to Numerical Simulation and Optimization Final Report of SFB (Collaborative Research Center) 359. Jäger, W. and Rannacher, R. and Warnatz, J.; 2007. p. 117–146.

18. Körkel S, Kostina E, Bock HG, Schlöder JP. Numerical Methods for Optimal Control Problems in Design of Robust Optimal Experiments for Nonlinear Dynamic Processes. Optimization Methods and Software. 2004;19:327–338.

19. Lehmann EL, Casella G. Theory of Point Estimation. Springer; 1998.

20. Fedorov VV, Hackl P. Model-Oriented Design of Experiments; 1997.

21. Bauer I, Bock HG, Köerkel S, Schlöder JP. Numerical Methods for Optimum Experimental Design in DAE systems. Journal of Computational and Applied Mathematics. 2000;120:1–25.

22. Kreutz C, Timmer J. Systems biology: experimental design. FEBS Journal. 2009;276:923–942.

23. Chaloner K, Verdinelli I. Bayesian Experimental Design: A Review. Statistical Science. 1995;10:273–304.

24. Steiert B, Raue A, Timmer J, Kreutz C. Experimental Design for Parameter Estimation of Gene Regulatory Networks. PlosONE. 2012;7:e40052.

25. Pagendam DE. Experimental Design and Inference for Population Models. PhD thesis, University of Queensland. 2010;.

26. Ruess J, Milias-Argeitis A, Lygeros J. Designing experiments to understand the variability in biochemical reaction networks. Journal of the Royal Society Interface. 2013;10.

27. Ruess J, Parise F, Milias-Argeitis A, Khammash M, Lygeros J. Iterative experiment design guides the characterization of a light-inducible gene expression circuit. PNAS. 2015;112:8148 – 8153.

28. Nandy P, Unger M, Zechner C, Koeppl H. Optimal Perturbations for the Identification of Stochastic Reaction Dynamics. IFAC; 2012. p. 686 – 691.

29. Berridge MJ, Bootman MD, Lipp P. Calcium - a life and death signal. Nature, news and views feature. 1998;395 (6703):645–648.

30. Kurtz TG. The Relationship between Stochastic and Deterministic Models for Chemical Reactions. The Journal of Chemical Physics. 1972;57(7):2976–2978.

31. Wilkinson DJ. Stochastic Modelling for Systems Biology. Boca Raton: Chapman & Hall/CRC, Mathematical and Computational Biology Series; 2006.

32. Wu S, Fu J, Cao Y, Petzold L. Michaelis-Menten speeds up tau-leaping under a wide range of conditions. The Journal of Chemical Physics. 2011;134 (13):134112.

33. Wilkinson DJ. Stochastic modeling for quantitative description of heterogeneous biological systems. Nature Reviews Genetics. 2009;10 (2):122–133.

34. Hoops S, Sahle S, Gauges R, Lee C, Pahle J, Simus N, et al. COPASI - a COmplex PAthway SImulator. Bioinformatics. 2006;22 (24):3067–3074.

35. Gardner TS, Cantor CR, Collins JJ. Construction of a genetic toggle switch in Escherichia coli. Letters to Nature. 2000;403:339–342.

36. Kummer U, Krajnc B, Pahle J, Green AK, Dixon CJ, Marhl M. Transition from Stochastic to Deterministic Behavior in Calcium Oscillations. Biophysical Journal. 2005;89:1603–1611.

37. Zimmer C, Sahle S, Pahle J. Exploiting intrinsic fluctuations to identify model parameters. IET Systems Biology. 2015;9:64–73.

38. Porat B, Friedlander B. Computation of the exact information matrix of Gaussian time series with stationary random components. IEEE T Acoust Speech. 1986;34:118 – 130.

39. Munsky B, Neuert G, van Oudenaarden A. Using Gene Expression Noise to Understand Gene Regulation. Science. 2012;336:183–187.

40. Munsky B, Trinh B, Khammash M. Listening to the noise: random fluctuations reveal gene network parameters. Molecular Systems Biology. 2009;5 (318).

41. Piaggio HTH. An elementary treatise on differential equations and their applications. London: Bell & Hyman; 1982.

42. Fröhlich F, Theis FJ, Hasenauer J. Uncertainty Analysis for Non-identifiable Dynamical Systems: Profile Likelihoods, Bootstrapping and More. In: Computational Methods in Systems Biology;.

43. Raue A, Kreutz C, Maiwald T, Bachmann J, Schilling M, Klingmüller U, et al. Structural and practical identifiability analysis of partially observed dynamical models by exploiting the profile likelihood. Bioinformatics;25 (15).

44. Grima R. A study of the accuracy of moment-closure approximations for stochastic chemical kinetics. J Chem Phys. 2015;136. doi:10.1063/1.370284.

45. Pahle J, Challenger JD, Mendes P, McKane AJ. Biochemical fluctuations, optimisation and the linear noise approximation. BMC Systems Biology. 2012;6.

46. Elf J, Ehrenberg M. Fast Evaluation of Fluctuations in Biochemical Networks With the Linear Noise Approximation. Genome Research. 2003;13:2475 − 2484.

47. Grima R. An effective rate equation approach to reaction kinetics in small volumes: Theory and application to biochemical reactions in nonequilibrium steady-state conditions. The Journal of Chemical Physics. 2010;133:035101.

48. Thomas P, Matuschek H, Grima R. How reliable is the linear noise approximation of gene regulatory networks? BMC Genomics. 2013;14.

49. Finkenstädt B, Woodcock DJ, Komorowski M, Harper C, Davis JRE, White MRH, et al. Quantifying intrinsic and extrinsic noise in gene transcription using the linear noise approximation: an application to single cell data. The Annals of Applied Statistics. 2013;7:1960–1982. doi:10.1214/13-AOAS669.

50. Fearnhead P, Giagos V, Sherlock C. Inference for Reaction Networks Using the Linear Noise Approximation. Biometrics. 2014;70:457–466. doi:10.111/biom.12152.

51. Smallwood RD, Sondik EJ. The Optimal Control of Partially Observable Markov Processes Over a Finite Time Horizon. Operations Research. 1973;21:1071–1088.

52. Evensen G. The Ensemble Kalman Filter: theoretical formulation and practical implementation. Ocean Dynamics. 2003;53:343–367.

53. Mathematica, Version 9.0. Wolfram Research, Inc. 2012;Champaign, IL.

54. Mathematica, Version 10.4. Wolfram Research, Inc. 2015;Champaign, IL.

55. Kennedy J, Eberhart R. Particle Swarm Optimization. Proceedings of the Fourth IEEE International conference on Neural Networks, Perth, Australia. 1995;4 (6):1942–1948.

56. Zimmer C. Parameter estimation for stochastic models of biochemical reactions. PhD thesis, University of Heidelberg. 2012;.
