# Supplementary figures and images for: Experimental Design for Stochastic Models of Nonlinear Signaling Pathways Using an Interval-Wise Linear Noise Approximation and State Estimation
Source: PLoS One. 2016 Sep 1;11(9):e0159902. doi: 10.1371/journal.pone.0159902 (PMC5008843; doi:10.1371/journal.pone.0159902)

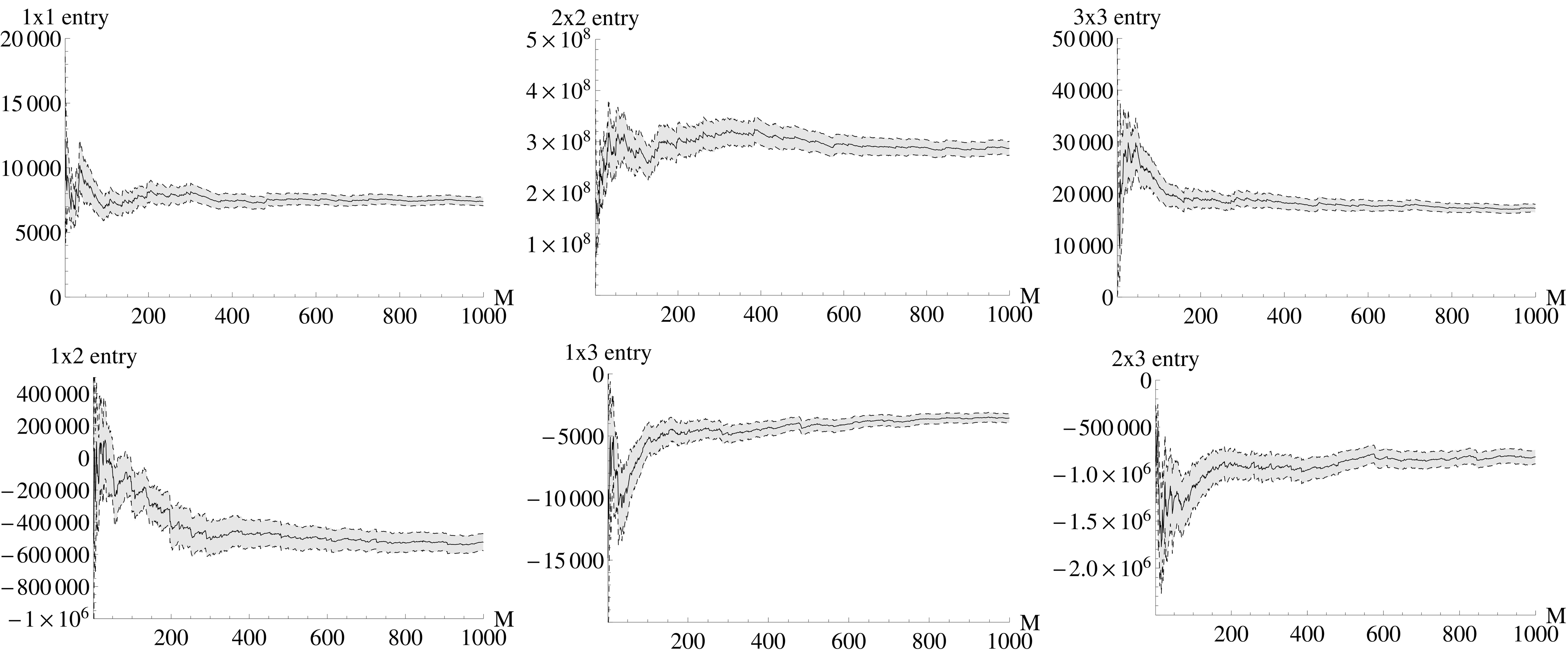

Supplement: S1 Fig — Each panel shows one entry of the Fisher information matrix. Note that the i × j entry is identical to the j × i entry due to the symmetry of the matrix. The x-axis shows the number of pseudo data sets used for calculating the sample mean of Eq (7) shown as solid line. Gray color shows the area from sample mean plus / minus one standard deviation. As the width of the gray are is decreasing, the accuracy is increasing. Small values as M = 200 already give a good approximation. (TIF) [file pone.0159902.s002.tif]

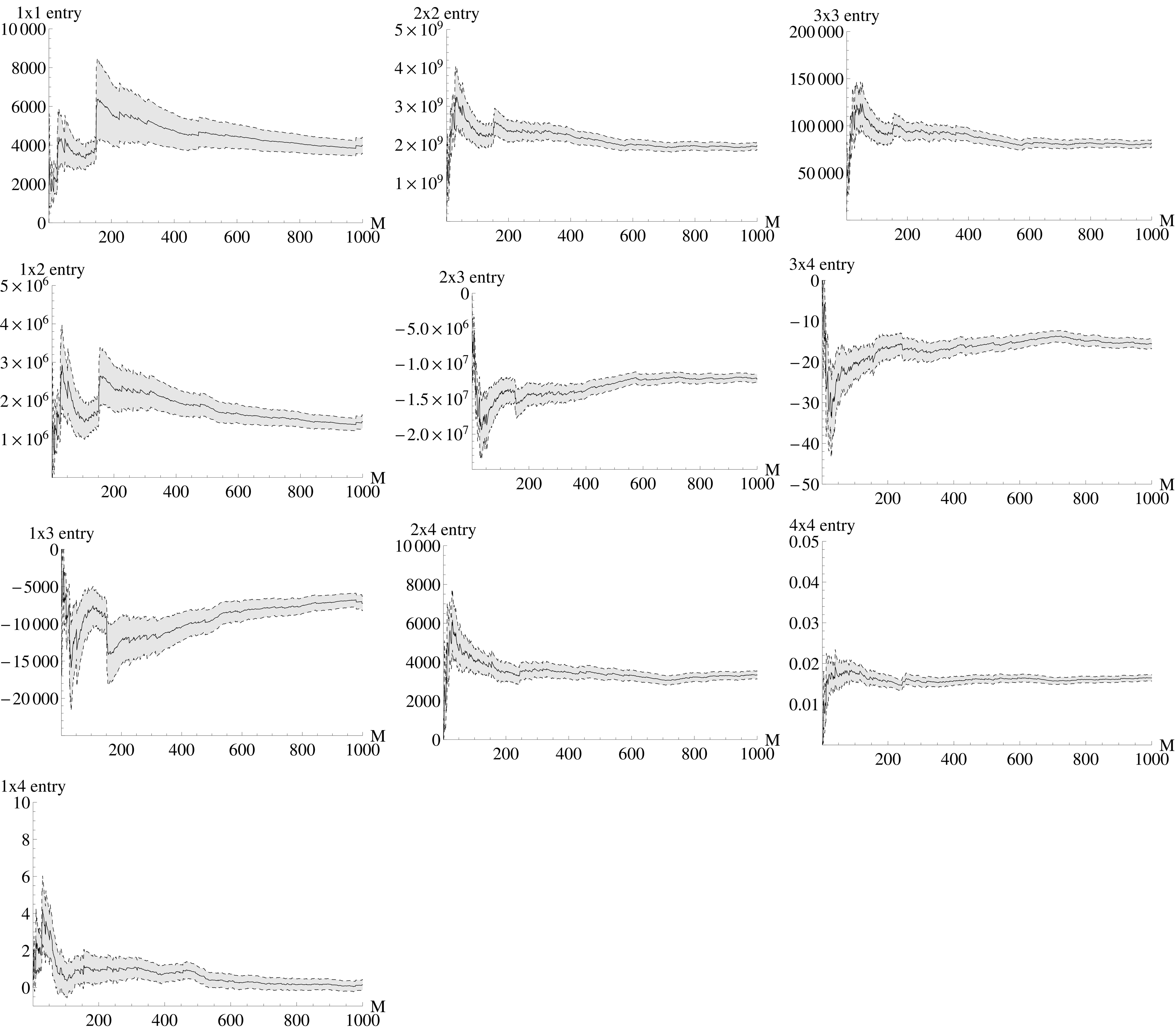

Supplement: S2 Fig — Same setting as in S1 Fig. (TIF) [file pone.0159902.s003.tif]

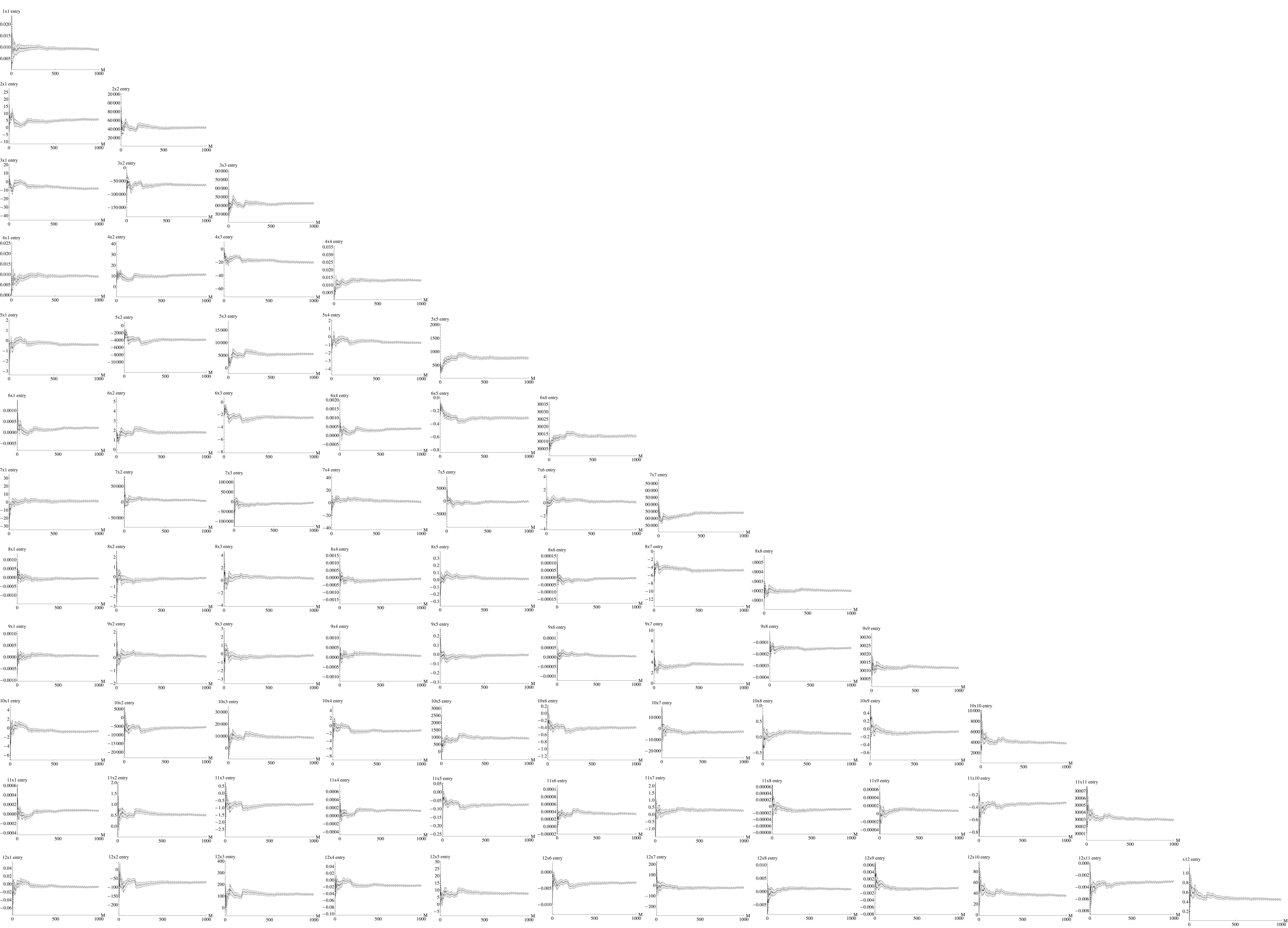

Supplement: S3 Fig — Same setting as in S1 Fig. (PDF) [file pone.0159902.s004.pdf]

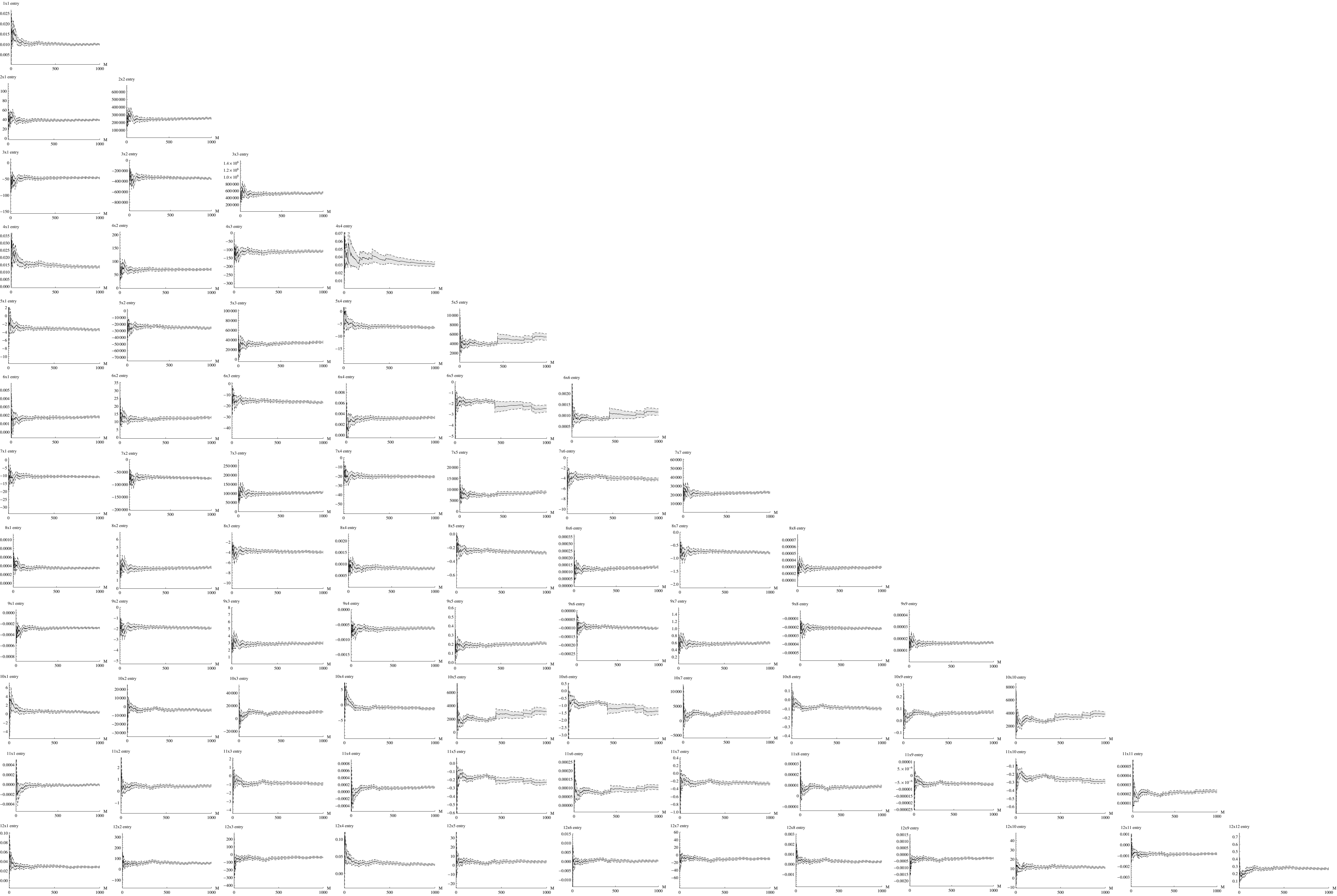

Supplement: S4 Fig — Same setting as in S1 Fig. (PDF) [file pone.0159902.s005.pdf]

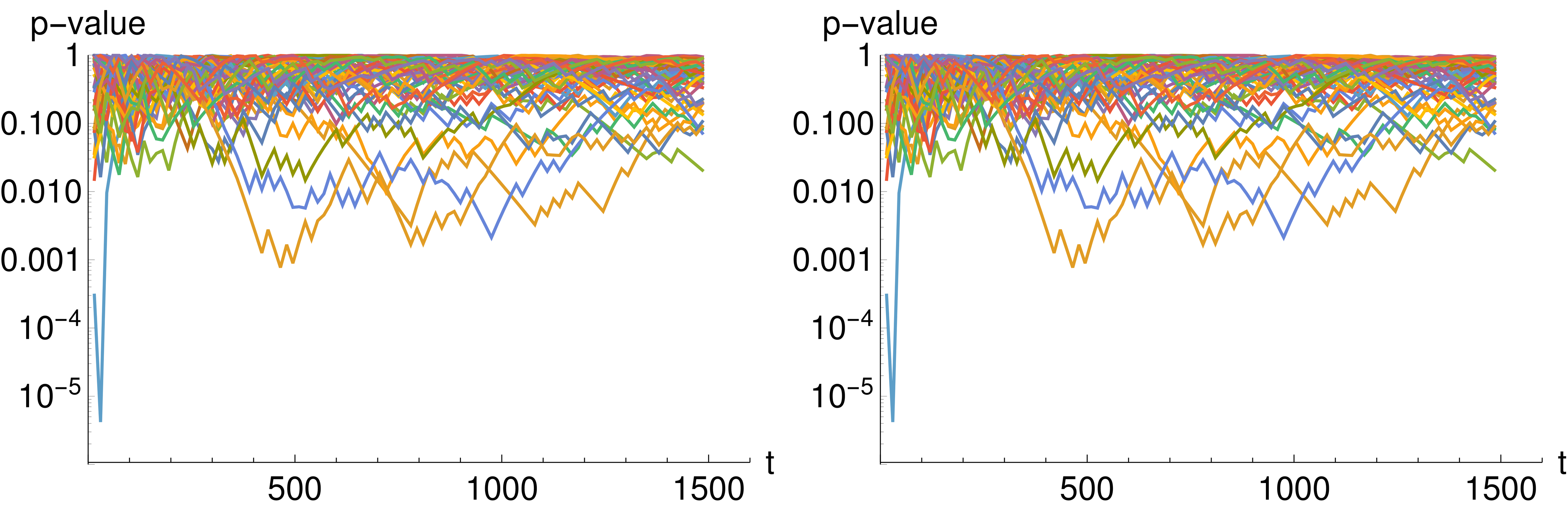

Supplement: S5 Fig — P-values for Kolmogorov-Smirnov tests whether the approximation is fulfilled for different observation horizons in the Immigration-Death model; left panel shows results for benchmark and right panel for MSS. Each color stands for one of the 50 data sets. Test is performed with the true parameter θ = (1, 0.1) and the scenario with the largest inter-sample distance, namely Δt = 15. (TIF) [file pone.0159902.s006.tif]

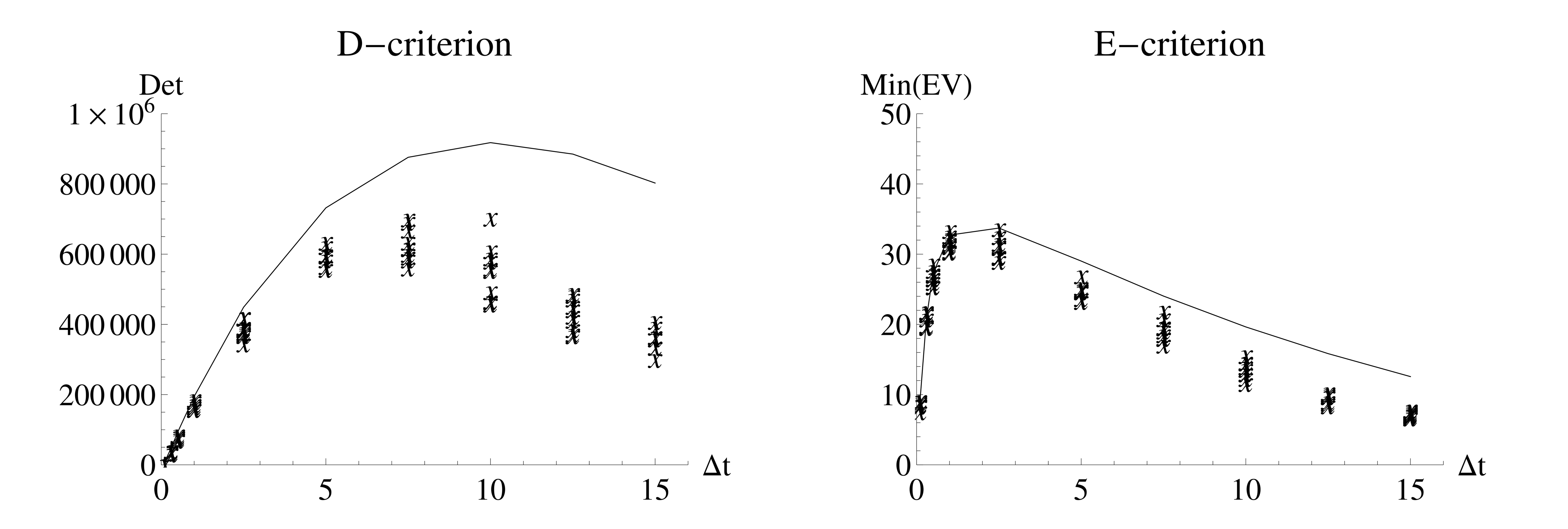

Supplement: S6 Fig — FIex Fisher information and FIemp,ex are calculated for different inter-sample distances. The solid line is an interpolation of the values of FIex and the “x” denote the 10 values of FIemp,ex. (TIF) [file pone.0159902.s007.tif]

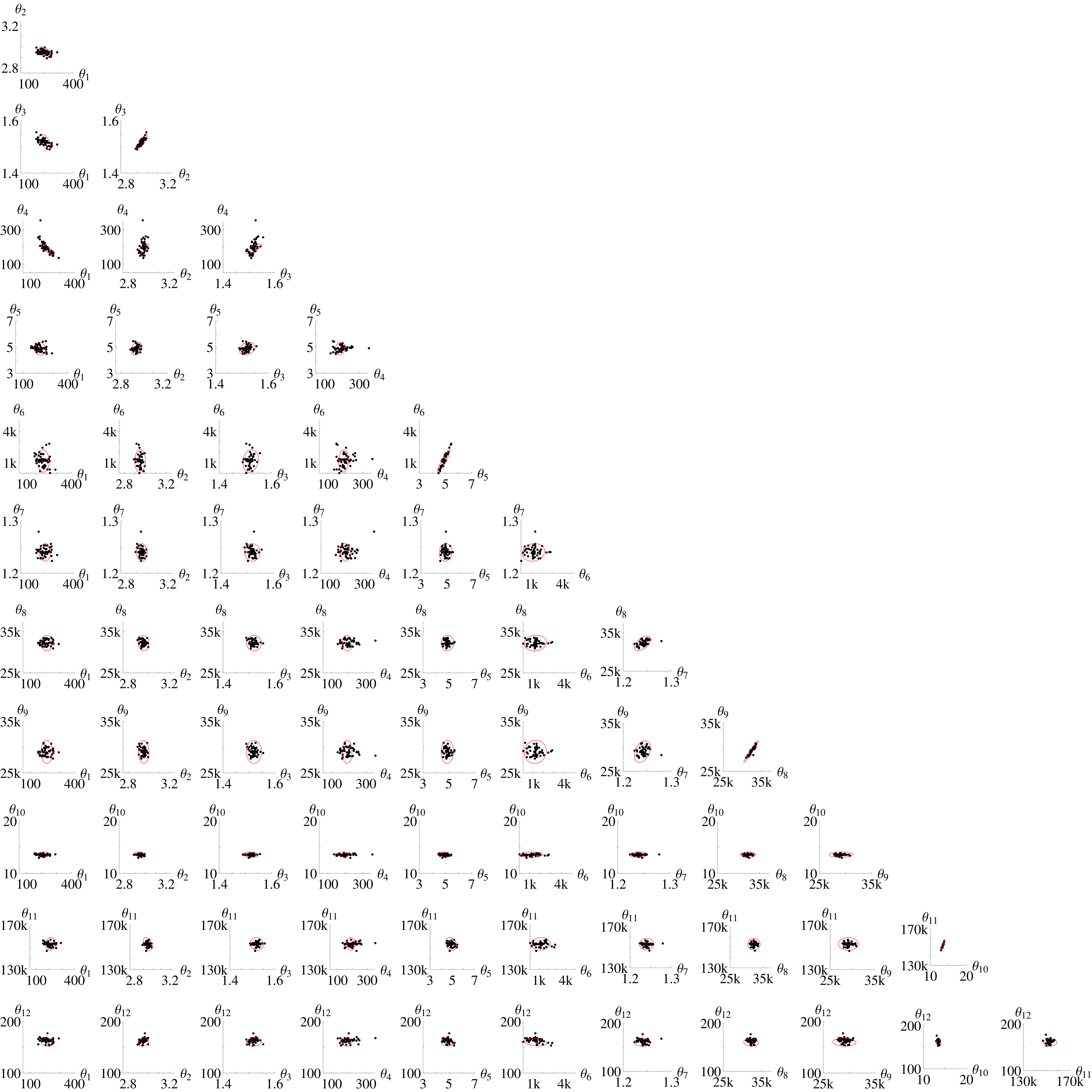

Supplement: S7 Fig — The panels show the two dimensional projections of the 12-dimensional parameter space for the Δt = 0.5 design. In each panel the black dots are the estimates from simulated data and the confidence ellipsoid of the Fisher information is marked red. “k” is used as an abbreviation for “thousand”. (TIF) [file pone.0159902.s008.tif]

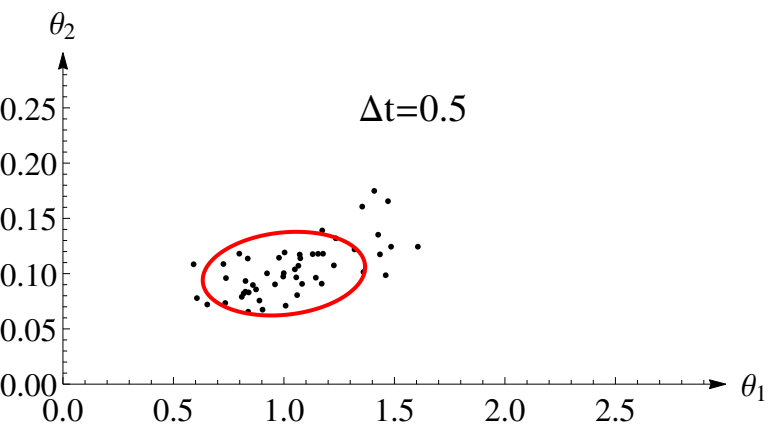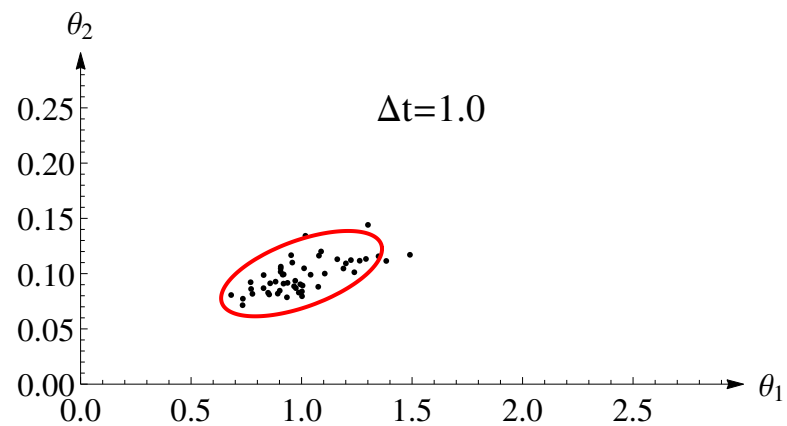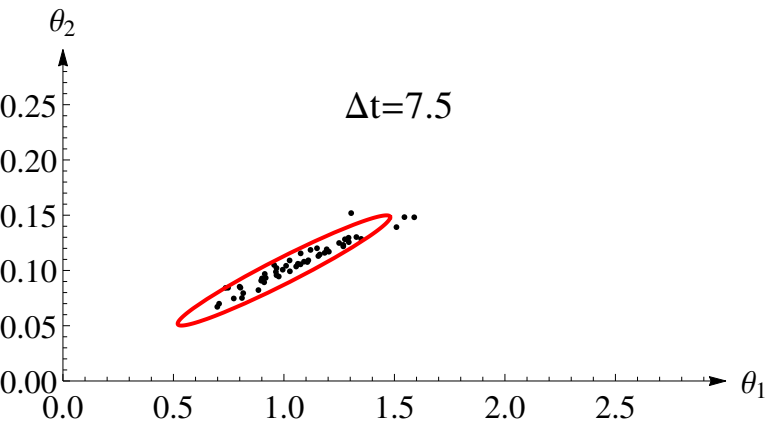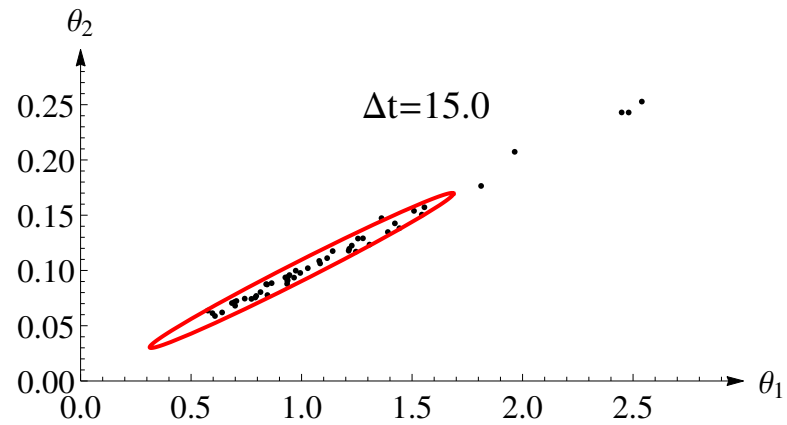

Supplement: S1 File — (TGZ) [file pone.0159902.s009.tgz › Online_SI/graphics/ID2dplots.pdf]

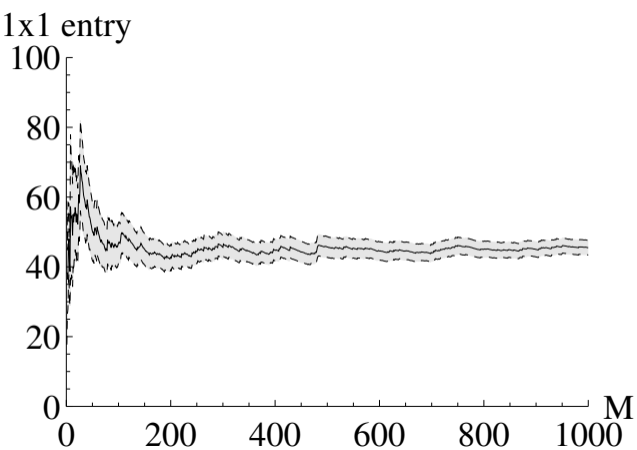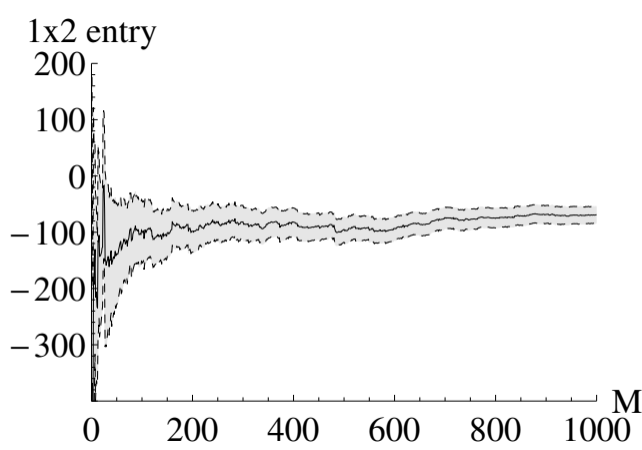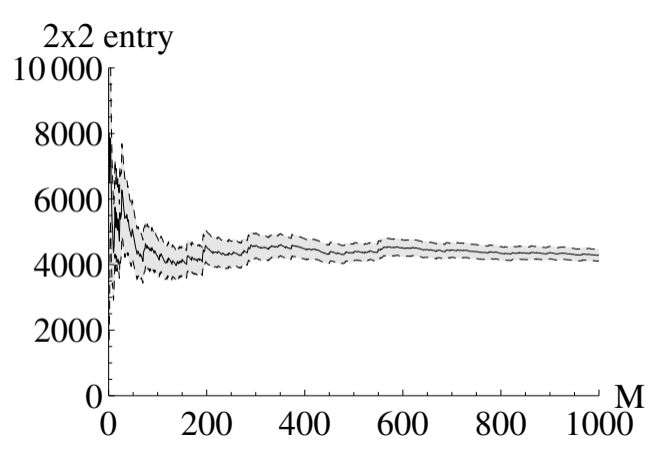

Supplement: S1 File — (TGZ) [file pone.0159902.s009.tgz › Online_SI/graphics/IDconv1.pdf]

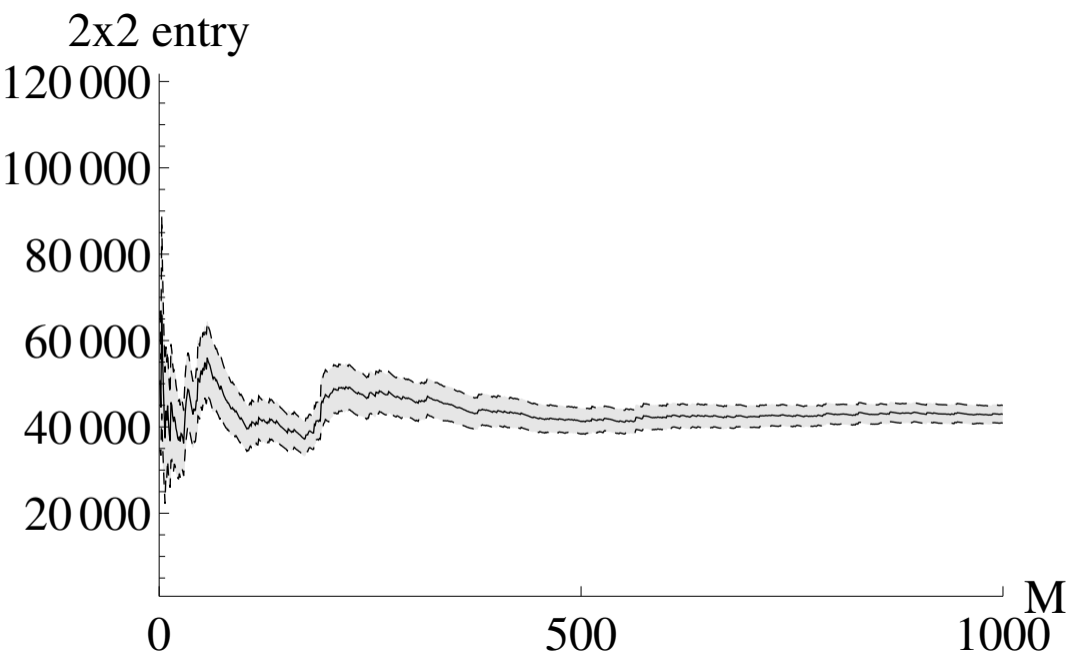

Supplement: S1 File — (TGZ) [file pone.0159902.s009.tgz › Online_SI/graphics/Caconv22.pdf]

A: fully observed

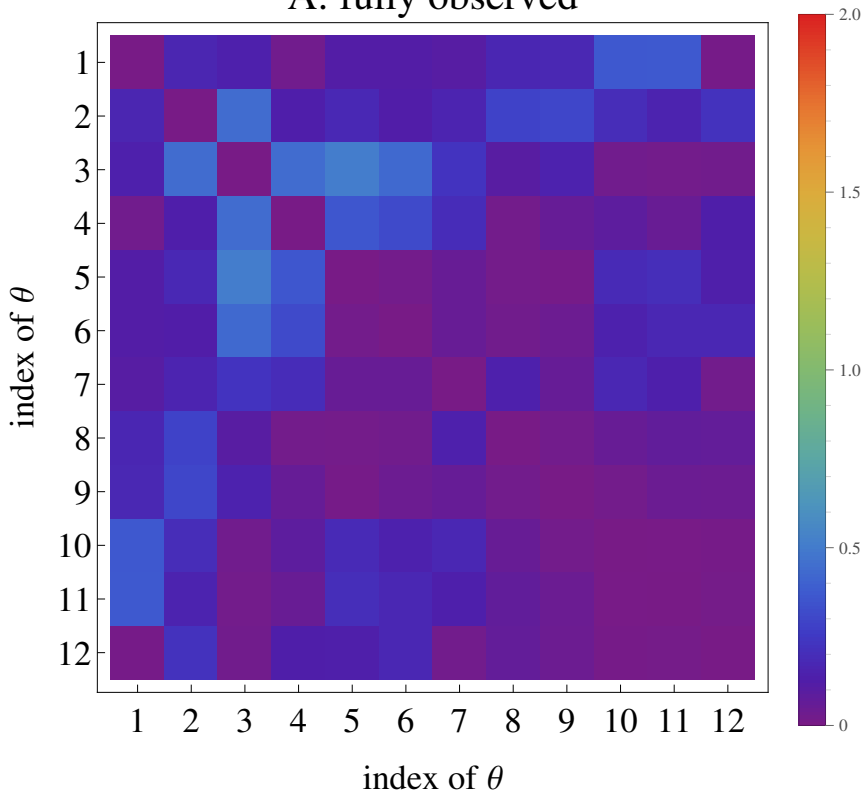

Supplement: S1 File — (TGZ) [file pone.0159902.s009.tgz › Online_SI/graphics/Cacorr.pdf]

ARSE  $\theta_1$ 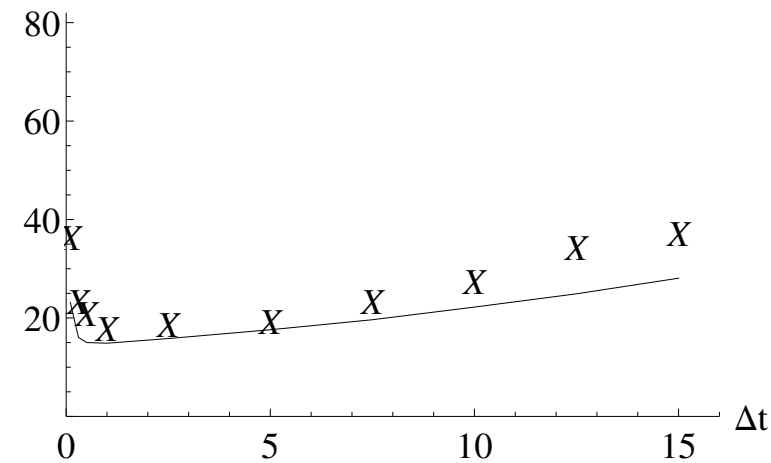ARSE  $\theta_2$ 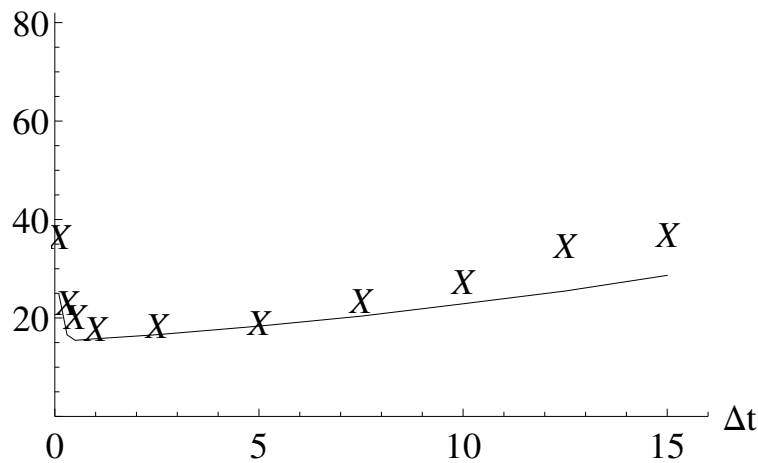

Supplement: S1 File — (TGZ) [file pone.0159902.s009.tgz › Online_SI/graphics/IDarse.pdf]

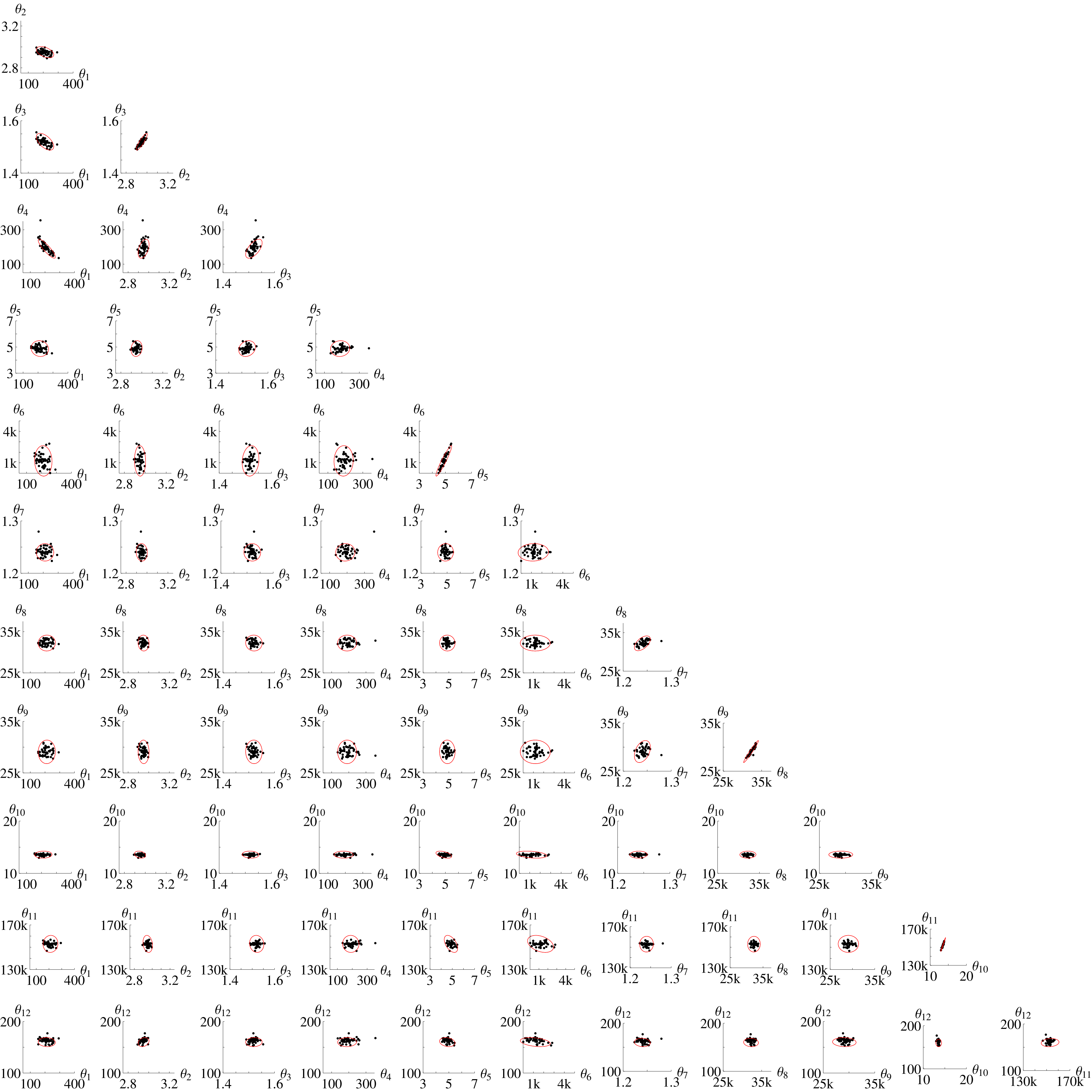

Supplement: S1 File — (TGZ) [file pone.0159902.s009.tgz › Online_SI/graphics/Ca2ds.pdf]

D-criterion

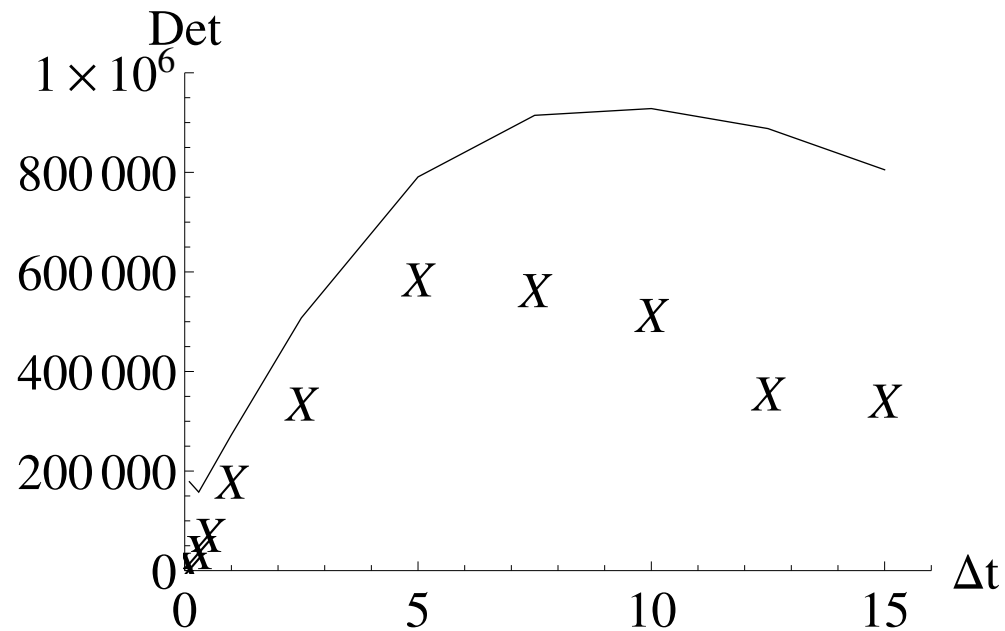

E-criterion

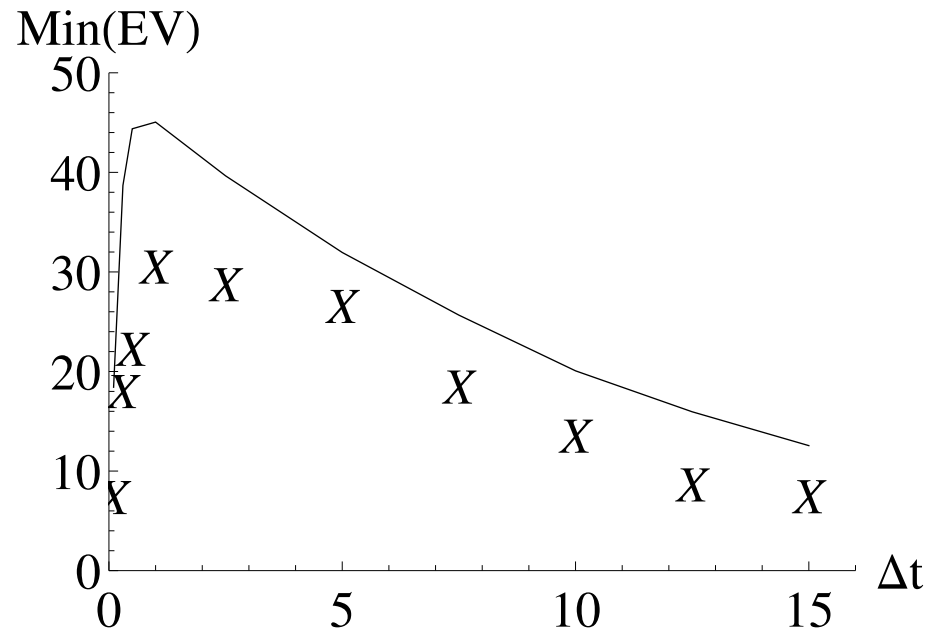

Supplement: S1 File — (TGZ) [file pone.0159902.s009.tgz › Online_SI/graphics/IDdesigns.pdf]

deterministic

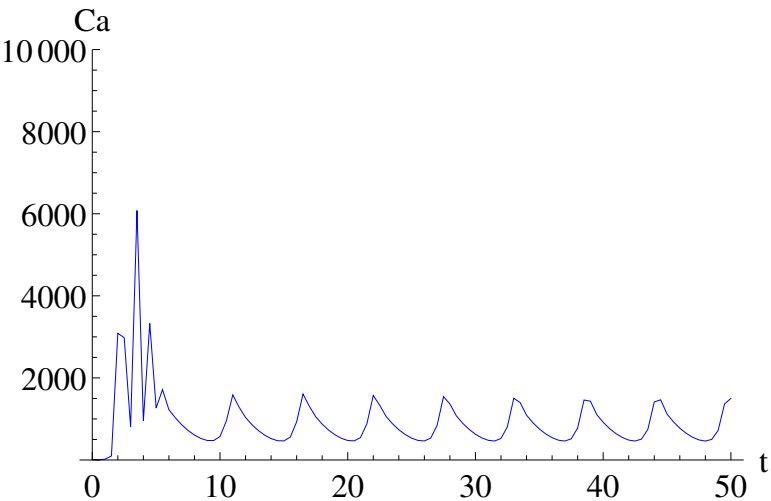

stochastic

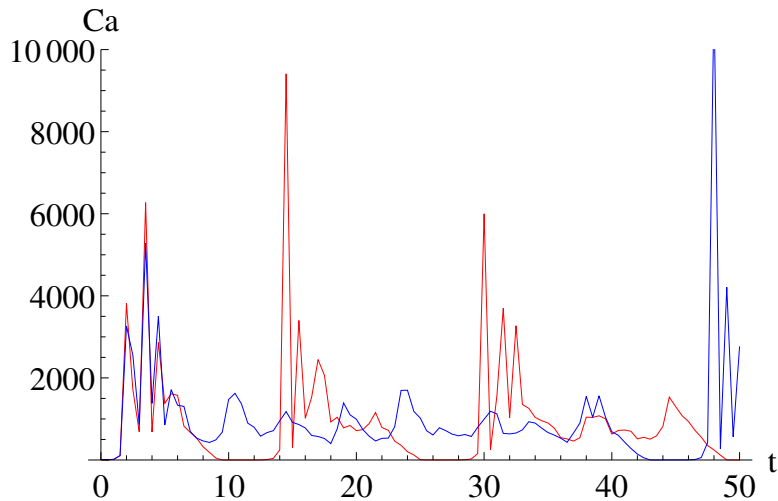

Supplement: S1 File — (TGZ) [file pone.0159902.s009.tgz › Online_SI/graphics/Cadetstoch.pdf]

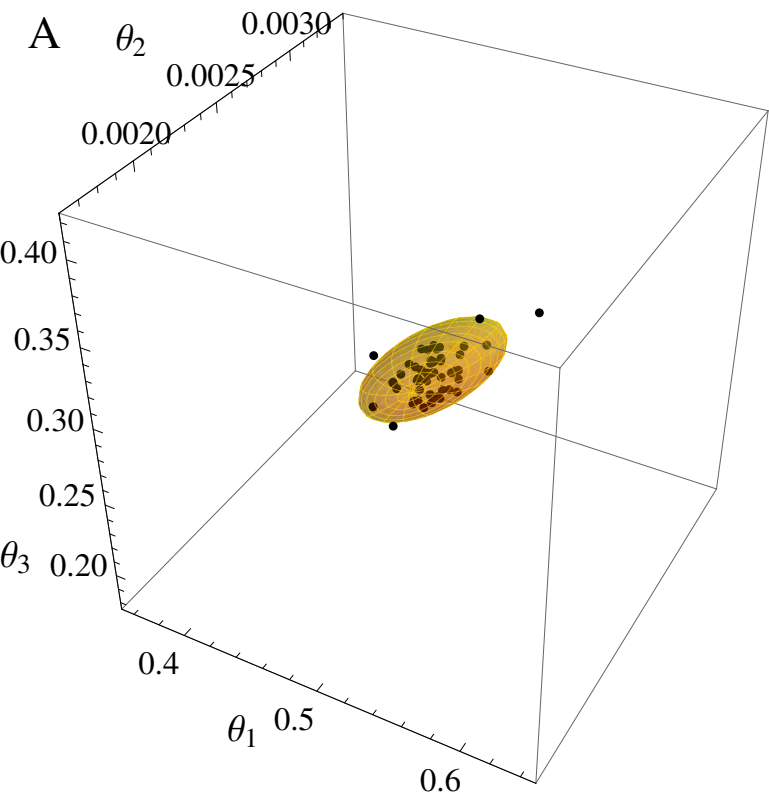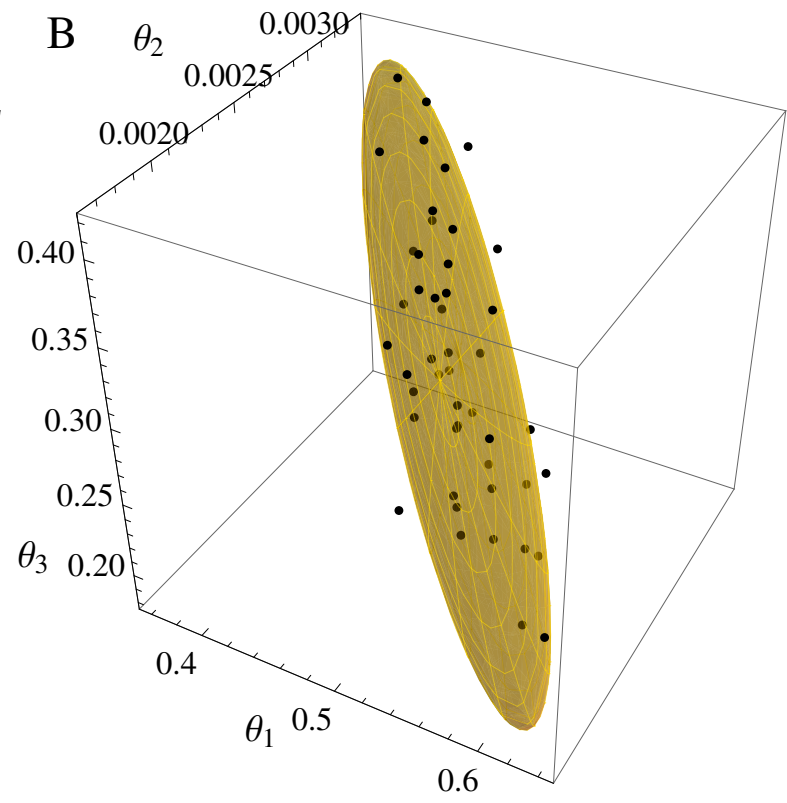

Supplement: S1 File — (TGZ) [file pone.0159902.s009.tgz › Online_SI/graphics/lv3d.pdf]

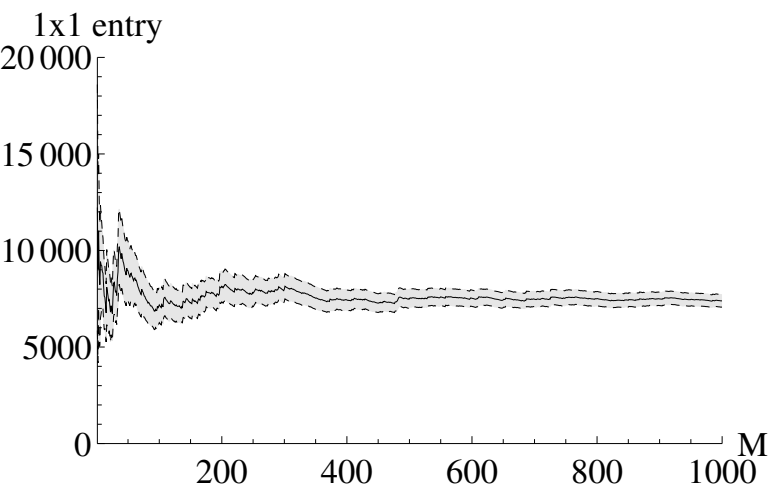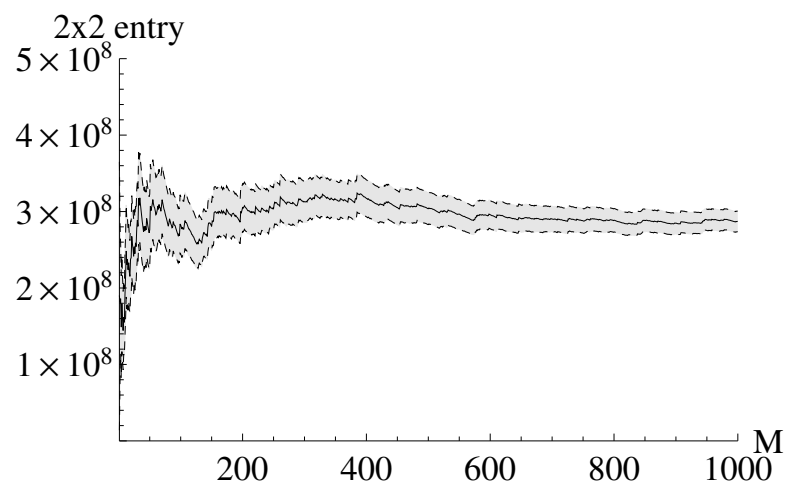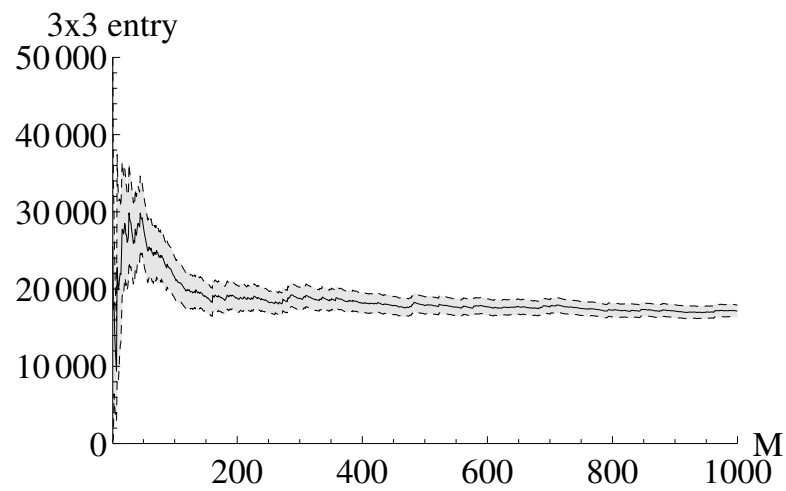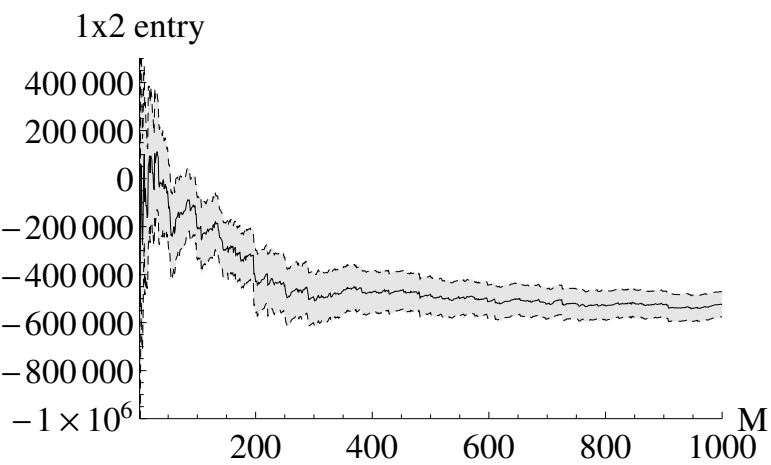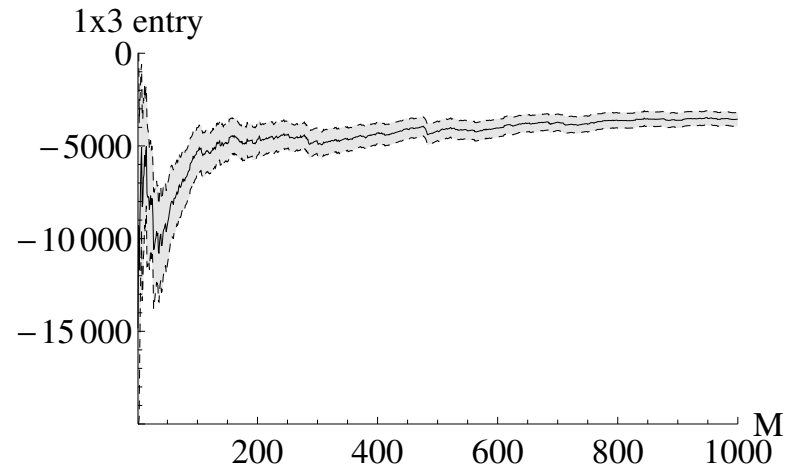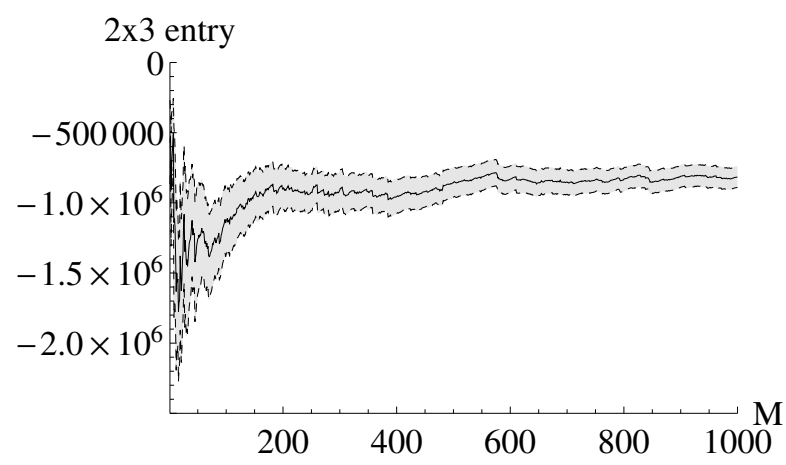

Supplement: S1 File — (TGZ) [file pone.0159902.s009.tgz › Online_SI/graphics/LVconv1.pdf]

B: partially observed

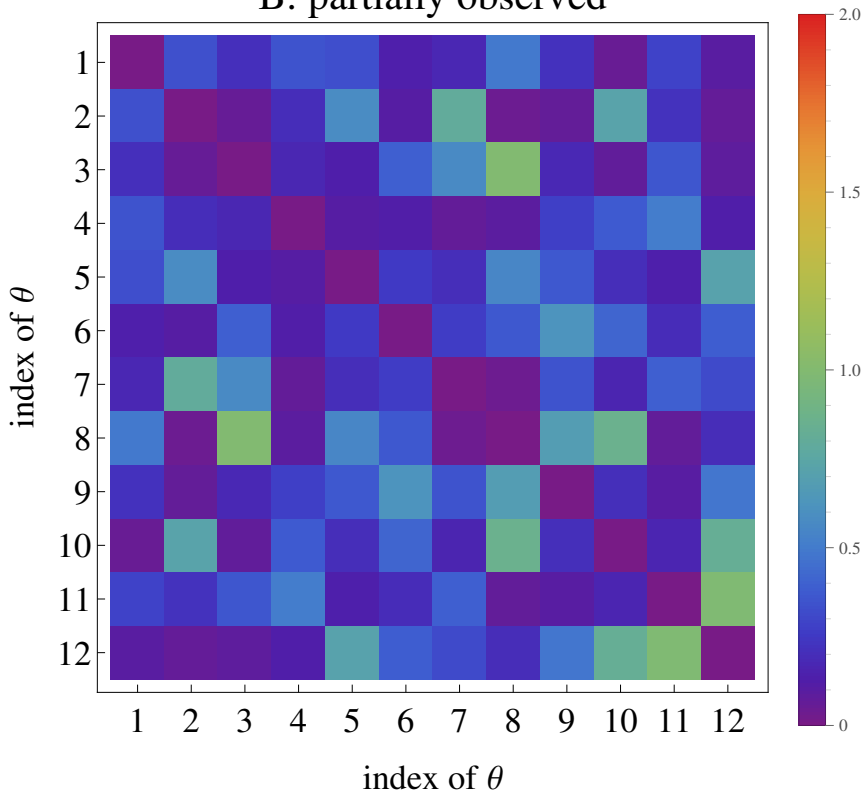

Supplement: S1 File — (TGZ) [file pone.0159902.s009.tgz › Online_SI/graphics/Capcorr.pdf]

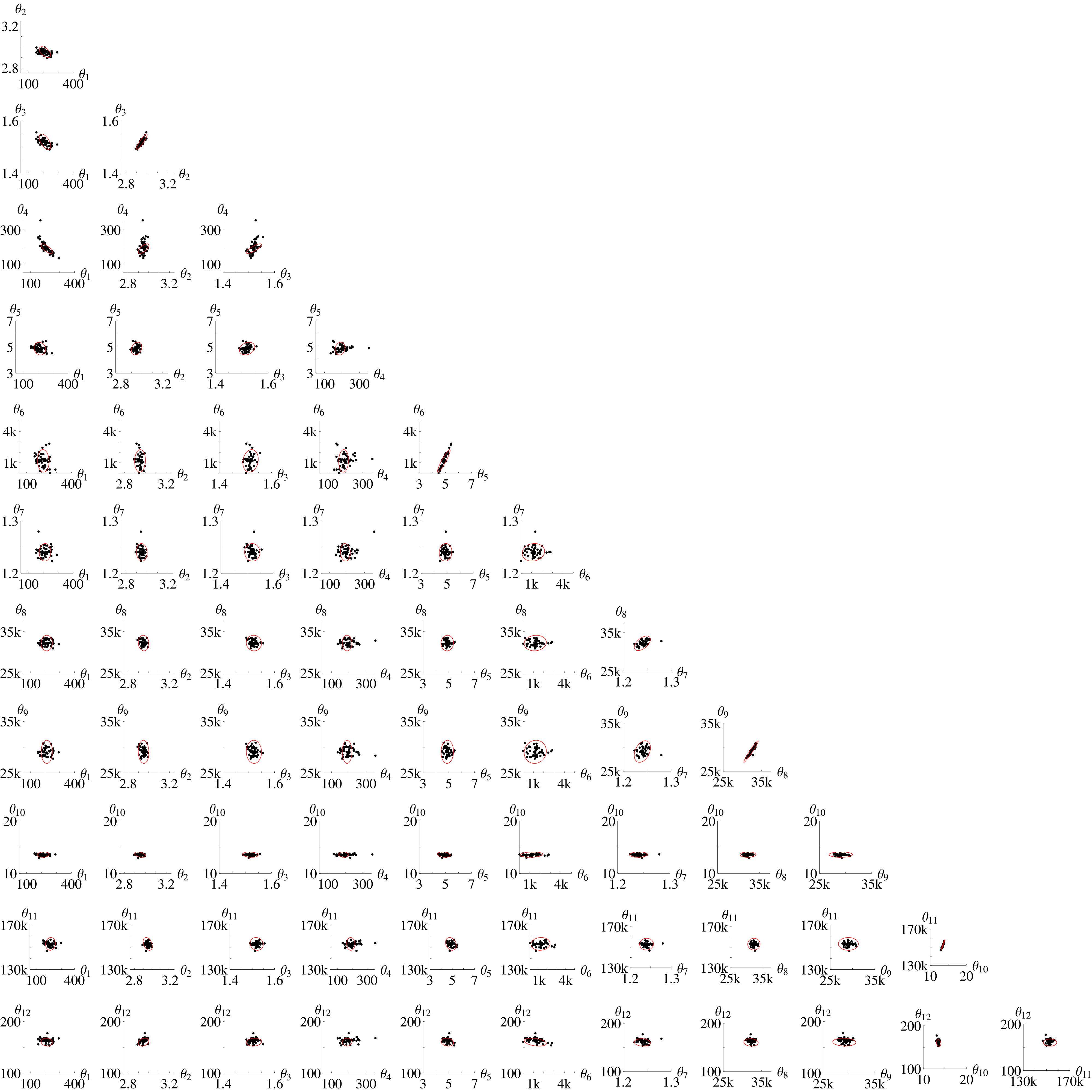

Supplement: S1 File — (TGZ) [file pone.0159902.s009.tgz › Online_SI/graphics/Ca2dsGillespie.pdf]

D-criterion

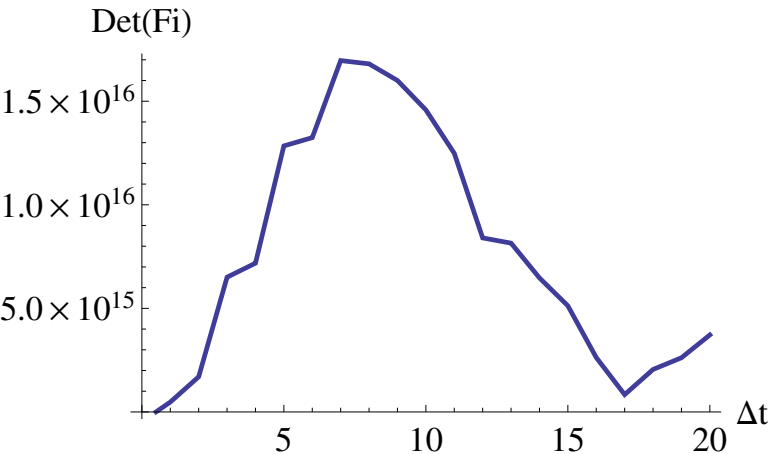

E-criterion

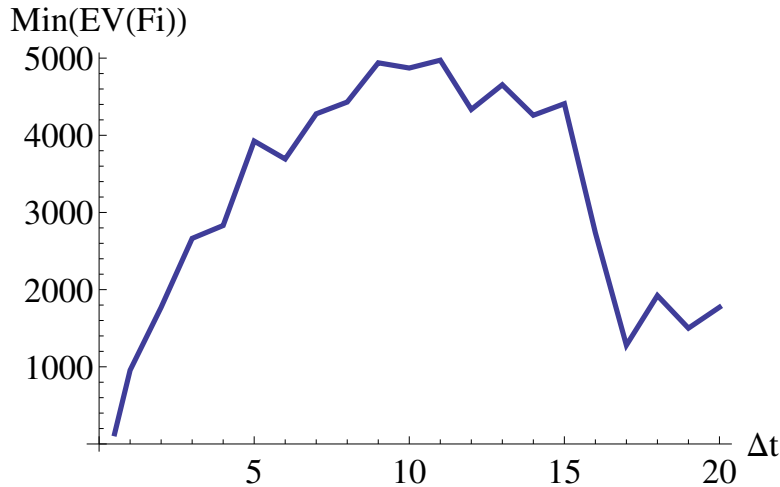

Supplement: S1 File — (TGZ) [file pone.0159902.s009.tgz › Online_SI/graphics/LVdtscan.pdf]

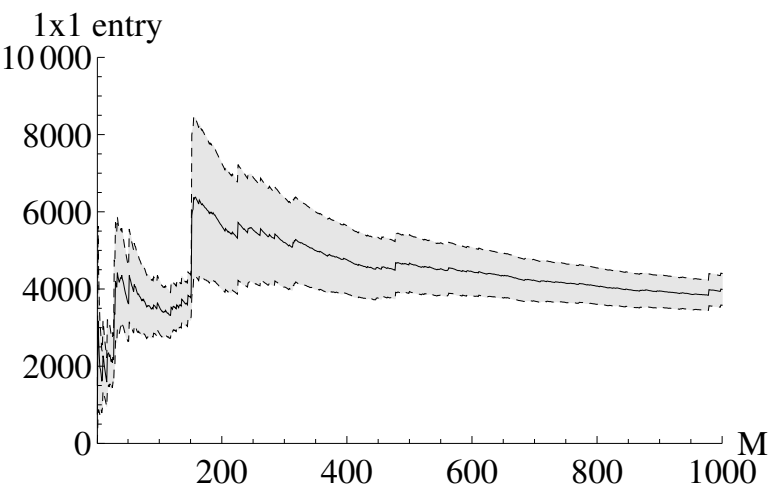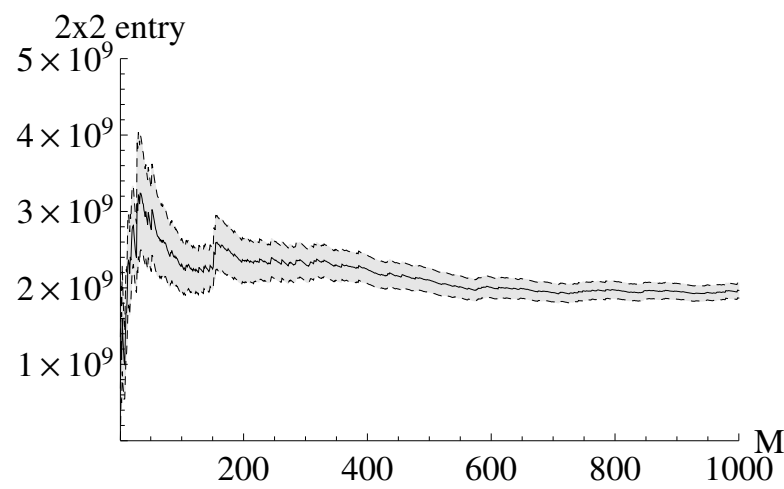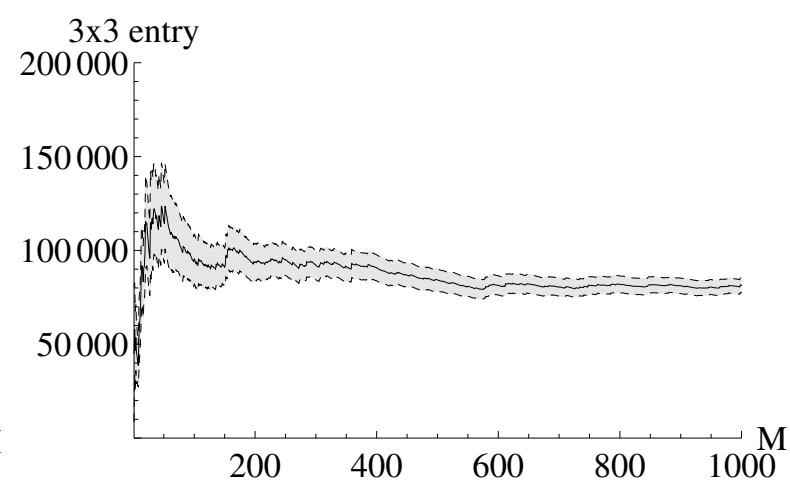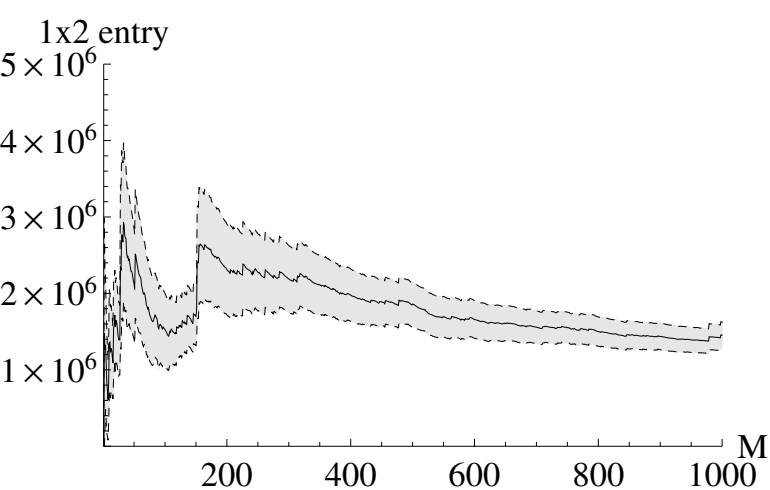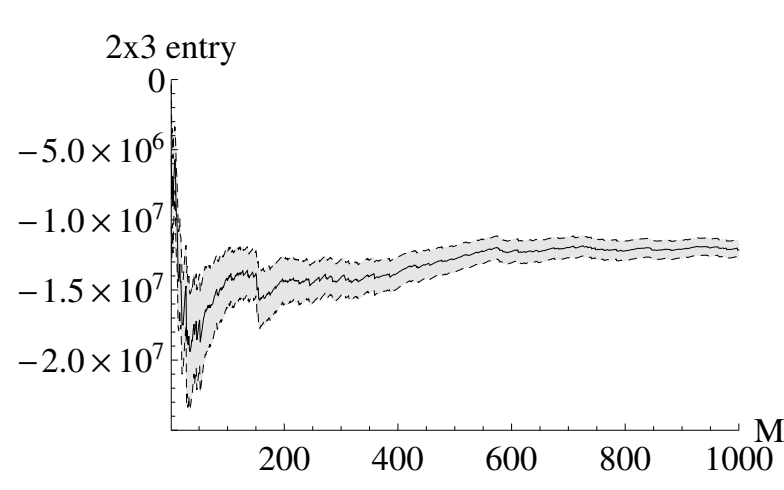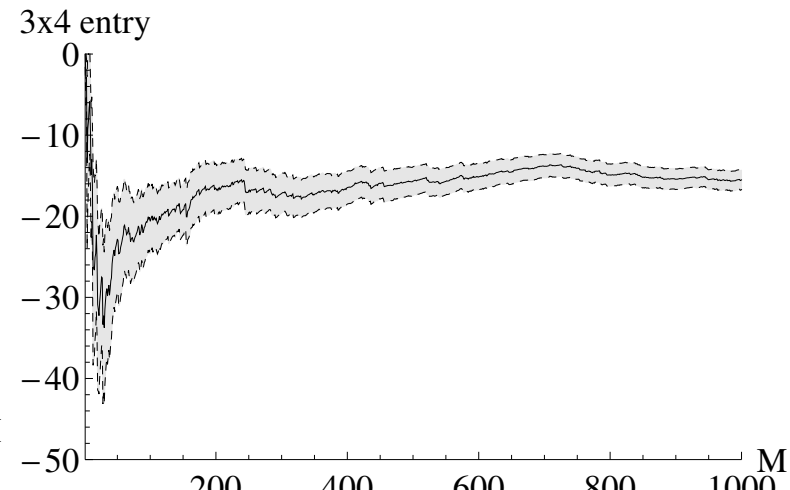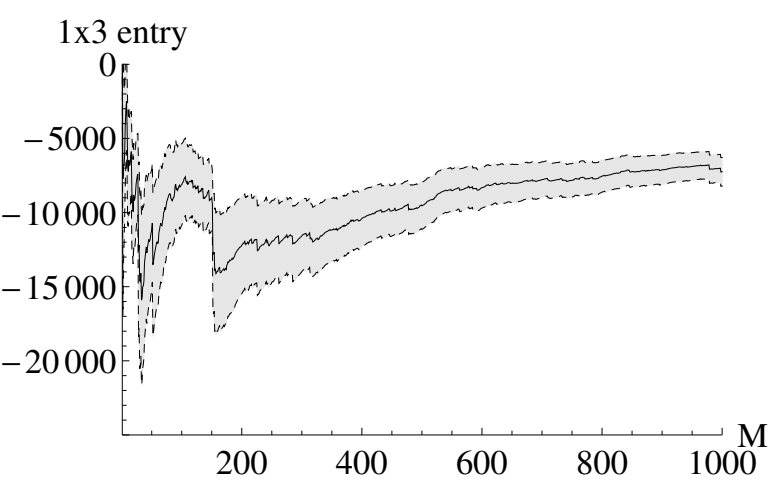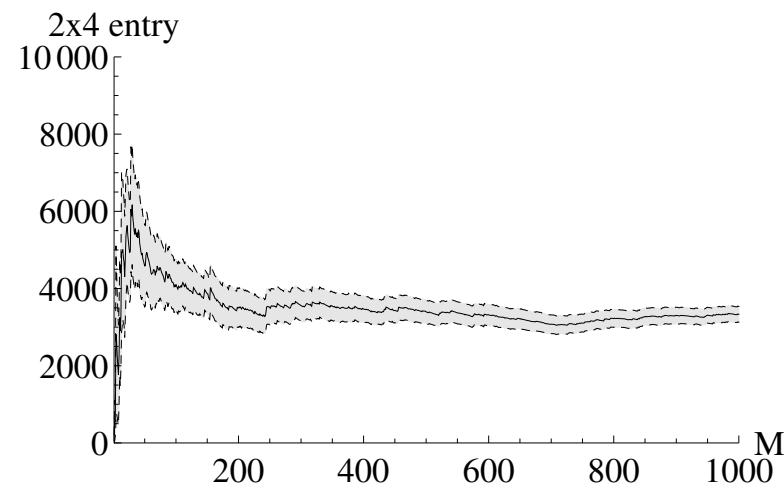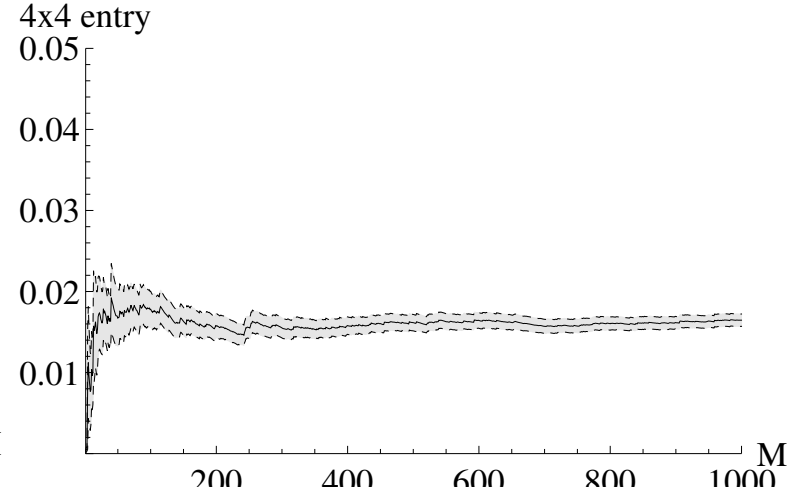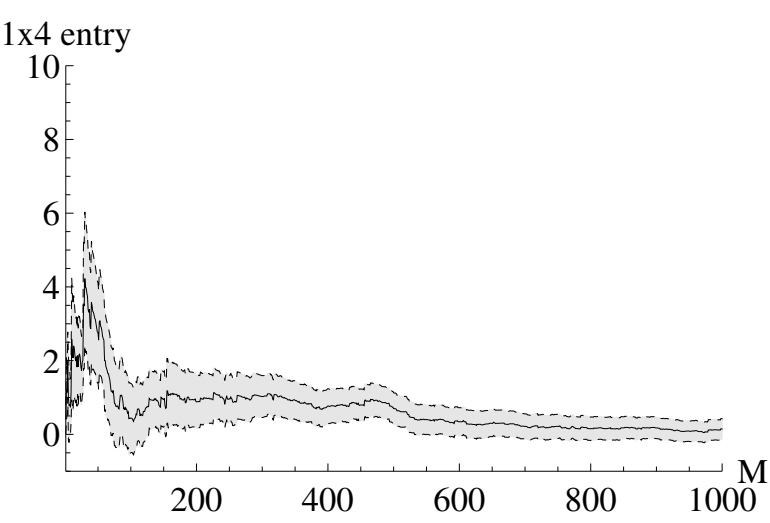

Supplement: S1 File — (TGZ) [file pone.0159902.s009.tgz › Online_SI/graphics/LVpconv.pdf]

D-criterion

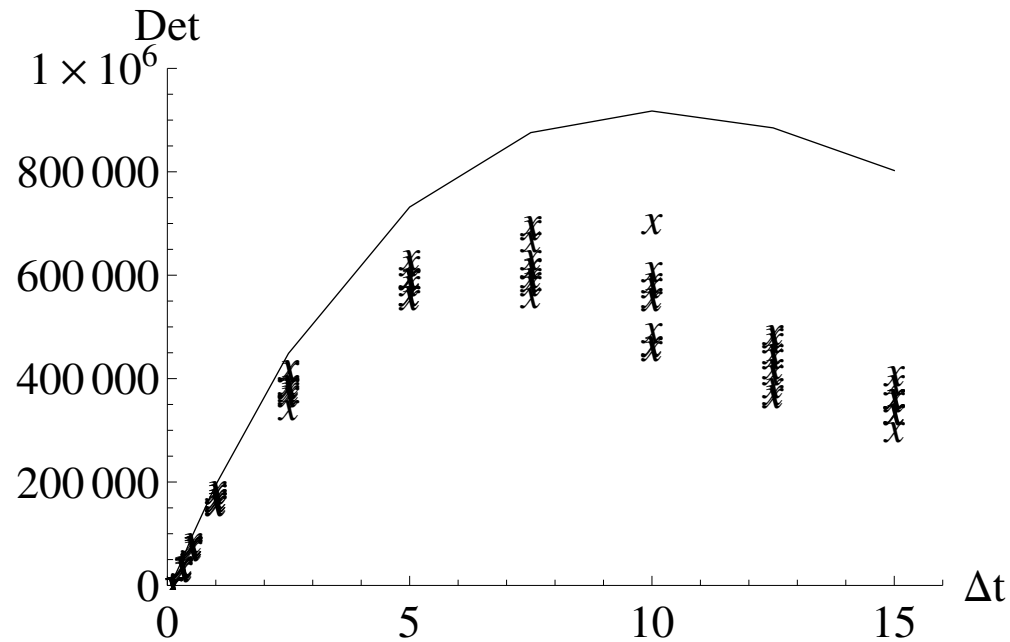

E-criterion

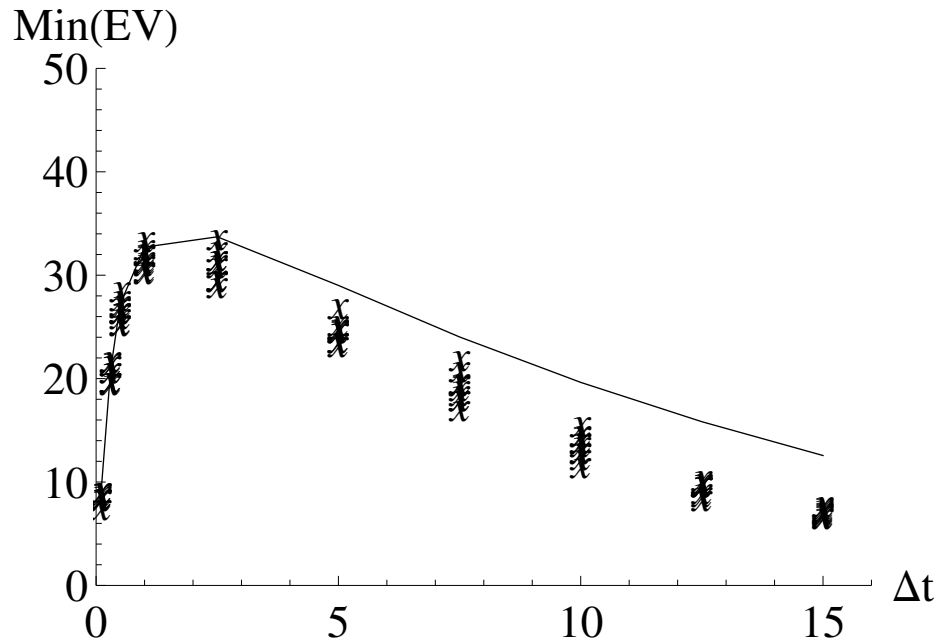

Supplement: S1 File — (TGZ) [file pone.0159902.s009.tgz › Online_SI/graphicsEXA/icrit10times.pdf]

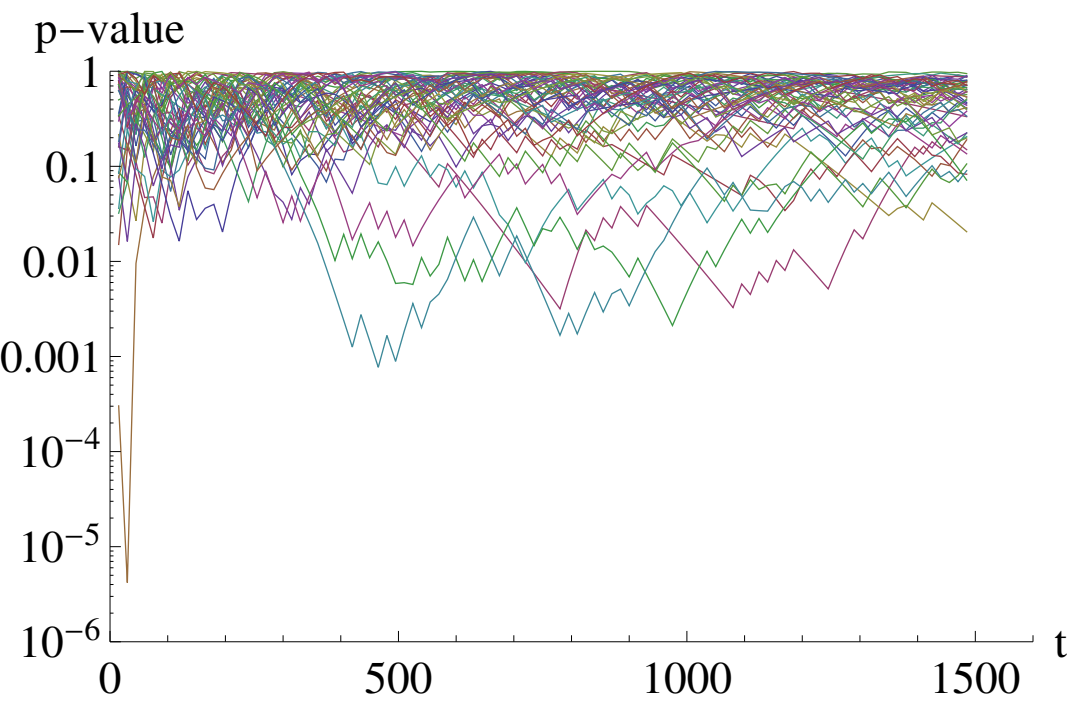

Supplement: S1 File — (TGZ) [file pone.0159902.s009.tgz › Online_SI/graphicsKOMOR/IDpvaluesKOMOR.pdf]

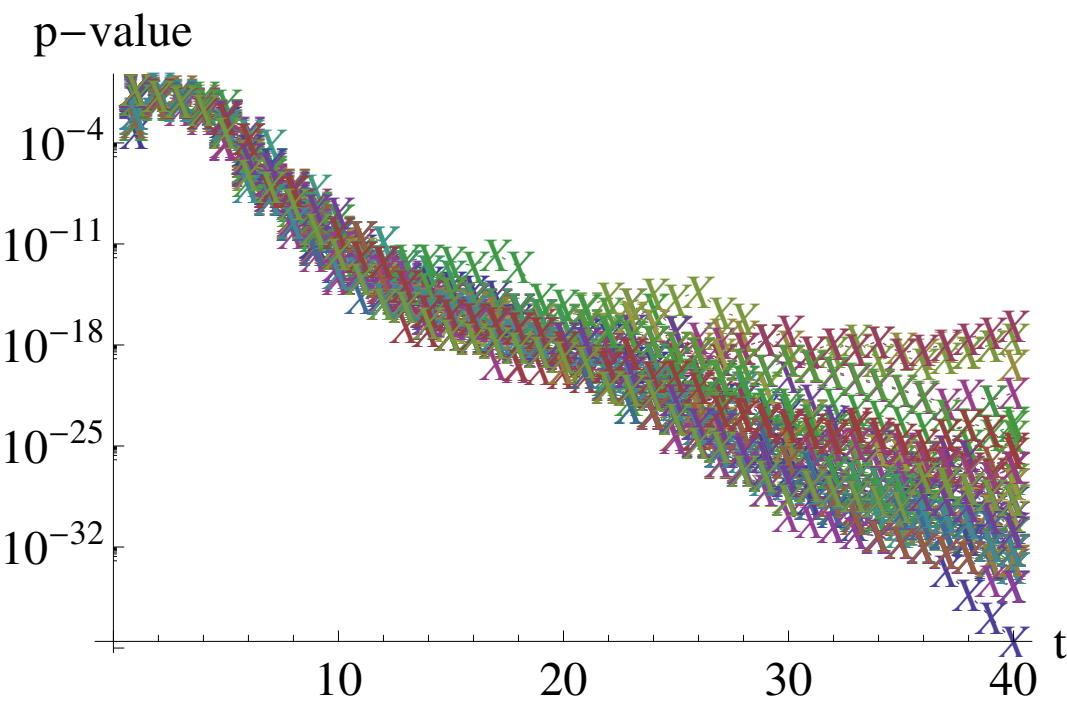

Supplement: S1 File — (TGZ) [file pone.0159902.s009.tgz › Online_SI/graphicsKOMOR/LVpvaluesKOMOR.pdf]

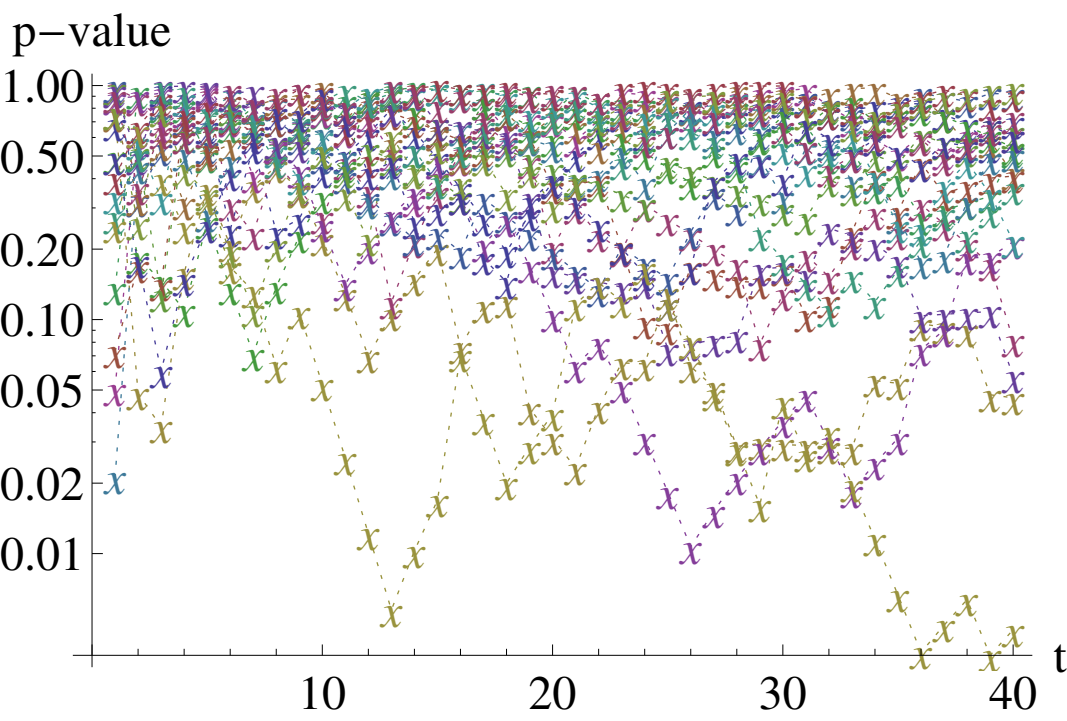

Supplement: S1 File — (TGZ) [file pone.0159902.s009.tgz › Online_SI/graphicsKOMOR/MSSpvalues.pdf]

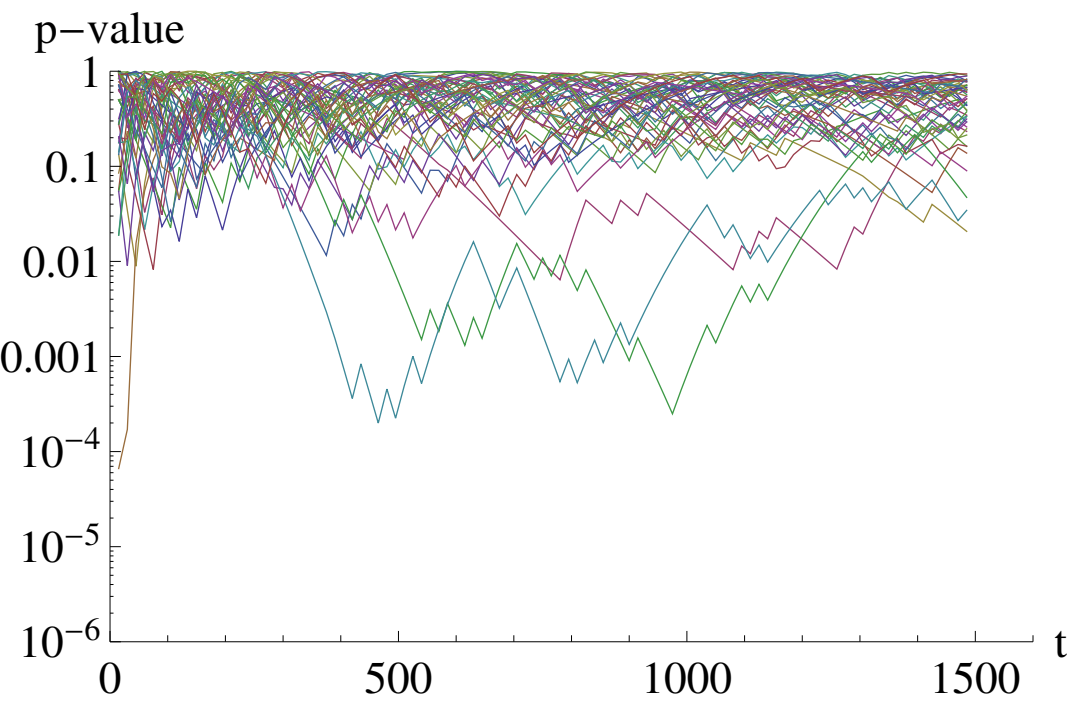

Supplement: S1 File — (TGZ) [file pone.0159902.s009.tgz › Online_SI/graphicsKOMOR/IDpvaluesMSS.pdf]

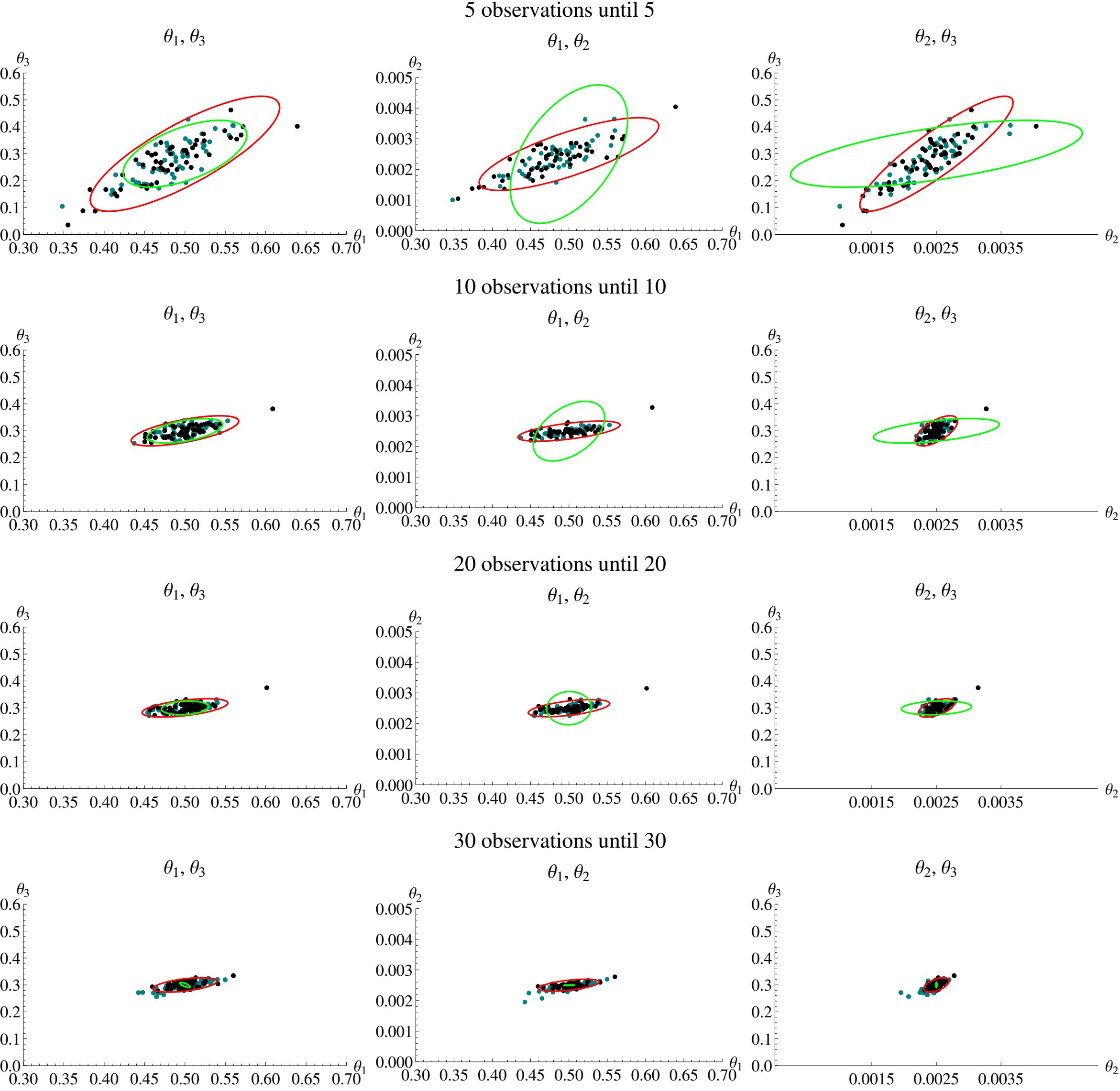

Supplement: S1 File — (TGZ) [file pone.0159902.s009.tgz › Online_SI/graphicsKOMOR/LV2dKOMOR.pdf]

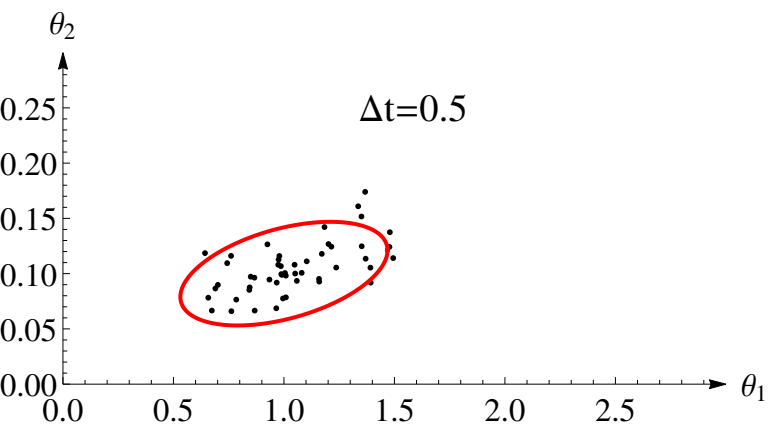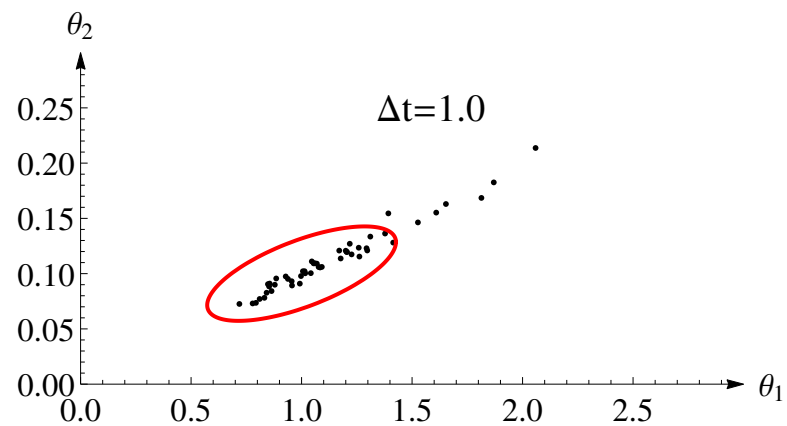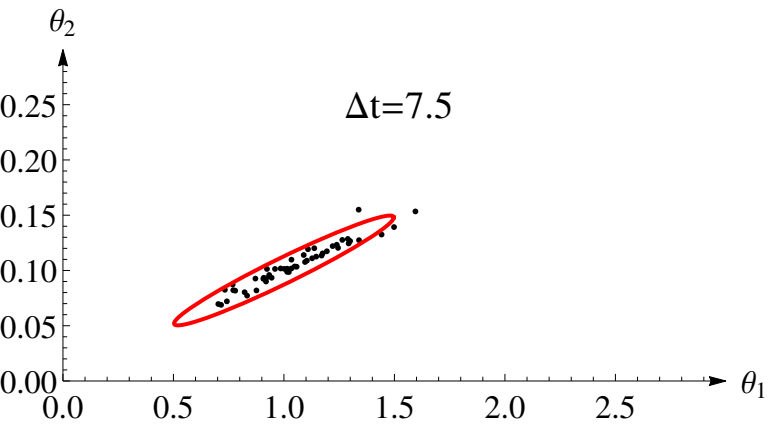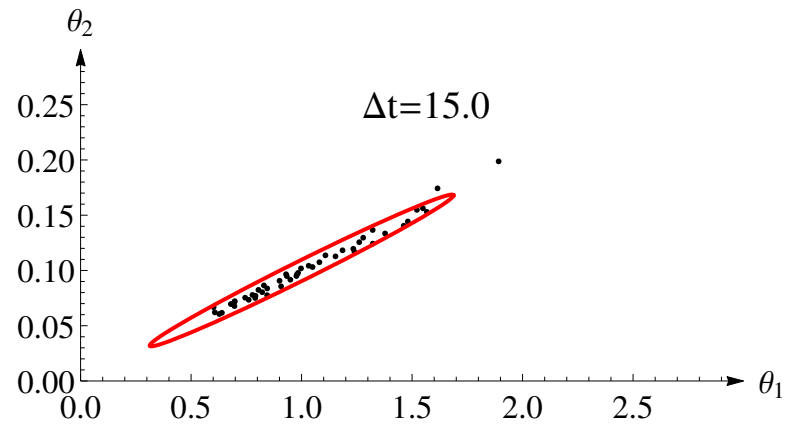

Supplement: S1 File — (TGZ) [file pone.0159902.s009.tgz › Online_SI/graphicsKOMOR/ID2dplotsEXA.pdf]

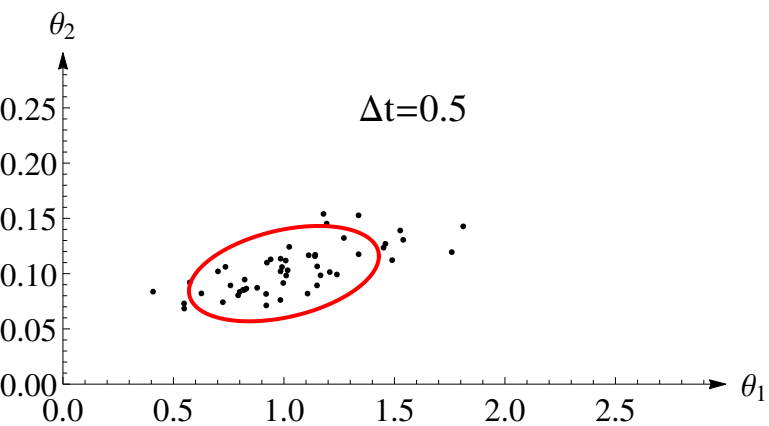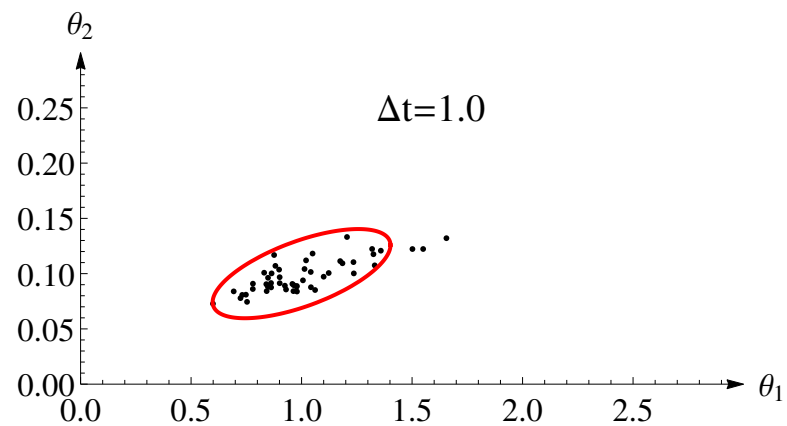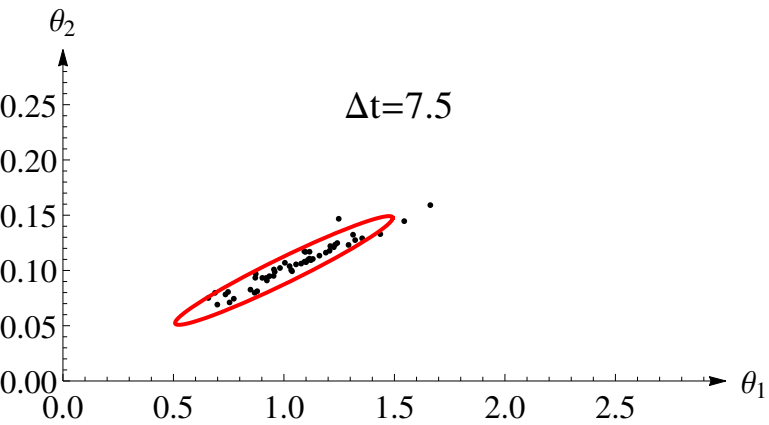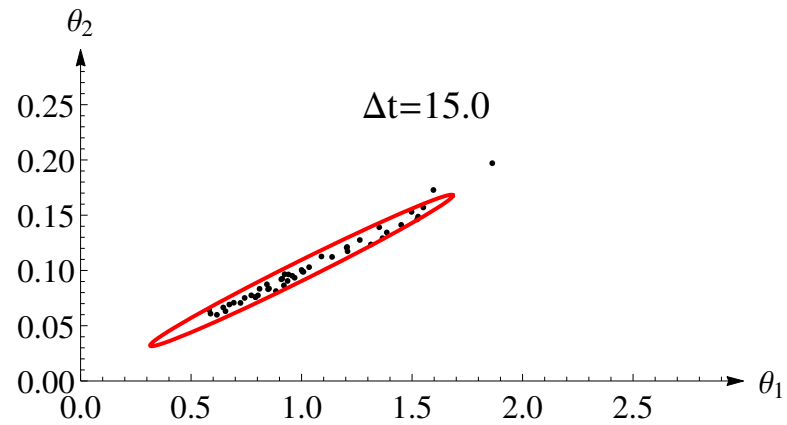

Supplement: S1 File — (TGZ) [file pone.0159902.s009.tgz › Online_SI/graphicsKOMOR/ID2dplotsKOMOR.pdf]

D-criterion

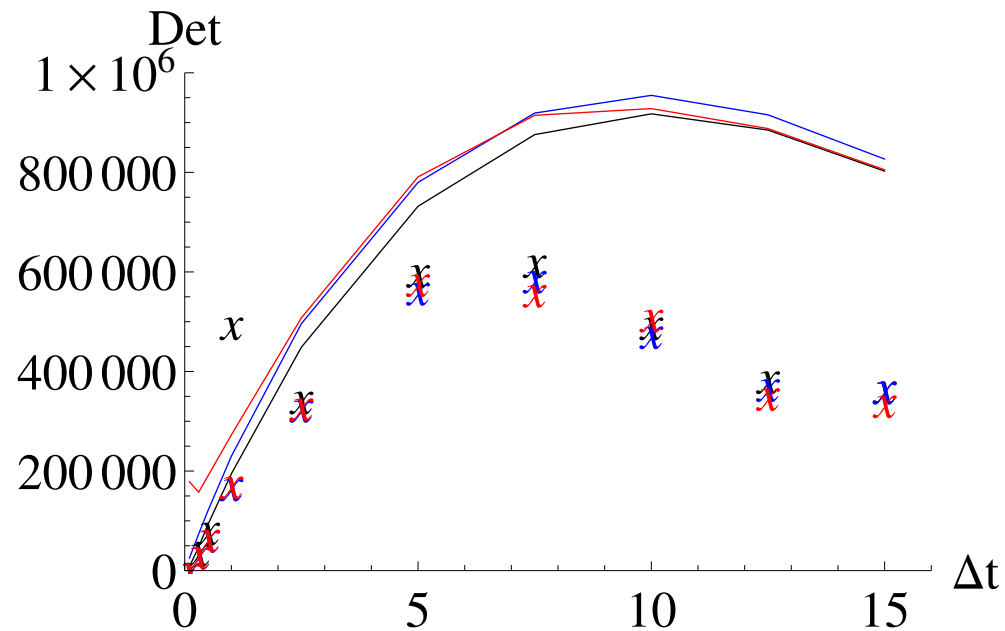

E-criterion

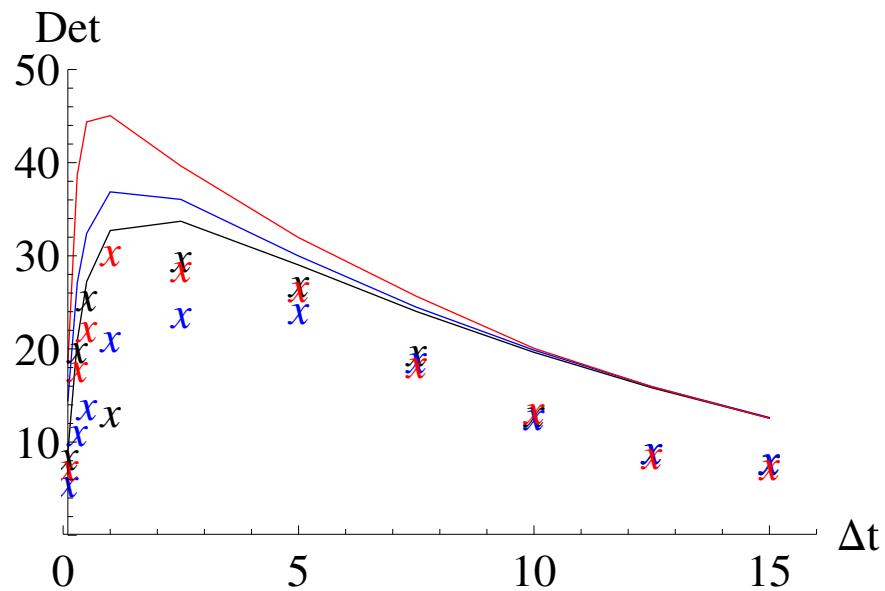

Supplement: S1 File — (TGZ) [file pone.0159902.s009.tgz › Online_SI/graphicsKOMOR/IDcritall3.pdf]

ARSE  $\theta_1$ 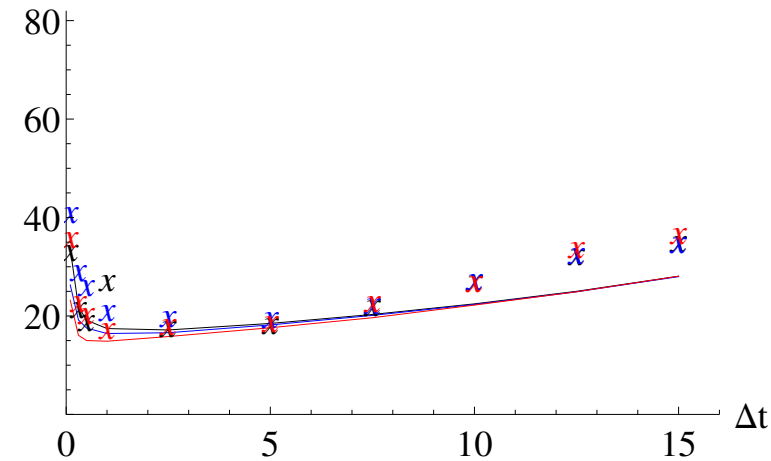ARSE  $\theta_2$ 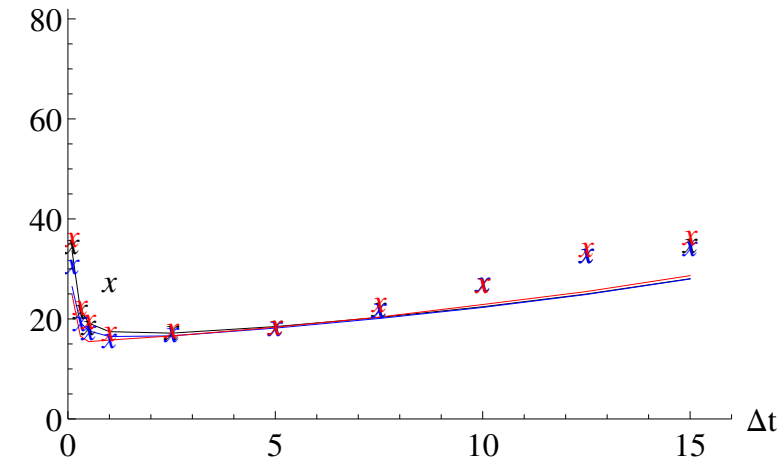

Supplement: S1 File — (TGZ) [file pone.0159902.s009.tgz › Online_SI/graphicsKOMOR/IDarseALL3.pdf]
