# Supplementary material for: Experimental Design for Stochastic Models of Nonlinear Signaling Pathways Using an Interval-Wise Linear Noise Approximation and State Estimation
Source: PLoS One. 2016 Sep 1;11(9):e0159902. doi: 10.1371/journal.pone.0159902 (PMC5008843; doi:10.1371/journal.pone.0159902)
Supplement: S1 File — (TGZ) [file pone.0159902.s009.tgz › Online_SI/graphics/LVexpdes-2d.pdf]

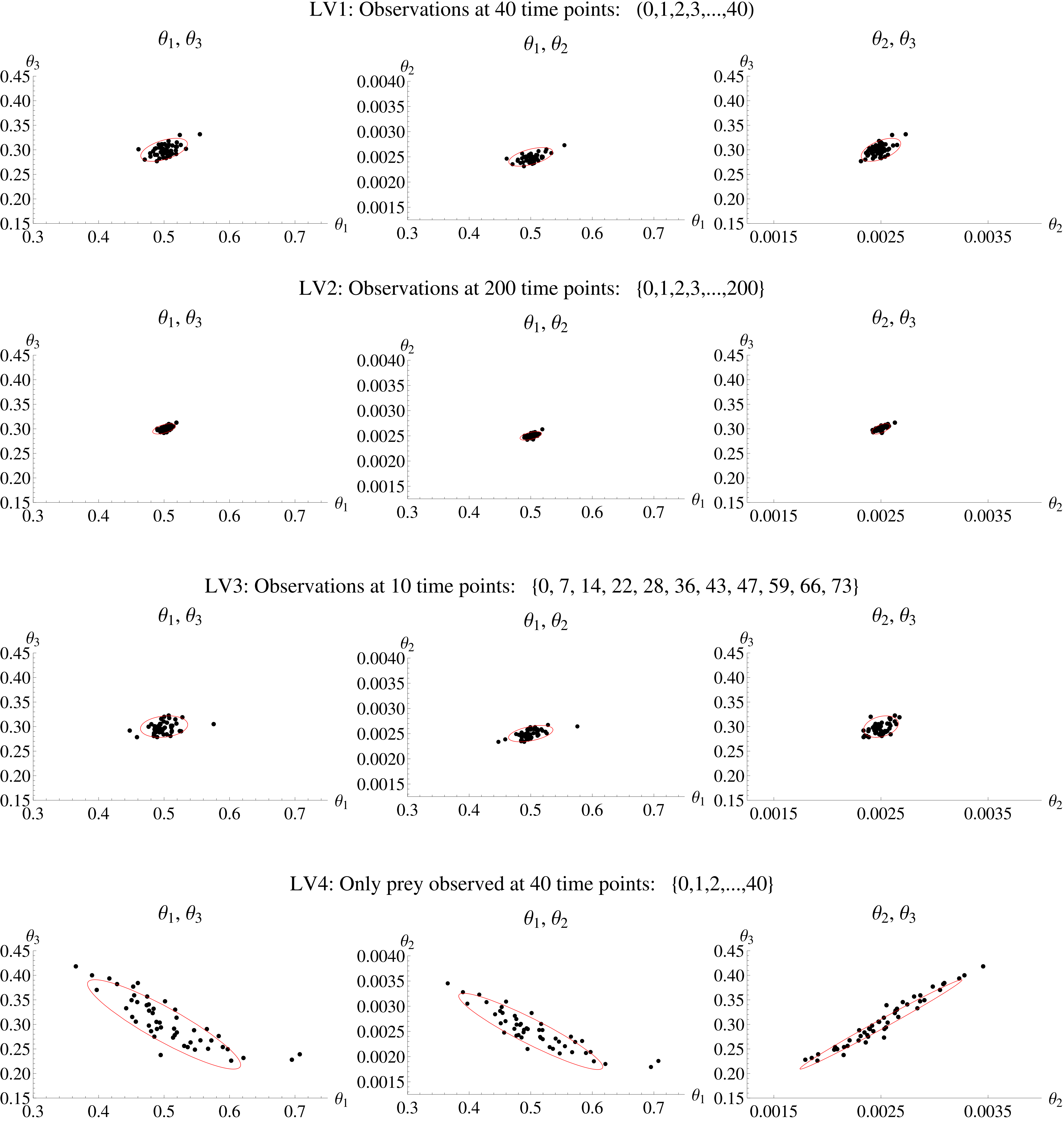

LV1: Observations at 40 time points: (0,1,2,3,...,40)
θ_1, θ_3
θ_1, θ_2
θ_2, θ_3
LV2: Observations at 200 time points: {0,1,2,3,...,200}
θ_1, θ_3
θ_1, θ_2
θ_2, θ_3
LV3: Observations at 10 time points: {0, 7, 14, 22, 28, 36, 43, 47, 59, 66, 73}
θ_1, θ_3
θ_1, θ_2
θ_2, θ_3
LV4: Only prey observed at 40 time points: {0,1,2,...,40}
θ_1, θ_3
θ_1, θ_2
θ_2, θ_3
θ_1
θ_2
θ_3
0.15
0.20
0.25
0.30
0.35
0.40
0.45
0.3
0.4
0.5
0.6
0.7
0.0015
0.0020
0.0025
0.0030
0.0035
0.0040
